# Gut microbiota does not play a mediating role in the causal association between inflammatory bowel disease and several its associated extraintestinal manifestations: a Mendelian randomization study

**DOI:** 10.3389/fimmu.2023.1296889

**Published:** 2024-01-15

**Authors:** Weipeng Lu, Jiepeng Cen, Qijie Dai, Heqing Tao, Liang Peng

**Affiliations:** Department of Gastroenterology, The Key Laboratory of Advanced Interdisciplinary Studies Center, The First Affiliated Hospital of Guangzhou Medical University, Guangzhou, Guangdong, China

**Keywords:** inflammatory bowel disease, gut microbiota, extraintestinal manifestations, mediating role, Mendelian randomization

## Abstract

**Background and objectives:**

Observational study has found inflammatory bowel disease to be associated with multiple extraintestinal manifestations. To this end, we characterized the causal association between inflammatory bowel disease and extraintestinal manifestations through a Mendelian randomization study and further explored the role of intestinal flora in inflammatory bowel disease and the extraintestinal manifestations associated with it.

**Materials and methods:**

We genetically predicted the causal relationship between inflammatory bowel disease and twenty IBD-related extraintestinal manifestations (including sarcoidosis, iridocyclitis, interstitial lung disease, atopic dermatitis, ankylosing spondylitis, psoriatic arthropathies, primary sclerosing cholangitis, primary biliary cholangitis). We used the full genome-wide association study (GWAS) summary statistics on gut microbiota in 18,340 participants from 24 cohorts to explore its role in the casual relationships between IBD and IBD-related extraintestinal manifestations. Inverse variance weighting (IVW) was used as the main analytical method to assess the causal associations. We performed Cochran’s Q test to examine the heterogeneity. To assess the robustness of the IVW results, we further performed sensitivity analyses including the weighted median method, MR-Egger regression, and Mendelian Randomization Pleiotropy RESidual Sum and Outlier (MR-PRESSO) test. The leave-one-out sensitivity analysis was further performed to monitor if significant associations were dominated by a single nucleotide polymorphism (SNP).

**Result:**

A total of eight extraintestinal manifestations were found to be at elevated risk of development due to inflammatory bowel diseases. A total of 11 causal relationships were found between IBD and gut microbiota, four of which were stable. Between gut microbiota and these eight extraintestinal manifestations, a total of 67 nominal causal associations were identified, of which 13 associations were stable, and notably 4 associations were strongly correlated.

**Conclusion:**

Through the two-sample MR analysis, we identified extraintestinal manifestations that were causally associated with inflammatory bowel disease and obtained multiple associations from inflammatory bowel disease and gut microbiota, and gut microbiota and extraintestinal manifestations in further analyses. These associations may provide useful biomarkers and potential targets for pathogenesis and treatment.

## 1 Introduction

Inflammatory bowel disease (IBD) is an autoimmune disease characterized by chronic inflammation of the gastrointestinal tract and primarily includes two subtypes: ulcerative colitis and Crohn’s disease (1). Previous studies have shown that patients with autoimmune diseases are often at a higher risk of developing other autoimmune conditions, a phenomenon also observed in IBD. Inflammatory bowel disease extends beyond intestinal inflammation and is frequently accompanied by various extraintestinal manifestations (2). Observational studies and meta-analyses have reported an increased risk of several extraintestinal diseases in IBD patients, including ankylosing spondylitis (3), psoriatic arthritis (3), psoriasis (3), iridocyclitis (3), primary sclerosing cholangitis (PSC) (3), primary biliary cholangitis (PBC) (3), rheumatoid arthritis (3), type 1 diabetes (3), asthma (3), acute pancreatitis (4), atopic dermatitis (5), cholelithiasis (6), deep vein thrombosis (7), pulmonary embolism (7), cerebrovascular disease (8), type 2 diabetes (9), multiple sclerosis (10), and interstitial lung disease, including its typical subtype, sarcoidosis (3, 11). Additionally, fatigue, a common yet often overlooked extraintestinal manifestation in IBD patients, is more prevalent and has higher fatigue scores during active periods compared to remission (12). Considering the potential confounding factors and reverse causality in observational studies, we aim to establish causal associations between IBD and the aforementioned 20 extraintestinal manifestations (EIMs) through a Mendelian randomization study.

The gut microbiota, a vast microbial community within the gastrointestinal tract, plays a crucial role in bile acid metabolism (13), short-chain fatty acid (SCAF) synthesis (14), synthesis of nutrients (15), and modulation of the host immune system (16). Thus, maintaining the stability of the gut microbiota is of significant importance for overall human health. As active participants in various associations such as the “gut–liver axis,” “gut–lung axis,” and “gut–skin axis,” the gut microbiota contributes to the development of various extraintestinal diseases through mechanisms like bacterial translocation (17) and molecular mimicry (18). Under normal circumstances, a structurally and functionally intact gut barrier serves as an effective defense mechanism against pathogenic gut bacteria. However, in the case of IBD, the integrity of the gut barrier is compromised, providing a conducive environment for the aforementioned mechanisms to occur (19). Existing studies have demonstrated differences in gut microbiota composition between individuals with IBD and healthy individuals, as well as in several extraintestinal manifestations associated with IBD (20, 21). These findings suggest that the gut microbiota may play a role in both IBD and its extraintestinal manifestations. However, based on current research, we cannot determine whether changes in the gut microbiota are accompanying variations or whether they play a key mediating role in the development of IBD and its extraintestinal manifestations. Epidemiological studies are limited by inherent biases, making it difficult to establish causal relationships in the disease process.

Mendelian randomization (MR) assesses the causal effect of exposure factors on outcomes by using the genetic variation that is randomly allocated at conception as the instrumental variables (IVs) (22). Since IVs are arranged randomly in the process of fertilization and were unaffected by environmental factors and lifestyle, thus MR analyses decreases residual confounding. Moreover, due to IVs being determined irrespective of disease progression or development, MR analyses are more advantageous in judging the directionality of causality.

Our study applied genome-wide association statistics (GWAS) data and we conducted a Mendelian randomization study to further explore the potential mediating role of gut microbiota abundance in the development of IBD and its associated extraintestinal manifestations.

## 2 Method

### 2.1 Study design

**Figure 1:**
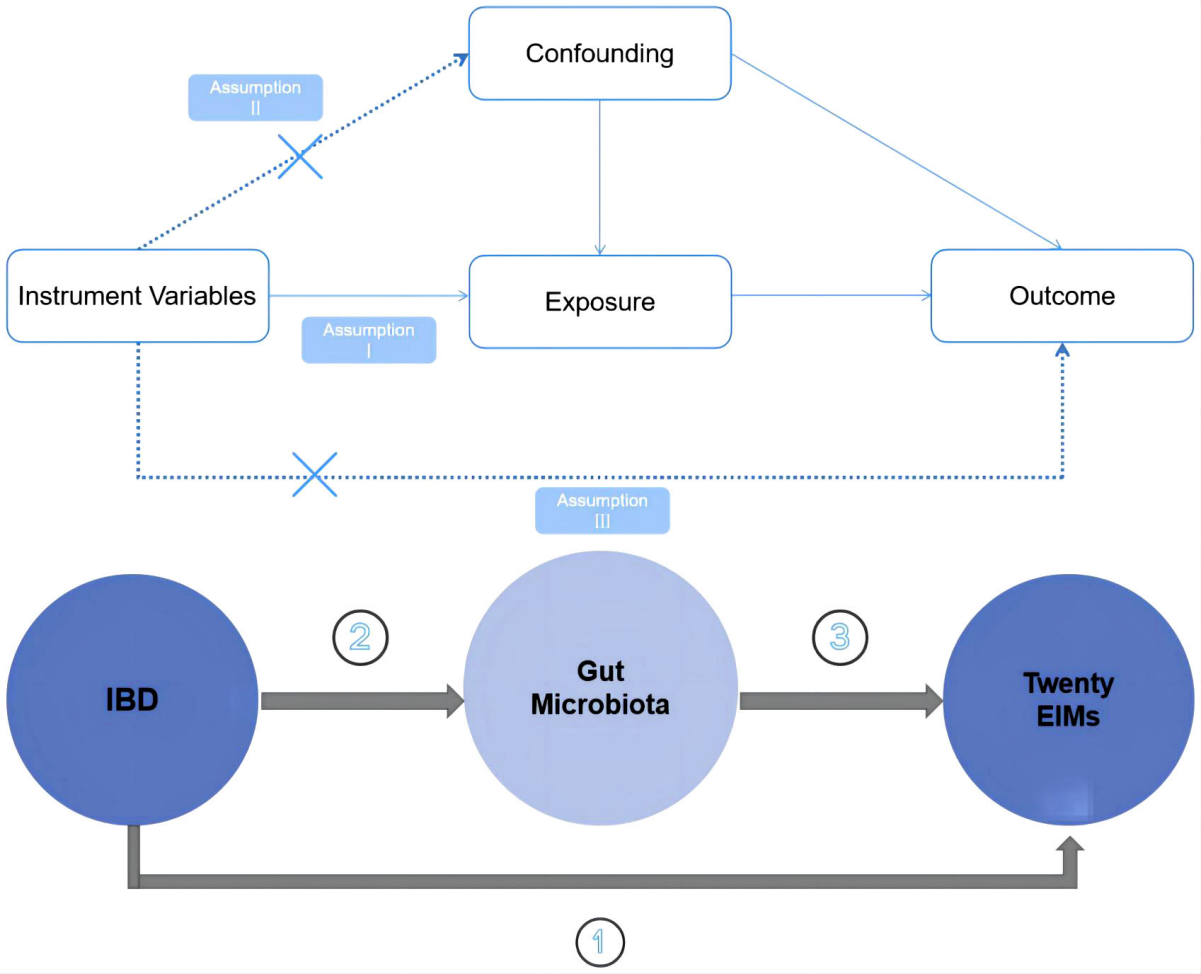
Schematic diagrams illustrating the study design.

The overall study design is illustrated in **Figure 1**. Specifically, we employed a two sample MR approach. To minimize bias and ensure the robustness of the results, we focused on satisfying three key assumptions inherent to the MR methodology. Firstly, we ensured that the instrumental variables (IVs) used in the analysis were significantly associated with gut microbiota.

Secondly, we took great care to select IVs that were independent, meaning they were not associated with any other confounding factors. Thirdly, we examined whether the selected IVs specifically affected the outcome of interest through the exposure factors under investigation and not through alternative pathways. By scrutinizing these pathways, we aimed to ensure that the observed effects were truly attributable to the impact of gut microbiota abundance on the outcome of interest.

### 2.2 Data source and instruments

#### 2.2.1 Human gut microbiome

The full GWAS summary statistics of the microbiota were derived primarily from a large-scale multi-ethnic GWAS meta-analysis (MiBioGen Consortium, www.mibiogen.org (accessed on 15 July 2022)) of 18,340 people from 24 cohorts which recorded 211 gut microbiota and 122,110 related single nucleotide polymorphisms (SNPs) (dataset citations can be seen in **Supplementary Table S1**). We removed 15 bacterial traits without specific species names (unknown family or genus), leaving 196 bacterial traits. We selected IVs at P< 1×10^-5^ to obtain a more comprehensive result and performed LD-clumping for all the IVs (r^2^< 0.001, distance = 10,000kb) to reduce the influence of correlations between SNPs. To avoid weak instrument bias, we only included SNPs with an F-statistic greater than 10. To ensure that each IV was associated with the same effect allele, we coordinated the summary statistics and eliminated the palindromic SNPs.

#### 2.2.2 IBD and IBD-related EIMs

The summary statistics of instrument variables for IBD were derived from a GWAS meta-analysis study of IBD from the International Inflammatory Bowel Disease Genetics Consortium (IIBDGC) which includes 31,665 cases and 33,977 controls with a total of 157,116 SNPs (data link: https://gwas.mrcieu.ac.uk/datasets/ieu-a-294/). Briefly, the genetic association data consisted of 33,394 individuals (N = 10,588 cases, 22,806 controls) with psoriasis, 24,751 individuals (N = 4,796 cases, 19,955 controls) with PSC, 115,803 individuals (N = 47,429 cases, 68,374 controls) with multiple sclerosis, 29,652 individuals (N = 6,683 cases, 12,173 controls) with type 1 diabetes, 408,442 individuals (N = 56,167 cases, 352,255 controls) with asthma, 13,239 individuals (N = 2,764 cases, 10,475 controls) with PBC, and 58,284 individuals (N = 14,361 cases, 43,923 controls) with rheumatoid arthritis. We obtained genetic associations for deep venous thrombosis published online by the MRC-IEU (https://gwas.mrcieu.ac.uk/datasets/ukb-b-12040/). The GWAS summary statistics of 12 other EIMs including ankylosing spondylitis, psoriatic arthropathies, atopic dermatitis, iridocyclitis, sarcoidoses, pulmonary embolism, cerebrovascular disorder, type 2 diabetes, interstitial lung disease, malaise and fatigue, cholelithiasis, and acute pancreatitis were accessed online (dataset citations can be seen in **Supplementary Table S1**). We selected SNPs with P-values less than the locus-wide significance level (5 × 10^-8^) and excluded instrumental variables with F-values< 10 to ensure the strength of association between instrumental variables and the exposure. To exclude horizontal pleiotropy and satisfy the independence test, we set the linkage disequilibrium parameter (r2) for SNPs to 0.001, the genetic distance to 10,000 kb, and those with minor allele frequency (MAF) values less than 0.01 were also excluded.

### 2.3 Statistical analysis

First, we used inverse variance weighting (IVW) as the main analytical method to assess the causal associations of IBD on IBD-related EIMs, IBD on gut microbiota, and gut microbiota on IBD-related EIMs. Then, to examine the heterogeneity of each SNP, we performed Cochran’s Q test. If significant heterogeneity was observed (p< 0.05), the IVW random-effects model was used, otherwise the IVW fixed-effects model would be used. To assess the robustness of the IVW results, we further performed sensitivity analyses including the weighted median method, MR-Egger regression, the Mendelian Randomization Pleiotropy RESidual Sum and Outlier (MR-PRESSO) test, and the leave-one-out sensitivity analysis monitoring if significant associations were dominated by a single SNP. Additionally, we also calculated the F-value (formula: $\text{F }=\;R^{2}\;\left(\text{n}-\text{k}-1\right)/\text{k}\left(1-R^{2}\;\right)$ ). SNPs with an F value< 10 were regarded as having weak instrumental variable bias and, therefore, were excluded from the study. Furthermore, the MR Steiger test of directionality was used to detect whether the assumption that exposure causes outcome was valid, and if not, it was rejected.

We performed two-sample Mendelian randomization analyses for three main groups of relationships: (1) IBD and IBD-related extraintestinal manifestations, (2) IBD and gut microbiota, and (3) gut microbiota and IBD-related extraintestinal manifestations confirmed to be causally associated with IBD by two sample MR. In the two Mendelian randomization analyses involving intestinal flora, we assessed the potential association between gut microbiota and the risk of IBD or IBD-related extraintestinal manifestations. A significance threshold of P< 0.05 was considered indicative of nominal significance. Additionally, when any specific intestinal flora demonstrated presence in both IBD and IBD-related extraintestinal manifestations, we further calculated its mediating effect value (citations of specific methods can be seen in **Supplementary Table S2**).

To obtain a more rigorous interpretation of causality, we also used Bonferroni correction according to each attribute (genus: 0:005/131 (3.81×10^-4^), family: 0.05/35 (1.4 × 10^-3^), order: 0.05/20 (2.5 × 10^-3^), class: 0.05/16 (3.1×10^-3^), and phylum: 0.05/9 (5.5×10^-3^)). If a significance threshold of P< Bonferroni corrected P-value, it indicates a strong causal relationship. All MR analyses were performed in R (version 4.3.0) using the “TwosampleMR” and “MR-PRESSO” packages.

## 3 Results

### 3.1 MR (IBD and IBD-related extraintestinal manifestations)

**Figure 2:**
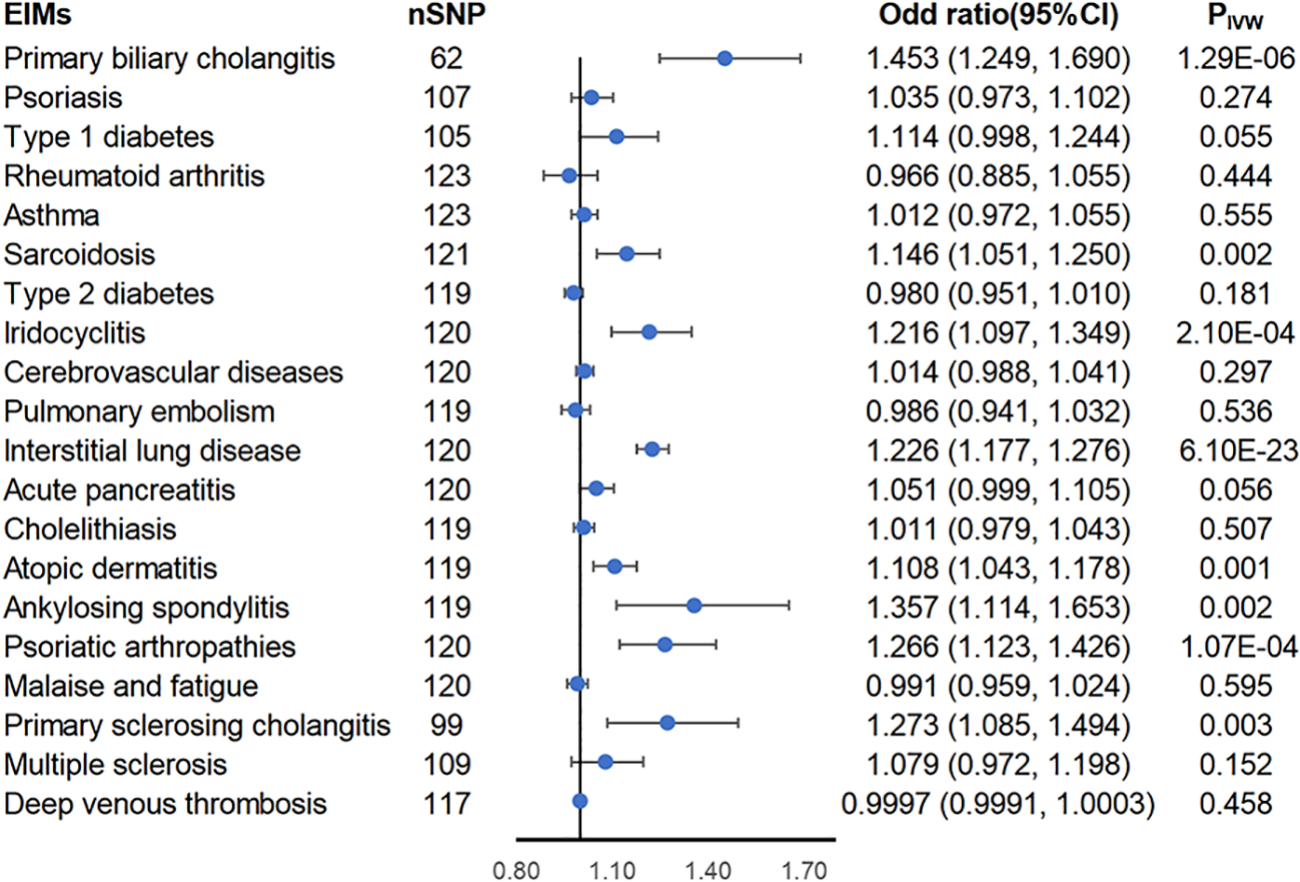
Forest plot of estimates from genetically predicted IBD on IBD-related extraintestinal manifestations. Presented are extraintestinal manifestations that were statistically significant using the inverse variance weighted (IVW) method.

Using the two-sample MR approach, genetically predicted liability to inflammatory bowel diseases was associated with higher risks of PBC (OR_IVW_ = 1.453; 95% CI = 1.249-1.69; P_IVW_ = 1.29 × 10^-6^), sarcoidosis (OR_IVW_ = 1.146; 95% CI = 1.051-1.25; P_IVW_ = 0.002), iridocyclitis (OR_IVW_ = 1.216; 95% CI = 1.097-1.349; P_IVW_ = 0.0002), interstitial lung disease (OR_IVW_ = 1.226; 95% CI = 1.177-1.276; P_IVW_ = 6.1× 10^-23^), atopic dermatitis (OR_IVW_ = 1.108; 95% CI = 1.043-1.178; P_IVW_ = 0.001), ankylosing spondylitis (OR_IVW_ = 1.357; 95% CI = 1.114-1.653; P_IVW_ = 0.002), psoriatic arthropathies (OR_IVW_ = 1.266; 95% CI = 1.123-1.426; P_IVW_ = 0.0001), and PSC (OR_IVW_ = 1.273; 95% CI = 1.085-1.494; P_IVW_ = 0.003) (**Figure 2**). Heterogeneity was detected in all of these eight causal associations via Cochran’s Q statistics test (p< 0.05), thus using the random-effects model, and under both the fixed-effects and random-effects models, the results are equally positive or equally negative (**Supplementary Figure S5**). No horizontal pleiotropy was observed in the remaining seven causal associations by MR-Egger intercept (p > 0.05) except for PBC (**Supplementary Table S1**). However, the MR-PRESSO global test, another sensitivity analysis method, found significant horizontal pleiotropy in the eight causal relationships mentioned above. After distorting outliers using the MR-PRESSO distortion test, there was no significant difference between the results before and after correction of the outliers except for ankylosing spondylitis (distortion test p value< 0.05) (**Supplementary Table S1**). Furthermore, the leave-one-out method indicated no SNP dominated the positive results above (**Supplementary Figure S2**). Therefore, we next further explored whether the flora plays a mediating role in these eight causal associations.

### 3.2 MR (IBD and gut microbiota)

**Figure 3:**
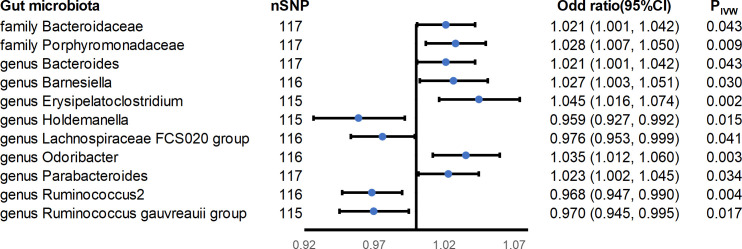
Forest plot of estimates from genetically predicted IBD on gut microbiota. Presented are the gut microbiota that were statistically significant using the inverse variance weighted (IVW) method.

Eleven causal associations were found to reach nominal significance (p< 0.05) in this study (**Figure 3**). A higher genetically predicted IBD was associated with increased abundance of the family Bacteroidaceae (OR_IVW_ = 1.021; 95% CI = 1.001-1.042; P_IVW_ = 0.043), family Porphyromonadaceae (OR_IVW_ = 1.028; 95% CI = 1.007-1.050; P_IVW_ = 0.009), genus Bacteroides (OR_IVW_ = 1.021; 95% CI = 1.001-1.042; P_IVW_ = 0.043), genus Barnesiella (OR_IVW_ = 1.027; 95% CI = 1.003-1.051; P_IVW_ = 0.03), genus Erysipelatoclostridium (OR_IVW_ = 1.045; 95% CI = 1.016-1.074; P_IVW_ = 0.002), genus Odoribacter (OR_IVW_ = 1.035; 95% CI = 1.012-1.060; P_IVW_ = 0.003), and genus Parabacteroides (OR_IVW_ = 1.023; 95% CI = 1.001-1.045; P_IVW_ = 0.034), while a higher risk of IBD was associated with decreased abundance of the genus Holdemanella (OR_IVW_ = 0.959; 95% CI = 0.927-0.992; P_IVW_ = 0.015), genus Lachnospiraceae FCS020 group (OR_IVW_ = 0.976; 95% CI = 0.953-0.999; P_IVW_ = 0.041), genus Ruminococcus 2 (OR_IVW_ = 0.968; 95% CI = 0.947-0.990; P_IVW_ = 0.004), and genus Ruminococcus gauvreauii group (OR_IVW_ = 0.970; 95% CI = 0.945-0.995; P_IVW_ = 0.017). All the results had no significant heterogeneity and horizontal pleiotropy to be found from the Cochrane’s Q, MR-Egger, and MR-PRESSO tests (**Supplementary Table S2**). In addition, the leave-one-out method showed that except for the genus Erysipelatoclostridium, genus Holdemanella, genus Odoribacter, genus Ruminococcus 2, and genus Ruminococcus gauvreauii group, some SNPs might dominate the positive results (**Supplementary Figure S7**).

### 3.3 MR (Gut microbiota and IBD-related EIMs)

#### 3.3.1 Sarcoidosis

**Figure 4:**
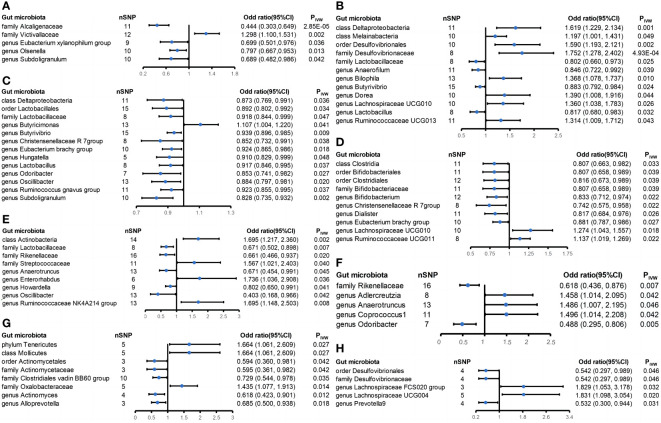
Forest plot of estimates from genetically predicted gut microbiota on extraintestinal manifestations. Presented are the gut microbiota that were statistically significant using the inverse variance weighted (IVW) method. **(A)** Sarcoidosis; **(B)** iridocyclitis; **(C)** interstitial lung disease; **(D)** atopic dermatitis; **(E)** ankylosing spondylitis; **(F)** psoriatic arthropathies; **(G)** primary sclerosing cholangitis; and **(H)** primary biliary cholangitis.

There were five casual relationships found in this study (**Figure 4A**). A higher genetically predicted family Victivallaceae (OR_IVW_ = 1.298; 95% CI = 1.100-1.531; P_IVW_ = 0.002) was associated with higher risk of sarcoidosis while the family Alcaligenaceae (OR_IVW_ = 0.444; 95% CI = 0.303-0.649; P_IVW_ = 2.85 × 10^-5^), genus Eubacterium xylanophilum group (OR_IVW_ = 0.699; 95% CI = 0.501-0.976; P_IVW_ = 0.036), genus Olsenella (OR_IVW_ = 0.797; 95% CI = 0.667-0.953; P_IVW_ = 0.013), and genus Subdoligranulum (OR_IVW_ = 0.689; 95% CI = 0.482-0.986; P_IVW_ = 0.042) were associated with lower risk of sarcoidosis. No significant heterogeneity and horizontal pleiotropy were found among the five positive results above based on Cochrane’s Q, MR-Egger and MR-PRESSO tests (**Supplementary Table S3**). Nevertheless, the leave-one-out method showed that some SNPs might dominate the positive results of the genus Eubacterium xylanophilum group, genus Olsenella, and genus Subdoligranulum (**Supplementary Figure S11**). What is more, the MR Steiger test of directionality showed the assumption that the family Alcaligenaceae causes a lower risk of sarcoidosis maybe invalid (**Supplementary Table S3**).

#### 3.3.2 Iridocyclitis

This study identified twelve casual relationships between gut microbiota and iridocyclitis (**Figure 4B**). The genetically predicted class Deltaproteobacteria (OR_IVW_ = 1.619; 95% CI = 1.229-2.134; P_IVW_ = 0.001), class Melainabacteria (OR_IVW_ = 1.197; 95% CI = 1.001-1.431; P_IVW_ = 0.049), family Desulfovibrionaceae (OR_IVW_ = 1.752; 95% CI = 1.278-2.402; P_IVW_ = 4.93 × 10^-4^), genus Bilophila (OR_IVW_ = 1.368; 95% CI = 1.078-1.737; P_IVW_ = 0.010), genus Dorea (OR_IVW_ = 1.390; 95% CI = 1.008-1.916; P_IVW_ = 0.044), genus Lachnospiraceae UCG010 (OR_IVW_ = 1.360; 95% CI = 1.038-1.783; P_IVW_ = 0.026), genus Ruminococcaceae UCG013 (OR_IVW_ = 1.314; 95% CI = 1.009-1.712; P_IVW_ = 0.043), and order Desulfovibrionales (OR_IVW_ = 1.590; 95% CI = 1.193-2.121; P_IVW_ = 0.002) were associated with elevated risk of iridocyclitis. In addition, the genetically predicted family Lactobacillaceae (OR_IVW_ = 0.802; 95% CI = 0.660-0.973; P_IVW_ = 0.025), genus Anaerofilum (OR_IVW_ = 0.846; 95% CI = 0.722-0.992; P_IVW_ = 0.039), genus Butyrivibrio (OR_IVW_ = 0.883; 95% CI = 0.792-0.984; P_IVW_ = 0.024), and genus Lactobacillus (OR_IVW_ = 0.817; 95% CI = 0.680-0.983; P_IVW_ = 0.032) were associated with lower risk of iridocyclitis. All the results had no significant heterogeneity and horizontal pleiotropy to be found from the Cochrane’s Q, MR-Egger, and MR-PRESSO tests (**Supplementary Table S3**). The leave-one-out method showed that except for the class Deltaproteobacteria, order Desulfovibrionales, family Desulfovibrionaceae, and genus Bilophila, other positive results might be dominated by one or several SNPs (**Supplementary Figure S15**). It is noteworthy that three of these bacteria reached strong causal associations, namely, the class Deltaproteobacteria, order Desulfovibrionales, and family Desulfovibrionaceae.

#### 3.3.3 Interstitial lung disease

Thirteen casual relationships were identified between gut microbiota and interstitial lung disease (**Figure 4C**). A higher genetically predicted class Deltaproteobacteria (OR_IVW_ = 0.873; 95% CI = 0.769-0.991; P_IVW_ = 0.036), family Lactobacillaceae (OR_IVW_ = 0.918; 95% CI = 0.844-0.999; P_IVW_ = 0.047), genus Butyrivibrio (OR_IVW_ = 0.939; 95% CI = 0.896-0.985; P_IVW_ = 0.009), genus Christensenellaceae R 7 group (OR_IVW_ = 0.852; 95% CI = 0.732-0.991; P_IVW_ = 0.038), genus Eubacterium brachy group (OR_IVW_ = 0.924; 95% CI = 0.865-0.986; P_IVW_ = 0.018), genus Hungatella (OR_IVW_ = 0.910; 95% CI = 0.829-0.999; P_IVW_ = 0.048), genus Lactobacillus (OR_IVW_ = 0.917; 95% CI = 0.846-0.995; P_IVW_ = 0.037), genus Odoribacter (OR_IVW_ = 0.853; 95% CI = 0.741-0.982; P_IVW_ = 0.027), genus Oscillibacter (OR_IVW_ = 0.884; 95% CI = 0.797-0.981; P_IVW_ = 0.020), genus Ruminococcus gnavus group (OR_IVW_ = 0.923; 95% CI = 0.855-0.995; P_IVW_ = 0.037), genus Subdoligranulum (OR_IVW_ = 0.828; 95% CI = 0.735-0.932; P_IVW_ = 0.002), and order Lactobacillales (OR_IVW_ = 0.892; 95% CI = 0.802-0.992; P_IVW_ = 0.034) were associated with lower risk of interstitial lung disease, and the genus Butyricimonas (OR_IVW_ = 1.107; 95% CI = 1.004-1.220; P_IVW_ = 0.041) was associated with increased risk of ILD. All the results had no significant heterogeneity and horizontal pleiotropy to be found from the Cochrane’s Q, MR-Egger, and MR-PRESSO tests (**Supplementary Table S3**). The leave-one-out method revealed that only the genus Butyrivibrio and genus Subdoligranulum met the requirement that no SNP dominate the positive results (**Supplementary Figure S19**).

#### 3.3.4 Atopic dermatitis

Ten taxa were found to have casual relationships with atopic dermatitis (**Figure 4D**). A higher genetically predicted class Clostridia (OR_IVW_ = 0.807; 95% CI = 0.663-0.982; P_IVW_ = 0.033), family Bifidobacteriaceae (OR_IVW_ = 0.807; 95% CI = 0.658-0.989; P_IVW_ = 0.039), genus Bifidobacterium (OR_IVW_ = 0.833; 95% CI = 0.712-0.974; P_IVW_ = 0.022), genus Christensenellaceae R 7 group (OR_IVW_ = 0.742; 95% CI = 0.575-0.958; P_IVW_ = 0.022), genus Dialster (OR_IVW_ = 0.817; 95% CI = 0.684-0.976; P_IVW_ = 0.026), genus Eubacterium brachy group (OR_IVW_ = 0.881; 95% CI = 0.787-0.986; P_IVW_ = 0.027), order Bifidobacteriales (OR_IVW_ = 0.807; 95% CI = 0.658-0.989; P_IVW_ = 0.039), and order Clostridiales (OR_IVW_ = 0.816; 95% CI = 0.673-0.989; P_IVW_ = 0.039) were related to decreased risk of atopic dermatitis. Additionally, a higher genetically predicted genus Lachnospiraceae UCG010 (OR_IVW_ = 1.274; 95% CI = 1.043-1.557; P_IVW_ = 0.018) and genus Ruminococcaceae UCG011 (OR_IVW_ = 1.137; 95% CI = 1.019-1.269; P_IVW_ = 0.022) were correlated with increased risk of atopic dermatitis. All the results had no significant heterogeneity and horizontal pleiotropy to be found from the Cochrane’s Q, MR-Egger, and MR-PRESSO tests (**Supplementary Table S3**). However, the leave-one-out method suggests that all results suffer from the possibility that some SNPs may dominate the results (**Supplementary Figure S23**). Moreover, the class Clostridia and order Clostridiales may invalidly cause decreased risk of atopic dermatitis according to the outcomes from the MR Steiger test of directionality (**Supplementary Table S3**).

#### 3.3.5 Ankylosing spondylitis

A total of nine causal associations were found to exist between gut microbiota and ankylosing spondylitis (**Figure 4E**). A higher genetically predicted class Actinobacteria (OR_IVW_ = 1.695; 95% CI = 1.217-2.360; P_IVW_ = 0.002), family Streptococcaceae (OR_IVW_ = 1.567; 95% CI = 1.021-2.403; P_IVW_ = 0.040), genus Enterorhabdus (OR_IVW_ = 1.736; 95% CI = 1.036-2.908; P_IVW_ = 0.036), and genus Ruminococcaceae NK4A214 group (OR_IVW_ = 1.695; 95% CI = 1.148-2.503; P_IVW_ = 0.008) were associated with enhanced risk of ankylosing spondylitis while the other five gut microbiota were associated with reduced risk of ankylosing spondylitis, namely, the family Lactobacillaceae (OR_IVW_ = 0.671; 95% CI = 0.502-0.898; P_IVW_ = 0.007), family Rikenellaceae (OR_IVW_ = 0.661; 95% CI = 0.466-0.937; P_IVW_ = 0.020), genus Anaerotruncus (OR_IVW_ = 0.671; 95% CI = 0.454-0.991; P_IVW_ = 0.045), genus Howardella (OR_IVW_ = 0.802; 95% CI = 0.650-0.991; P_IVW_ = 0.041), and genus Oscillibacter (OR_IVW_ = 0.403; 95% CI= 0.168-0.966; P_IVW_ = 0.042). None of the results suggested the presence of significant heterogeneity based on the Cochrane’s Q test (p > 0.05) (**Supplementary Table S3**). There was no heterogeneity or horizontal pleiotropy in any of the eight bacteria, except for the genus Oscillibacter, which had inconsistent MR-Egger and MR-PRESSO test results (**Supplementary Table S3**). Additionally, the genus Oscillibacter and genus Anaerotruncus may suffer from an invalid causal relationship with the lower risk of ankylosing spondylitis based on the result of the MR Steiger test of directionality. Furthermore, only four gut microbiota, namely, the class Actinobacteria, family Lactobacillaceae, family Rikenellaceae, and genus Ruminococcaceae NK4A214 group, met the requirement that no single SNP dominate the positive results. It is noteworthy that the association of the class Actinobacteria with ankylosing spondylitis is significant (**Supplementary Figure S27**).

#### 3.3.6 Psoriatic arthropathies

The genetically predicted genus Adlercreutzia (OR_IVW_ = 1.458; 95% CI = 1.014-2.095; P_IVW_ = 0.042), genus Anaerotruncus (OR_IVW_ = 1.486; 95% CI = 1.007-2.195; P_IVW_ = 0.046), and genus Coprococcus1 (OR_IVW_ = 1.496; 95% CI = 1.014-2.208; P_IVW_ = 0.042) were associated with increased risk of psoriatic arthropathies while the genetically predicted family Rikenellaceae (OR_IVW_ = 0.618; 95% CI = 0.436-0.876; P_IVW_ = 0.007) and genus Odoribacter (OR_IVW_ = 0.488; 95% CI = 0.295-0.806; P_IVW_ = 0.005) were associated with decreased risk of psoriatic arthropathies (**Figure 4F**). No significant heterogeneity and horizontal pleiotropy were found among the five positive results above based on the Cochrane’s Q, MR-Egger, and MR-PRESSO tests (**Supplementary Table S3**). The MR Steiger test of directionality discovered a false direction in the causal relationship between the genus Anaerotruncus and a lower risk of psoriatic arthropathies. In addition, the leave-one-out method identified that no single SNP dominated the statistically significant results of the family Rikenellaceae and genus Odoribacter while the remaining three texa did (**Supplementary Figure S31**). Notably, genetically predicted IBD was also causally associated with the genus Odoribacter. For this reason, we further calculated the mediating role of the genus Odoribacter in IBD and psoriatic arthropathies:  βi = β1*β2/β = −0.106 (β1:IBD → genus Odoribacte; β2: genus Odoribacte → psoriatic arthropathies; β: IBD → psoriatic arthropathies).

#### 3.3.7 Primary sclerosing cholangitis

We identified eight causal correlations between gut microbiota and primary sclerosing cholangitis (**Figure 4G**). A higher genetically predicted class Mollicutes (OR_IVW_ = 1.664; 95% CI = 1.061-2.609; P_IVW_ = 0.027), family Oxalobacteraceae (OR_IVW_ = 1.435; 95% CI = 1.077-1.913; P_IVW_ = 0.014), and phylum Tenericutes (OR_IVW_ = 1.664; 95% CI = 1.061-2.609; P_IVW_ = 0.027) were related to enhanced risk of PBC while the family Actinomycetaceae (OR_IVW_ = 0.595; 95% CI = 0.361-0.982; P_IVW_ = 0.042), family Clostridiales vadin BB60 group (OR_IVW_ = 0.729; 95% CI = 0.544-0.978; P_IVW_ = 0.035), genus Actinomyces (OR_IVW_ = 0.618; 95% CI = 0.423-0.901; P_IVW_ = 0.012), genus Alloprevotella (OR_IVW_ = 0.685; 95% CI = 0.5-0.938; P_IVW_ = 0.018), and order Actinomycetales (OR_IVW_ = 0.594; 95% CI = 0.36-0.981; P_IVW_ = 0.042) were related to reduced risk of PSC. There was no significant heterogeneity and horizontal pleiotropy from the results of the Cochrane’s Q, MR-Egger, and MR-PRESSO tests (**Supplementary Table S3**). According to the leave-one-out method, the presence of some SNPs in all results may dominate statistically significant results (**Supplementary Figure S35**).

#### 3.3.8 Primary biliary cholangitis

Five casual relationships were found according to the IVW method (**Figure 4H**). A higher genetically predicted genus Lachnospiraceae FCS020 group (OR_IVW_ = 1.829; 95% CI = 1.053-3.178; P_IVW_ = 0.032) and genus Lachnospiraceae UCG004 (OR_IVW_ = 1.831; 95% CI = 1.098-3.054; P_IVW_ = 0.02) were associated with higher risk of PBC while the family Desulfovibrionaceae (OR_IVW_ = 0.542; 95% CI = 0.297-0.989; P_IVW_ = 0.046), order Desulfovibrionales (OR_IVW_ = 0.542; 95% CI = 0.297-0.989; P_IVW_ = 0.046), and genus Prevotella 9 (OR_IVW_ = 0.532; 95% CI = 0.3-0.944; P_IVW_ = 0.031) were associated with lower risk of PBC. All the results had no significant heterogeneity and horizontal pleiotropy to be found from the Cochrane’s Q, MR-Egger, and MR-PRESSO tests (**Supplementary Table S3**). However, the leave-one-out method indicated that there are SNPs that might dominate all of these five positive results (**Supplementary Figure S39**).

## 4 Discussion

Based on multiple GWAS datasets, this study explored the causal associations between inflammatory bowel disease and its associated 20 extraintestinal manifestations using a Mendelian randomization study and found that genetically predicted IBD was causally associated with eight extraintestinal manifestations: sarcoidosis, iridocyclitis, ankylosing spondylitis, atopic dermatitis, interstitial lung disease, psoriatic arthritis, primary sclerosing cholangitis, and primary biliary cholangitis. As IBD leads to an increased risk of developing these eight extraintestinal manifestations, we further used large-scale genetic data on gut microbiota to explore its role in these relationships.

Early-onset sarcoidosis (EOS) has been shown to be associated with mutations in the NOD2/CARD15 gene, which has also been identified as one of the genes associated with susceptibility to CD (23, 24). 7E_12_H_12_ is a murine monoclonal antibody against a 40,000 relative-molecular-mass colonic protein (P40) on colonic epithelial cells. Cross-reactive peptides reactive with 7E_12_H_12_ MAb were found in the non-pigmented epithelial cells of the ciliary processes, the bile duct, the hepatic ducts, and the skin (25, 26). This corroborates our results that IBD is causally associated with iridocyclitis and PSC. Type 3 innate lymphoid cells (ILC3) differentiate in the intestine and migrate to extraintestinal sites, especially recirculating between the intestine and bone marrow (BM), and are involved in the development of ankylosing spondylitis (AS) through the production of IL-17 and IL-22, which may be responsible for the induction of inflammation (27). Citrullination and neutrophil extracellular traps (NETs) are involved in the process of inflammation and fibrosis in ILD, which are reported in the etiology of the inflammatory bowel diseases (28–30). Mitochondrial autophagy appears to be involved in the pathogenesis of both IBD and PBC (31). PDC-E2, the hallmark antigen of PBC, induces anti-mitochondrial antibodies (AMA) that cross-react with Novosphingobium aromaticivorans, which are conditioned by the disruption of the intestinal barrier and the increase in permeability that occurs in inflammatory bowel diseases (32). In addition, an increase in Th17 cells, the main IL17-secreting cells, is observed in atopic dermatitis, psoriatic arthritis, and the six extraintestinal manifestations mentioned above and is thought to be involved in the pathogenesis of these diseases, which likewise play an important role in the pathogenesis of IBD (33, 34). For the 12 excluded extraintestinal manifestations, the MR results were inconsistent with those of the observational studies, which we considered to be due to possible confounders and reverse causal associations in the observational studies. Commonly used medications for the treatment of patients with IBD have been found to be associated with an increased risk of multiple extraintestinal manifestations. IBD and increased risk of rheumatoid arthritis, psoriasis, and asthma may be associated with tumor necrosis factor inhibitors (35, 36). Mesalazine and cyclosporine are thought to carry a risk of inducing acute pancreatitis, and cyclosporine is also thought to cause an increased incidence of cholelithiasis (37, 38). Insulin resistance and type 2 diabetes, on the other hand, are side effects of glucocorticoid (39). Several studies have shown significantly higher rates of obesity in patients with IBD, which is also thought to cause an increased risk of developing many autoimmune diseases, including type 1 diabetes and multiple sclerosis, and vascular diseases (40–43). In addition, a pro-inflammatory diet characterized by high sugar and fat is a risk factor for IBD, which may be a shared risk factor with fatigue, which is reduced in IBD patients with improved diet quality (44, 45).

A total of 11 causal associations between IBD and gut microbiota reached nominal significance, of which five were stable causal associations, specifically the genus Erysipelatoclostridium, genus Holdemanella, genus Odoribacter, genus Ruminococcus 2, and genus Ruminococcus gauvreauii group. Genetically predicted IBD is associated with a higher abundance of the genus Erysipelatoclostridium and a lower abundance of the genus Holdemanella, which is consistent with the overall change in the increased relative abundance of beneficial bacteria and decreased relative abundance of harmful bacteria in the gut of IBD patients (21). The genus Erysipelatoclostridium is a potentially harmful bacterium, while the genus Holdemanella is a beneficial bacterium, inhibiting inflammation by producing long-chain fatty acids (46, 47). Genetically predicted IBD is associated with lower abundance of genus Ruminococcus2 and genus Ruminococcus gauvreauii group, which is contrary to the results of existing studies (48). We hypothesize that the genus Ruminococcus may be involved in promoting intestinal inflammation, which may suggest that this bacterium is a potential biomarker for IBD. The abundance of the genus Odoribacter was found to decrease in Crohn’ s disease and pancolitis which is also inconsistent with the results of existing studies (49). The genus Odoribacter is a gram-negative and SCFA-producing bacterium, including three species within the genus: O. denticanis, O. laneus, and O. splanchnicus. The reason for its reduced abundance may suggest that its traits are initiators of IBD, and that the host can produce specific antibodies to reduce the abundance of these bacterial traits.

A total of 67 causal associations were identified as nominally significant in intestinal flora and eight extraintestinal manifestations, i.e., 5 in sarcoidosis, 12 in iridocyclitis, 13 in interstitial lung disease, 10 in atopic dermatitis, 9 in ankylosing spondylitis, 5 in psoriatic arthritis, 5 in PSC, and 8 in PBC. Thirteen of these associations are stable, specifically the family Victivallaceae and sarcoidosis; the class Deltaproteobacteria, order Desulfovibrionales, family Desulfovibrionaceae, genus Bilophila, and iridocyclitis; the genus Butyrivibrio, genus Subdoligranulum, and interstitial lung disease; the class Actinobacteria; the family Lactobacillaceae, family Rikenellaceae, genus Ruminococcaceae NK4A214 group, and ankylosing spondylitisand; and the family Rikenellaceae, genus Odoribacter, and psoriatic arthritis. Notably, the genus Odoribacter was causally associated with both IBD and psoriatic arthritis, i.e., genetically predicted inflammatory bowel disease resulted in increased abundance of genus Odoribacter whereas increased (reduced) abundance of genus Odoribacter caused a reduced (increased) risk of psoriatic arthritis onset. However, IBD is associated with an increased risk of psoriatic arthritis, so the genus Odoribacter does not play a mediating role in this association. What is more, the association of the class Deltaproteobacteria, order Desulfovibrionales, family Desulfovibrionaceae, and iridocyclitis, and the association of the class Actinobacteria and ankylosing spondylitis reached significant correlation. Enrichment of the class Actinobacteria was observed in patients with ankylosing spondylitis, which is consistent with our MR results (50). The class Actinobacteria has proteasomes and can degrade target proteins through a process called pupylation, which utilizes Pup, an ubiquitin-like protein, to bind the protein substrate, which is then degraded by the proteasome (51). IκBs are inhibitory proteins of NF-κB that block nuclear uptake by binding to NF-κB and masking its nuclear localization signal and they are regulated by ubiquitination (52). Considering that increased abundance of the class Actinobacteria causes an increased risk of developing ankylosing spondylitis, then the class Actinobacteria might be involved in ubiquitinated degradation of IκBs, which in turn activates the NF-κB pathway (53). Although there are no studies on the association of the class Deltaproteobacteria, order Desulfovibrionales, family Desulfovibrionaceae, and genus Bilophila with iridocyclitis, there are case reports of iridocyclitis due to endogenous infections (54). Therefore, we hypothesized that it is possible for these four bacteria to cause iridocyclitis by bacterial translocation, especially the three strongly related groups. Actually, there are no studies on the relationship between the rest of bacterial taxa and corresponding extraintestinal manifestations. Thus, our study may provide a new perspective for mechanistic research on these extraintestinal manifestations.

Our study also has limitations. First, the dataset used was predominantly European populations, exclusively or mostly European, which is not conducive to the extension of the results. Second, when obtaining SNPs from the colony dataset, we set the P-value to 1 × 10^-5^ considering that too few SNPs were obtained by setting the locus-wide significance level to 5 × 10^-8^. Although all SNP F-values were >10, weak instrumental variable bias is still something to be aware of. Third, this study was unable to determine whether overlapping participants were enrolled in the exposure and outcome GWAS used in the two-sample MR analyses. Fourth, only 20 extraintestinal manifestations were included in our study, and there are many other IBD-related extraintestinal manifestations that were not included in the study. Fifth, only the mediating role of intestinal bacteria was analyzed in this paper, while there are fungi, viruses, and other microorganisms in the gut that were not included in the analysis. Sixth, the study consists of multiple datasets, and the standards applied to sample collection, processing, and sequencing may affect comparisons. Seventh, further clinical validation with experimental studies to confirm causality and the long-term impact of the examination on gut microbiota interventions are required.

## 5 Conclusion

By using the methodology of a Mendelian randomization study, we identified eight extraintestinal manifestations for which an elevated risk of morbidity was associated with the development of inflammatory bowel disease, and we identified a stable nominal causal association between IBD and five flora. Through further Mendelian randomization of the causal associations between intestinal flora and extraintestinal manifestations, a total of 67 nominal causal associations were identified, of which 13 associations were stable, and notably 4 associations were strongly correlated. Although we did not find any gut microbiota playing a mediating role in the causal association between IBD and several of its associated extraintestinal manifestations, these associations may provide useful biomarkers and potential targets for pathogenesis and treatment.

## Data availability statement

The original contributions presented in the study are included in the article/**Supplementary Material**. Further inquiries can be directed to the corresponding authors.

## Ethics statement

Publicly available GWAS data were used for this study. Relevant informed consent and ethical approval were obtained for the original study. Therefore, additional ethical approval was not required in the present study.

## Author contributions

WL: Data curation, Software, Visualization, Writing – original draft, Conceptualization, Formal analysis, Investigation, Methodology. QD: Investigation, Software, Writing – original draft. JC: Investigation, Methodology, Software, Writing – original draft. HT: Funding acquisition, Methodology, Project administration, Supervision, Writing – review & editing, Conceptualization, Formal analysis. LP: Formal Analysis, Funding acquisition, Project administration, Supervision, Writing – review & editing.
