# Supplementary material for: Gut microbiota does not play a mediating role in the causal association between inflammatory bowel disease and several its associated extraintestinal manifestations: a Mendelian randomization study
Source: Front Immunol. 2024 Jan 15;14:1296889. doi: 10.3389/fimmu.2023.1296889 (PMC10822939; doi:10.3389/fimmu.2023.1296889)
Supplement: Supplementary file 1 [file DataSheet_1.zip › Supplementary Data Sheet 1.docx]

[Supplement Table S1 4](#_Toc24049)

[Supplement Table S2 4](#_Toc3131)

[Supplement Table S3. 4](#_Toc16976)

[Supplement Table S4. 4](#_Toc24291)

[Supplement Table S5. 4](#_Toc1958)

[Supplement Table S6. 4](#_Toc8479)

[Supplement Figure S1 5](#_Toc11230)

[Supplement Figure S2 6](#_Toc3793)

[Supplement Figure S3 7](#_Toc598)

[Supplement Figure S4 8](#_Toc8414)

[Supplement Figure S5 9](#_Toc12145)

[Supplement Figure S7 12](#_Toc1252)

[Supplement Figure S8 13](#_Toc505)

[Supplement Figure S9 15](#_Toc20383)

[Supplement Figure S10 16](#_Toc8297)

[Supplement Figure S11 17](#_Toc10126)

[Supplement Figure S12 18](#_Toc29063)

[Supplement Figure S13 18](#_Toc20879)

[Supplement Figure S14 19](#_Toc8971)

[Supplement Figure S16 22](#_Toc6030)

[Supplement Figure S18 25](#_Toc1558)

[Supplement Figure S19 27](#_Toc377)

[Supplement Figure S20 28](#_Toc30921)

[Supplement Figure S21 30](#_Toc23326)

[Supplement Figure S22 31](#_Toc3394)

[Supplement Figure S23 33](#_Toc5528)

[Supplement Figure S24 34](#_Toc25027)

[Supplement Figure S25 36](#_Toc18798)

[Supplement Figure S26 37](#_Toc31442)

[Supplement Figure S27 38](#_Toc1705)

[Supplement Figure S28 39](#_Toc14223)

[Supplement Figure S29 40](#_Toc19026)

[Supplement Figure S30 41](#_Toc31129)

[Supplement Figure S31 42](#_Toc27971)

[Supplement Figure S32 43](#_Toc9525)

[Supplement Figure S33 43](#_Toc15927)

[Supplement Figure S34 44](#_Toc921)

[Supplement Figure S35 45](#_Toc18795)

[Supplement Figure S36 46](#_Toc8318)

[Supplement Figure S37 47](#_Toc8796)

[Supplement Figure S38 48](#_Toc17775)

[Supplement Figure S39 49](#_Toc26724)

[Supplement Figure S40 50](#_Toc2159)

[Supplement Figure S41 51](#_Toc17727)

Supplement Table S1. Datasets used and their citations (the relevant table is available in the Supplement Tables.xlsx).

Supplement Table S2. Methods used and their citations (the relevant table is available in the Supplement Tables.xlsx).

Supplement Table S3. Sensitivity analysis of IBD on IBD-related EIMs (the relevant table is available in the Supplement Tables.xlsx).

**Supplement Table S4.** Sensitivity analysis of IBD on gut microbiota (the relevant table is available in the Supplement Tables.xlsx).

Supplement Table S5. Sensitivity analysis of gut microbiota on IBD-related EIMs (the relevant table is available in the Supplement Tables.xlsx).

Supplement Table S6. The GWAS ID of gut microbiota abundance (the relevant table is available in the Supplement Tables.xlsx).

**
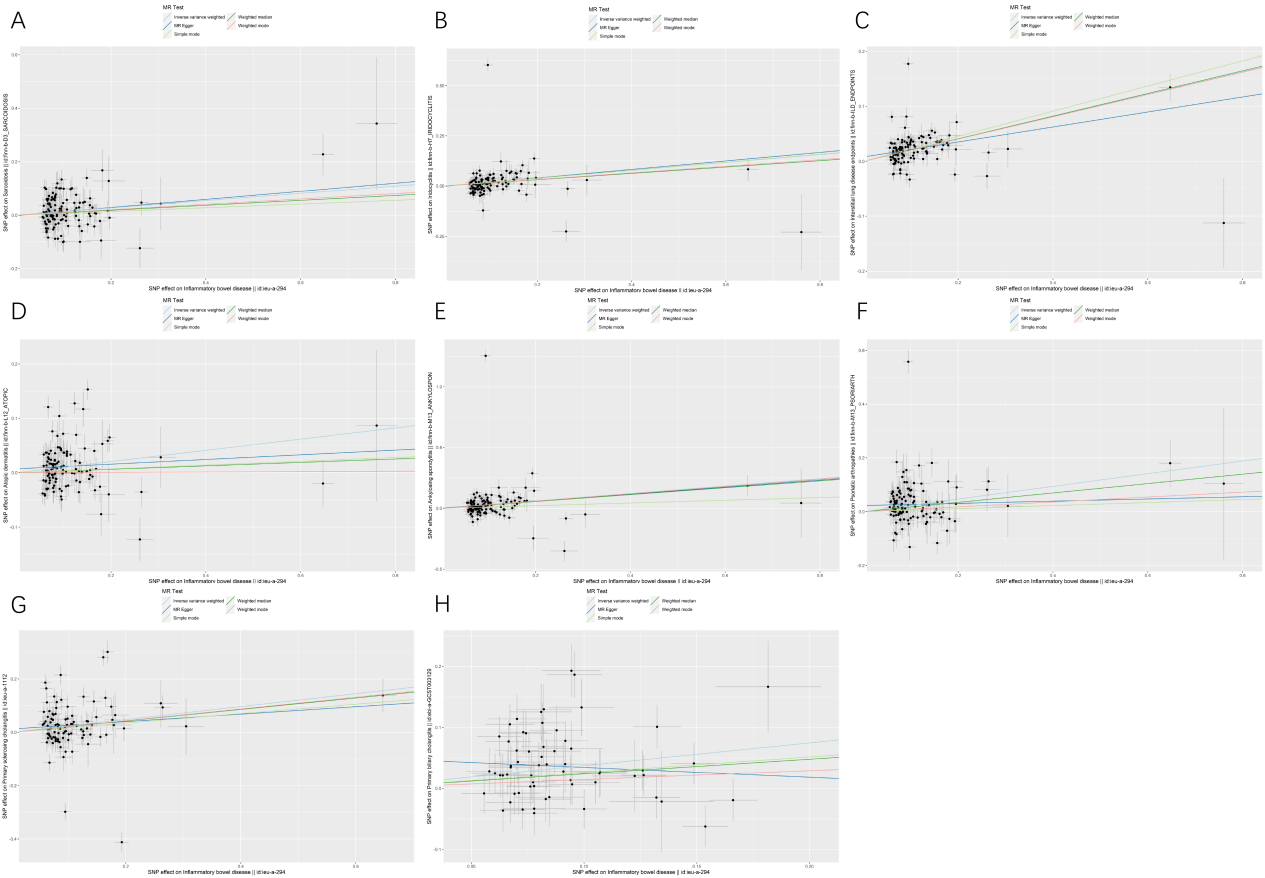
**

Supplement Figure S1

Scatter plots of estimates from genetically predicted IBD on twenty IBD-related Extraintestinal Manifestations. (A)Sarcoidosis; (B)Iridocyclitis; (C)Interstitial lung disease; (D)Atopic dermatitis; (E)Ankylosing spondylitis; (F)Psoriatic arthropathies; (G)Primary sclerosing cholangitis; (H)Primary biliary cholangitis.

**
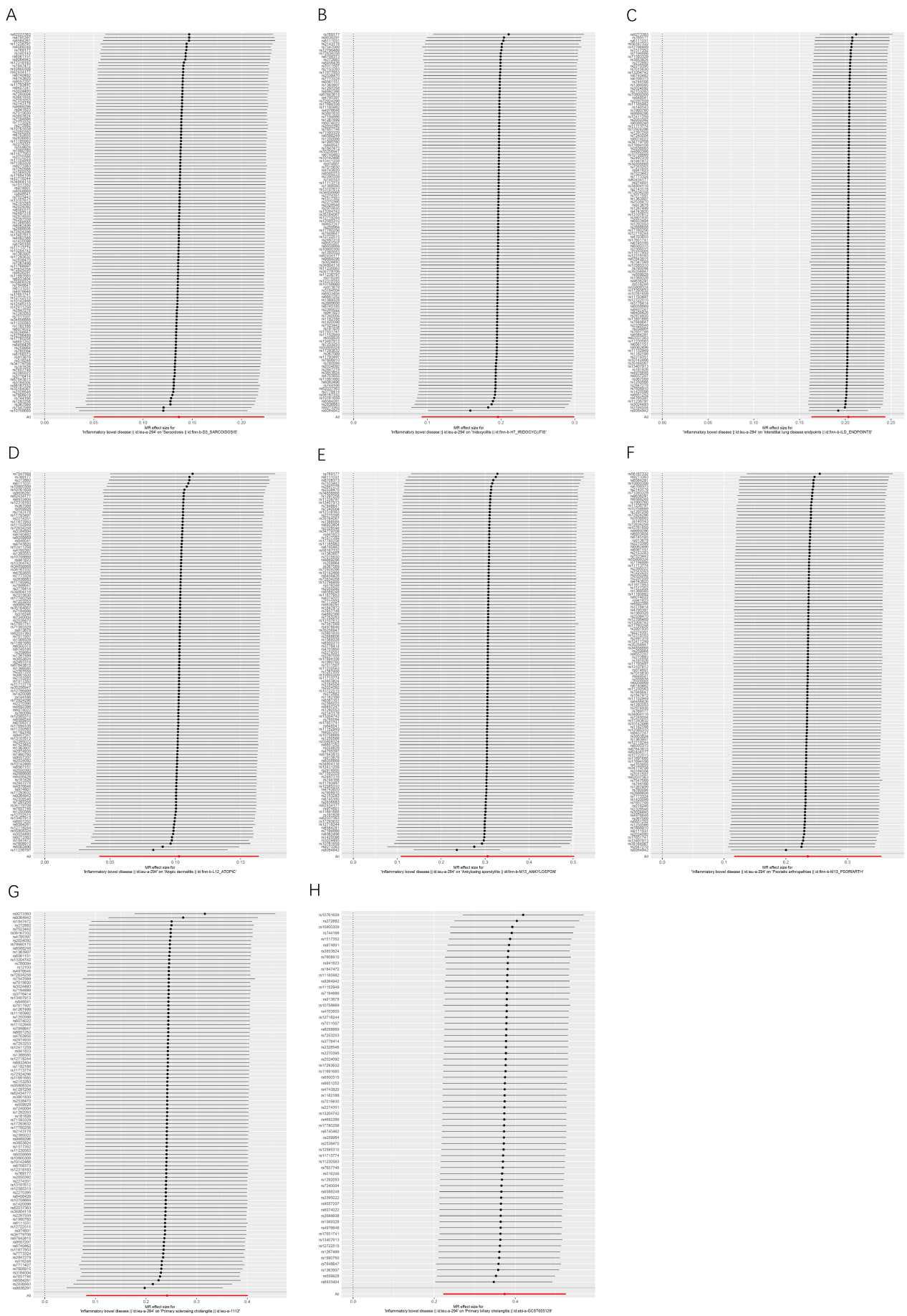
**

Supplement Figure S2

Leave-one-out plots plots of estimates from genetically predicted IBD on twenty IBD-related Extraintestinal Manifestations. (A)Sarcoidosis; (B)Iridocyclitis; (C)Interstitial lung disease; (D)Atopic dermatitis; (E)Ankylosing spondylitis; (F)Psoriatic arthropathies; (G)Primary sclerosing cholangitis; (H)Primary biliary cholangitis.

**
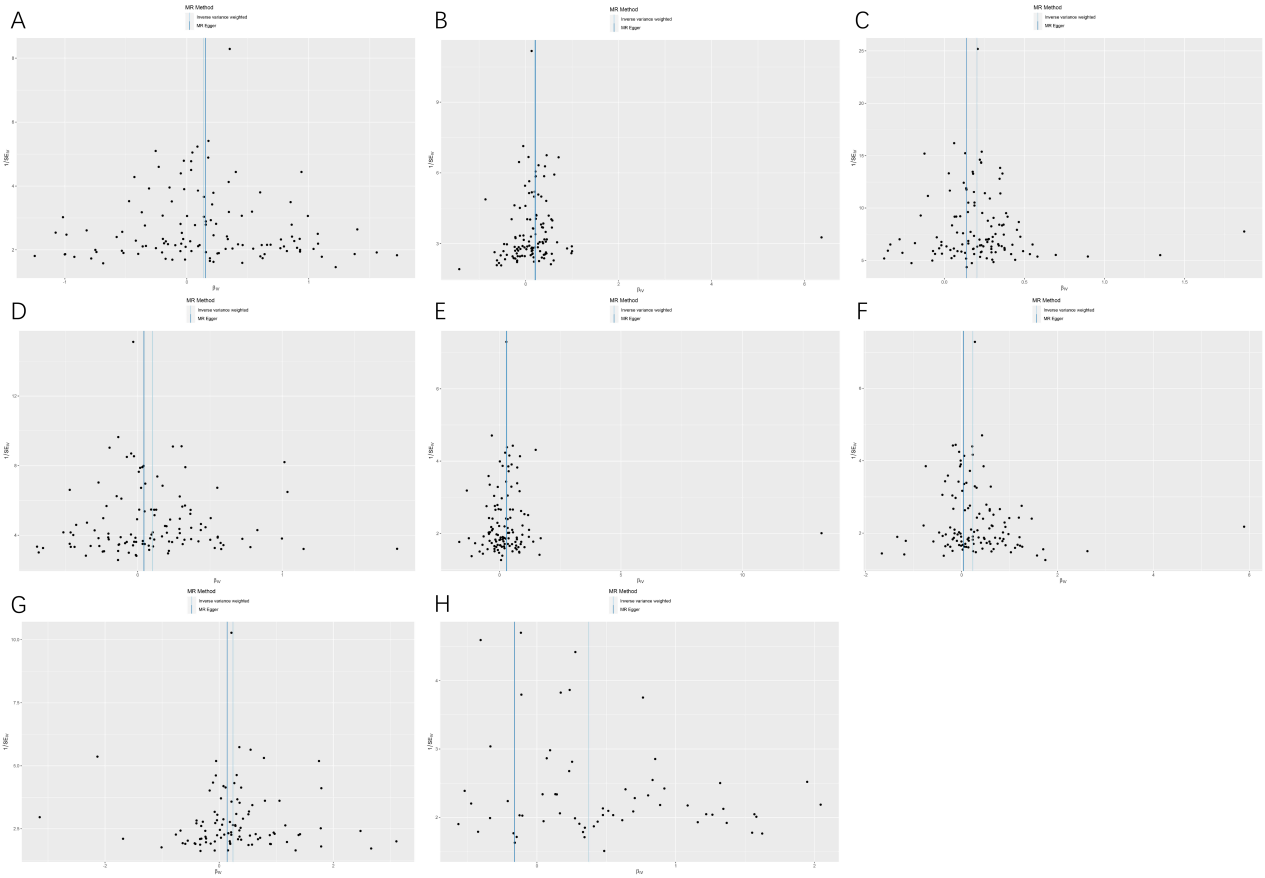
**

Supplement Figure S3

Funnel plots of estimates from genetically predicted IBD on twenty IBD-related Extraintestinal Manifestations. (A)Sarcoidosis; (B)Iridocyclitis; (C)Interstitial lung disease; (D)Atopic dermatitis; (E)Ankylosing spondylitis; (F)Psoriatic arthropathies; (G)Primary sclerosing cholangitis; (H)Primary biliary cholangitis.

**
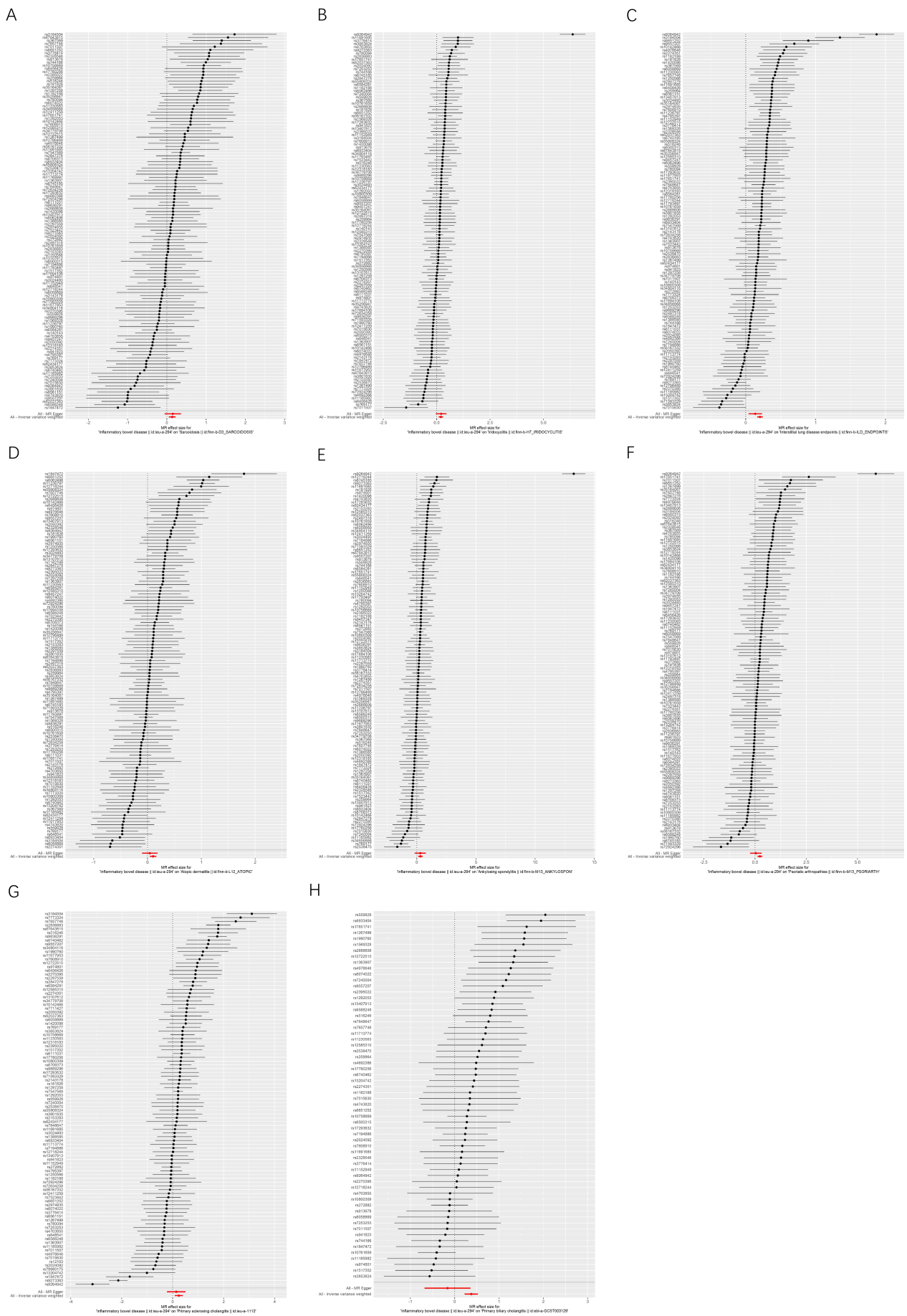
**

Supplement Figure S4

Single plots of estimates from genetically predicted IBD on twenty IBD-related Extraintestinal Manifestations. (A)Sarcoidosis; (B)Iridocyclitis; (C)Interstitial lung disease; (D)Atopic dermatitis; (E)Ankylosing spondylitis; (F)Psoriatic arthropathies; (G)Primary sclerosing cholangitis; (H)Primary biliary cholangitis.

**
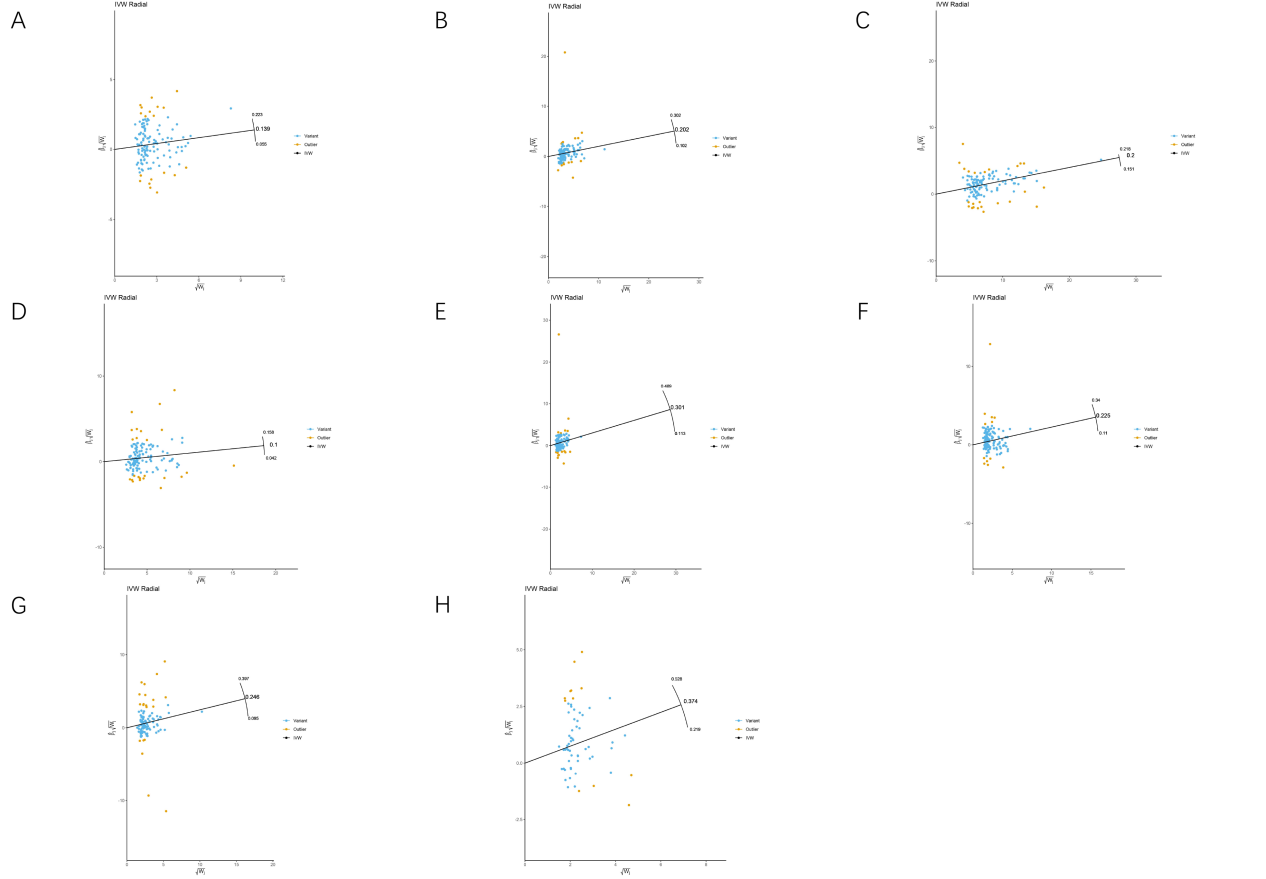
**

Supplement Figure S5

Radial plots of estimates from genetically predicted IBD on twenty IBD-related Extraintestinal Manifestations. (A)Sarcoidosis; (B)Iridocyclitis; (C)Interstitial lung disease; (D)Atopic dermatitis; (E)Ankylosing spondylitis; (F)Psoriatic arthropathies; (G)Primary sclerosing cholangitis; (H)Primary biliary cholangitis.

**
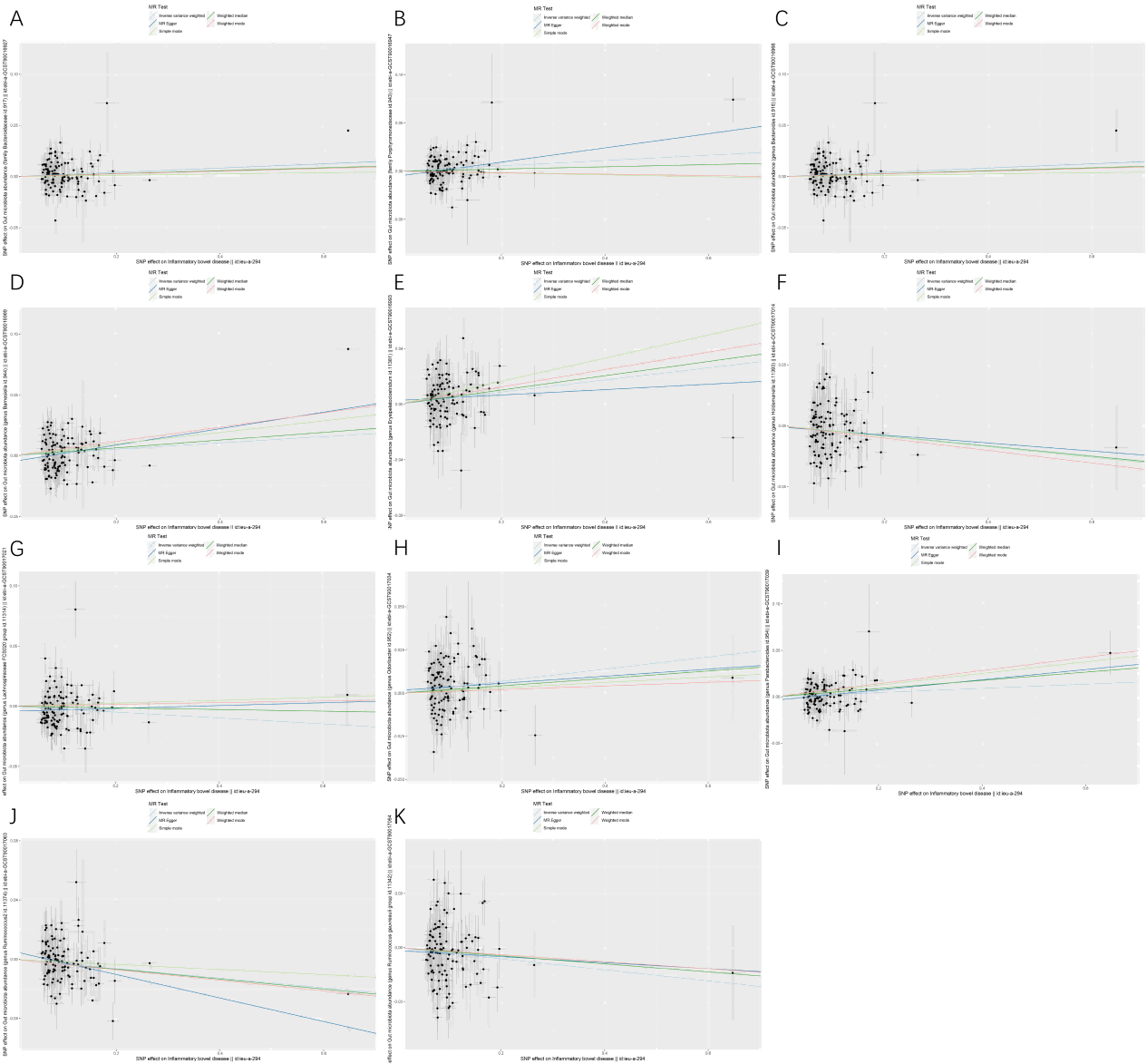
**

**Supplement Figure S6**

Scatter plots significant and nominal significant estimates from genetically predicted IBD on gut microbiota. (A)family Bacteroidaceae; (B)family Porphyromonadaceae; (C)genus Bacteroides; (D)genus Barnesiella; (E)genus Erysipelatoclostridium; (F)genus Holdemanella; (G)genus Lachnospiraceae FCS020 group; (H)genus Odoribacter; (I)genus Parabacteroides; (J)genus Ruminococcus2; (K)genus Ruminococcus gauvreauii group.

**
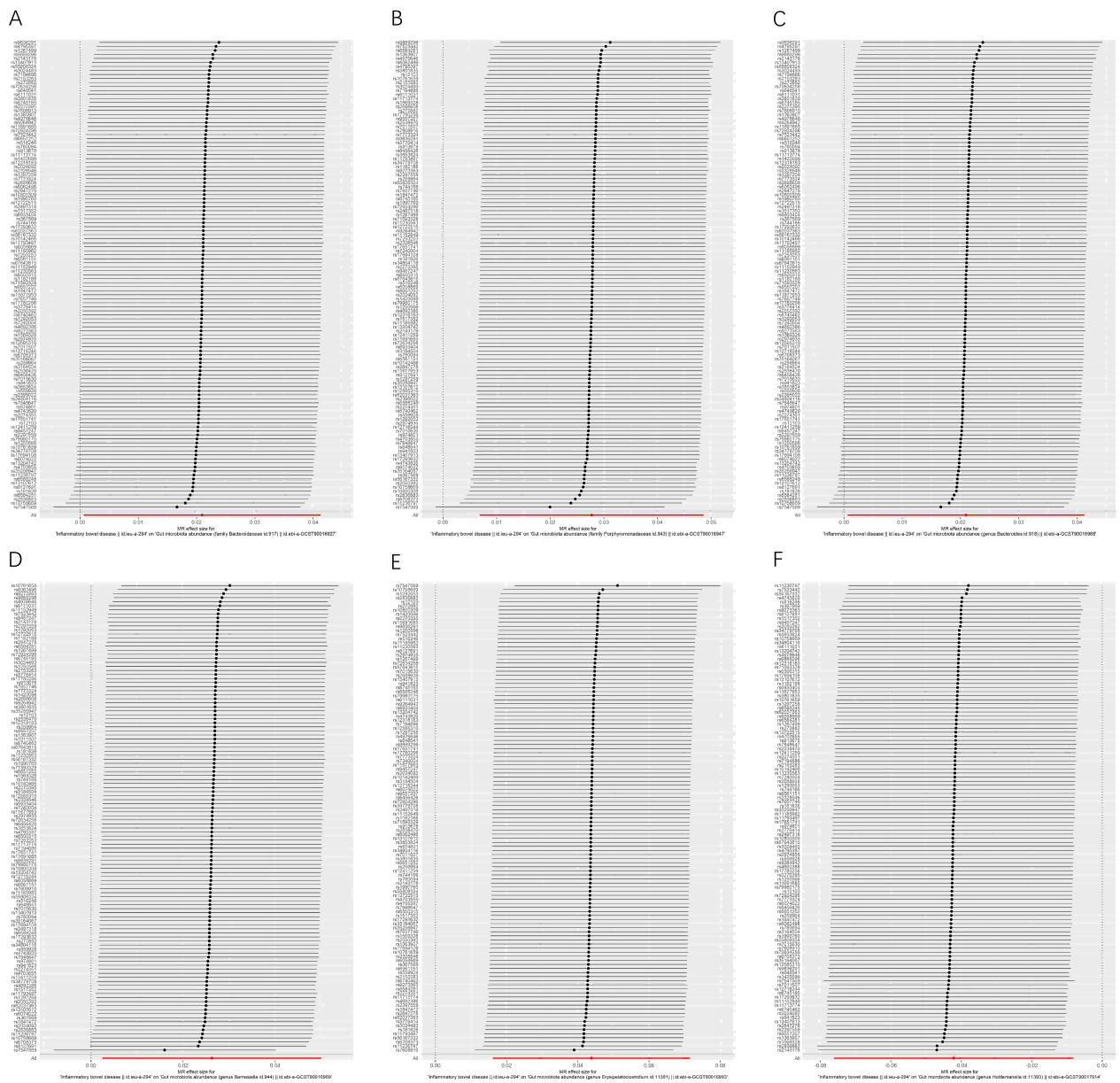
**

**
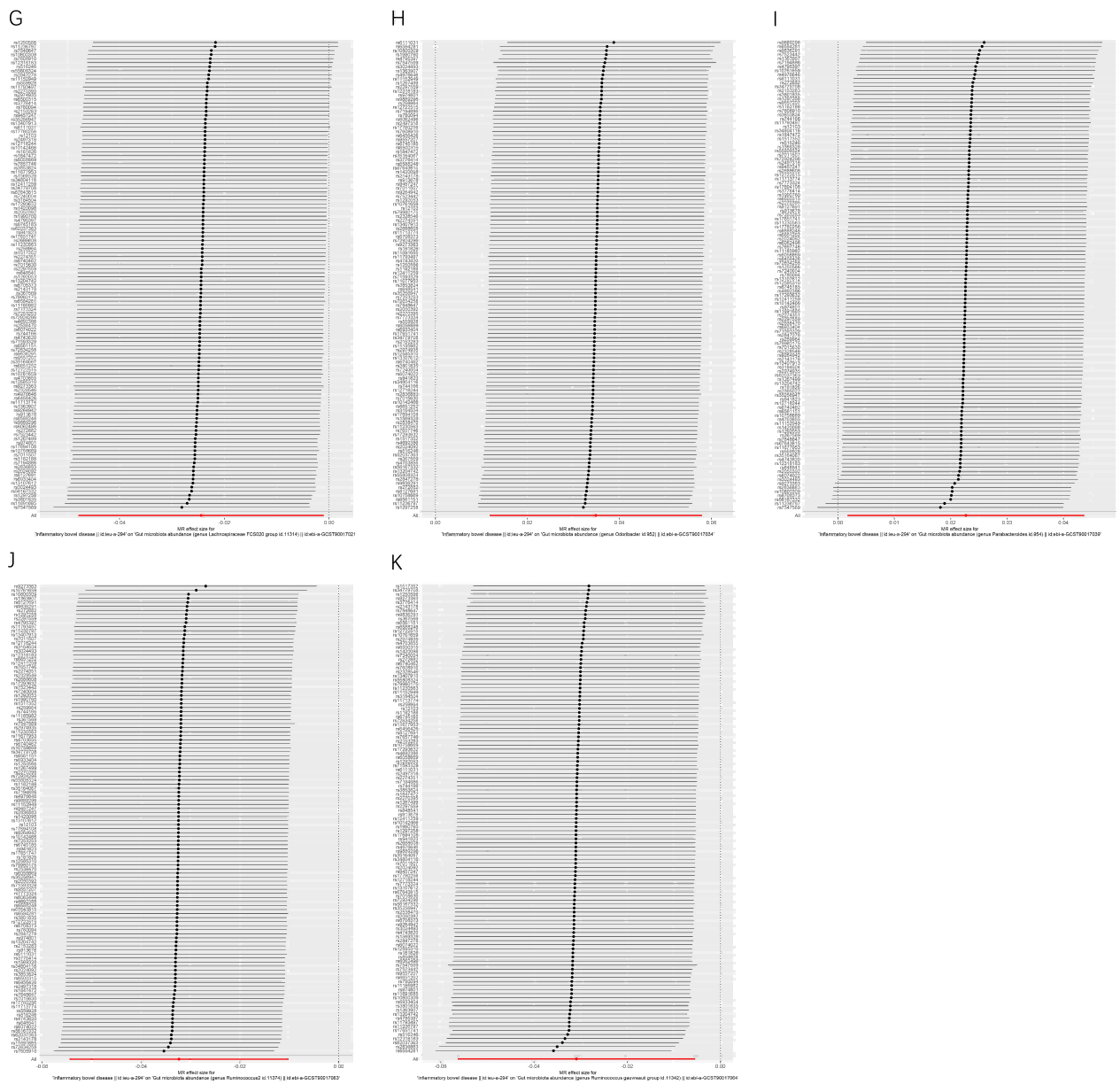
**

Supplement Figure S7

Leave-one-out plots significant and nominal significant estimates from genetically predicted IBD on gut microbiota. (A)family Bacteroidaceae; (B)family Porphyromonadaceae; (C)genus Bacteroides; (D)genus Barnesiella; (E)genus Erysipelatoclostridium; (F)genus Holdemanella; (G)genus Lachnospiraceae FCS020 group; (H)genus Odoribacter; (I)genus Parabacteroides; (J)genus Ruminococcus2; (K)genus Ruminococcus gauvreauii group.

**
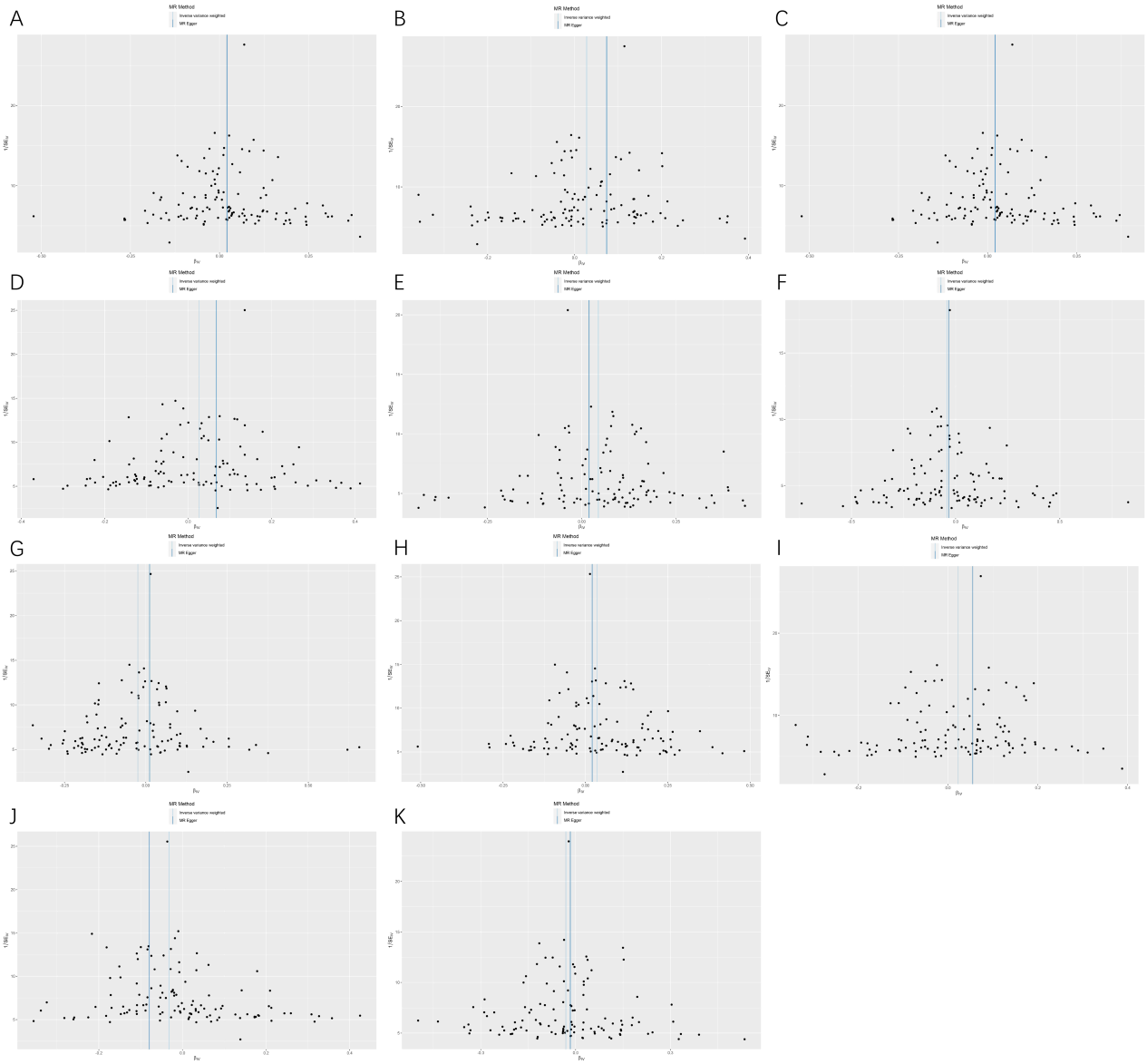
**

Supplement Figure S8

Funnel plots significant and nominal significant estimates from genetically predicted IBD on gut microbiota. (A)family Bacteroidaceae; (B)family Porphyromonadaceae; (C)genus Bacteroides; (D)genus Barnesiella; (E)genus Erysipelatoclostridium; (F)genus Holdemanella; (G)genus Lachnospiraceae FCS020 group; (H)genus Odoribacter; (I)genus Parabacteroides; (J)genus Ruminococcus2; (K)genus Ruminococcus gauvreauii group.

**
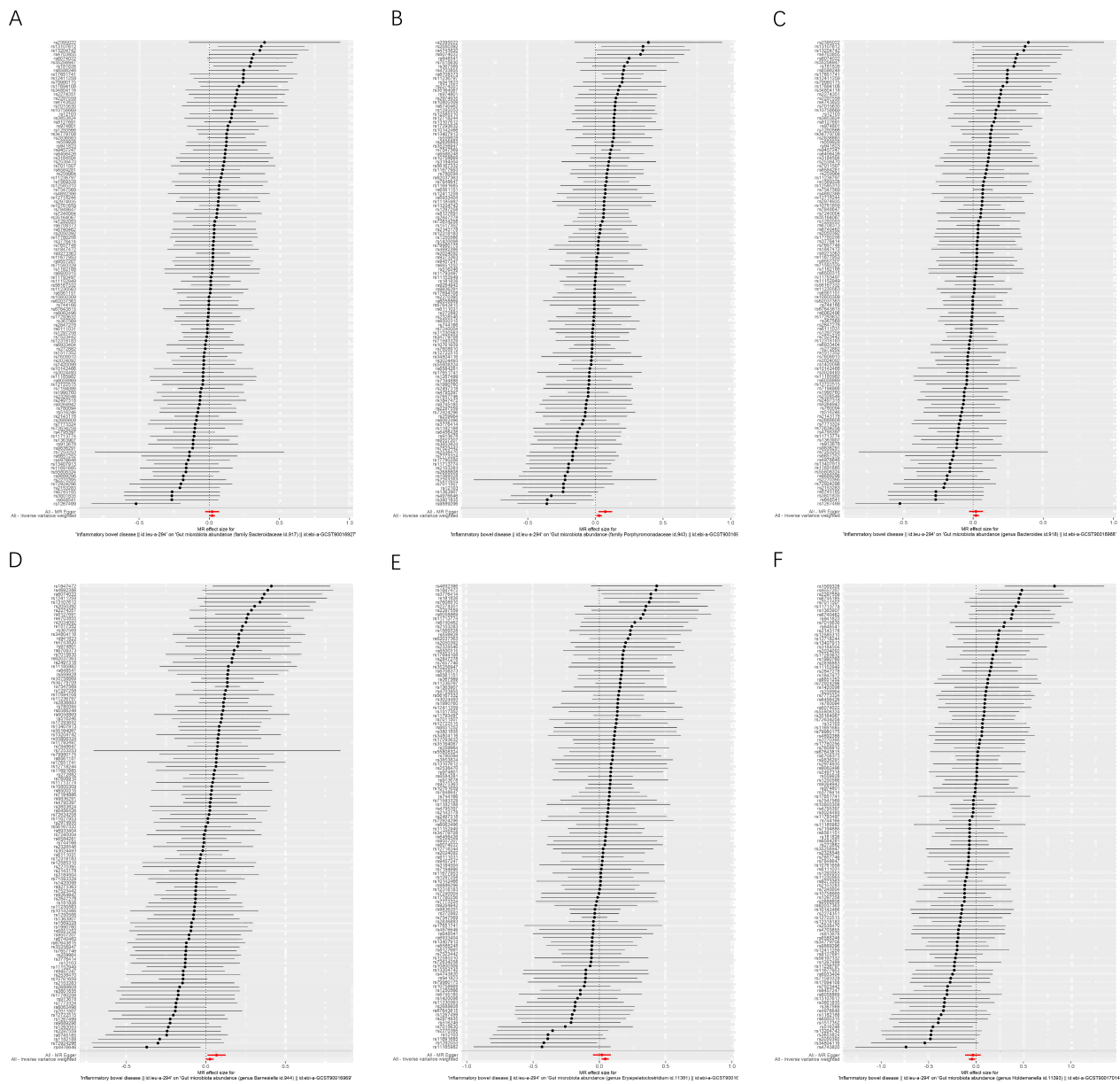
**

**
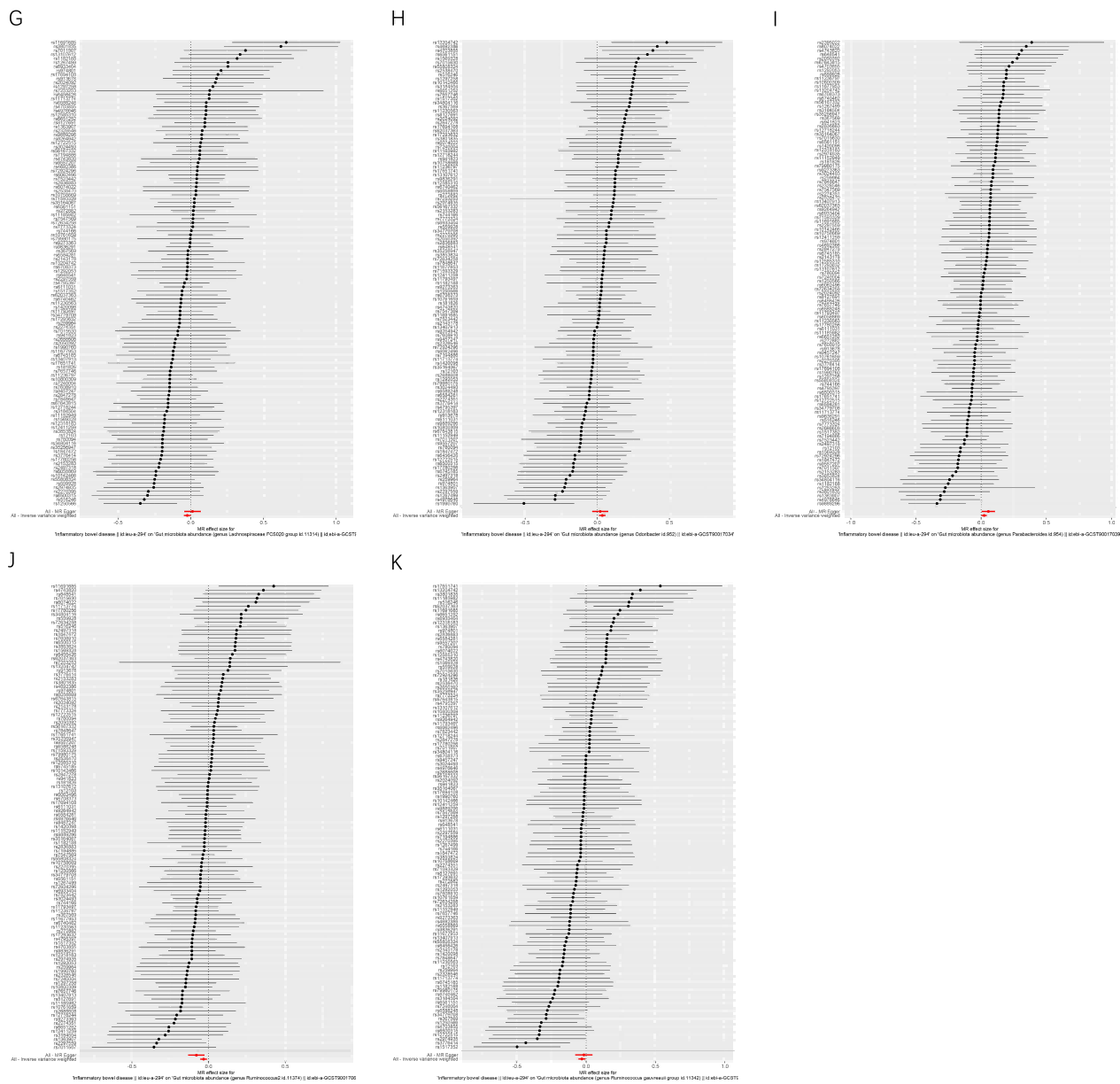
**

Supplement Figure S9

Single plots significant and nominal significant estimates from genetically predicted IBD on gut microbiota. (A)family Bacteroidaceae; (B)family Porphyromonadaceae; (C)genus Bacteroides; (D)genus Barnesiella; (E)genus Erysipelatoclostridium; (F)genus Holdemanella; (G)genus Lachnospiraceae FCS020 group; (H)genus Odoribacter; (I)genus Parabacteroides; (J)genus Ruminococcus2; (K)genus Ruminococcus gauvreauii group.

**
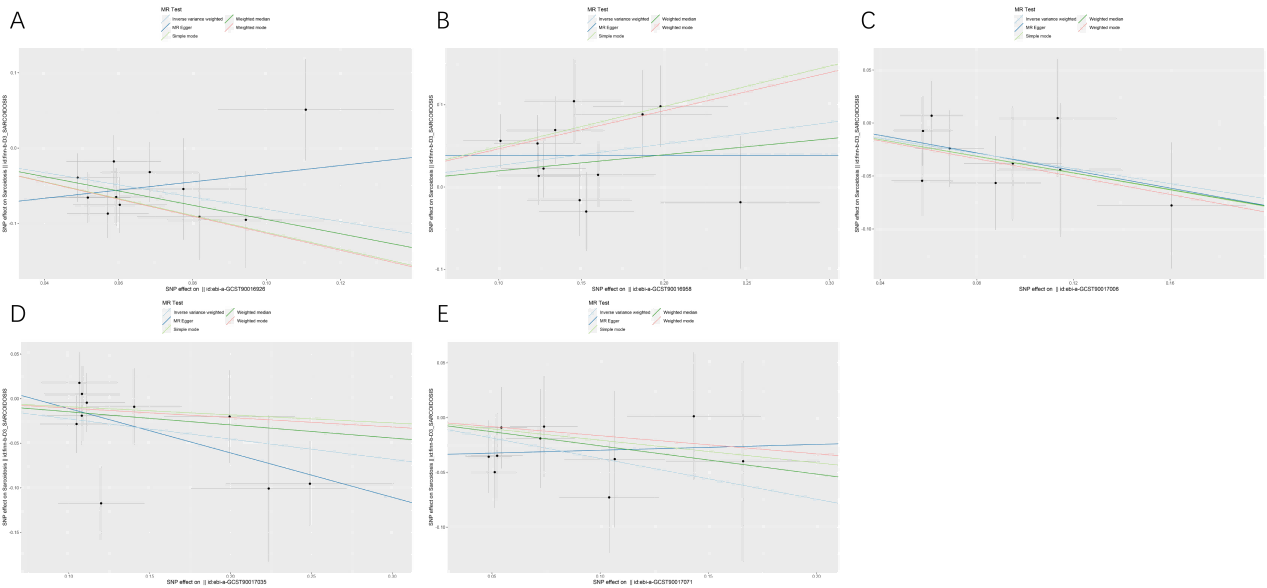
**

Supplement Figure S10

Scatter plots of significant and nominal significant estimates from genetically predicted gut microbiota on Sarcoidosis. (A) family Alcaligenaceae; (B)family Victivallaceae; (C)genus Eubacterium xylanophilum group; (D)genus Olsenella; (E)genus Subdoligranulum

**
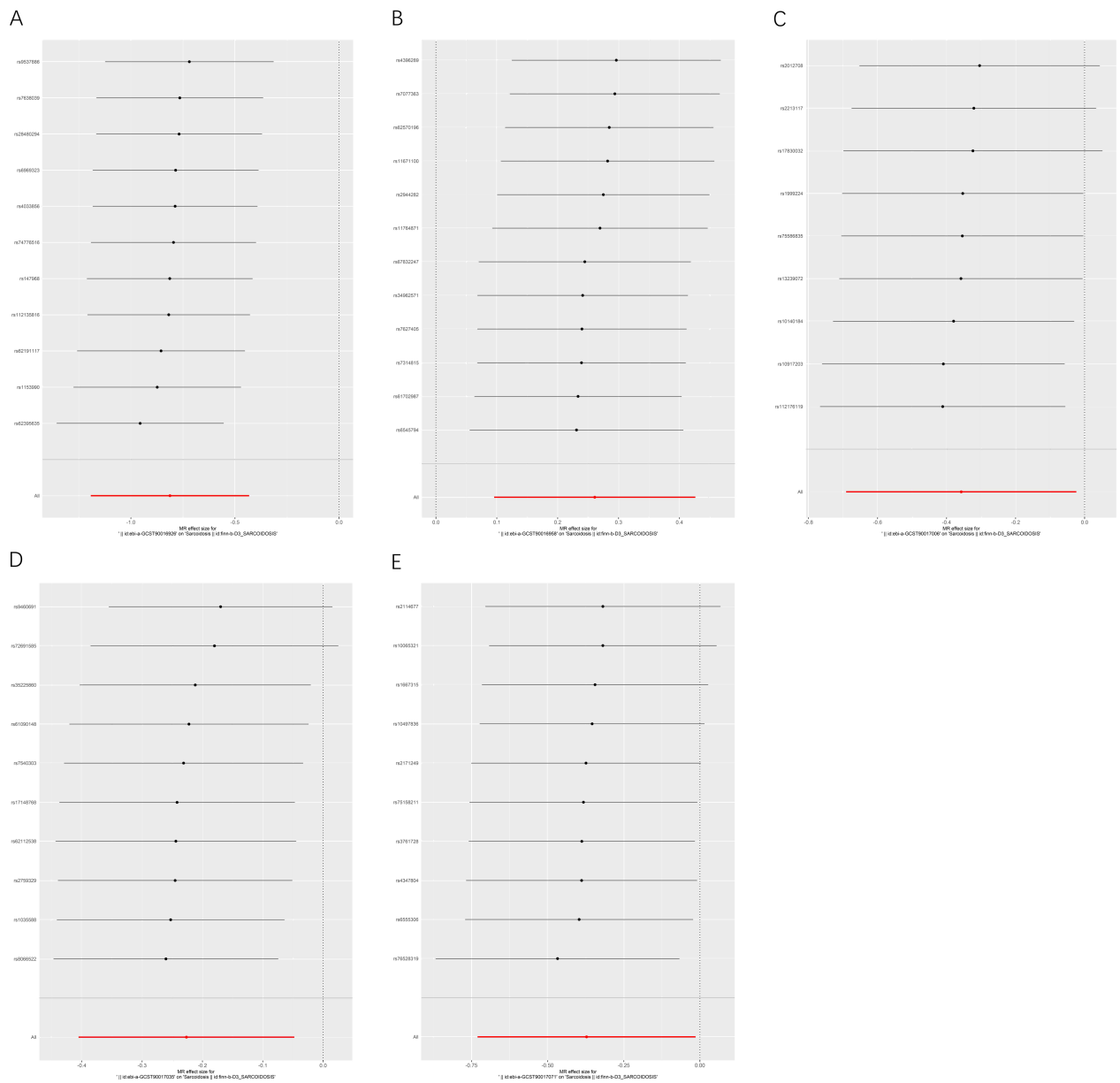
**

Supplement Figure S11

Leave-one-out plots of significant and nominal significant estimates from genetically predicted gut microbiota on Sarcoidosis. (A) family Alcaligenaceae; (B)family Victivallaceae; (C)genus Eubacterium xylanophilum group; (D)genus Olsenella; (E)genus Subdoligranulum

**
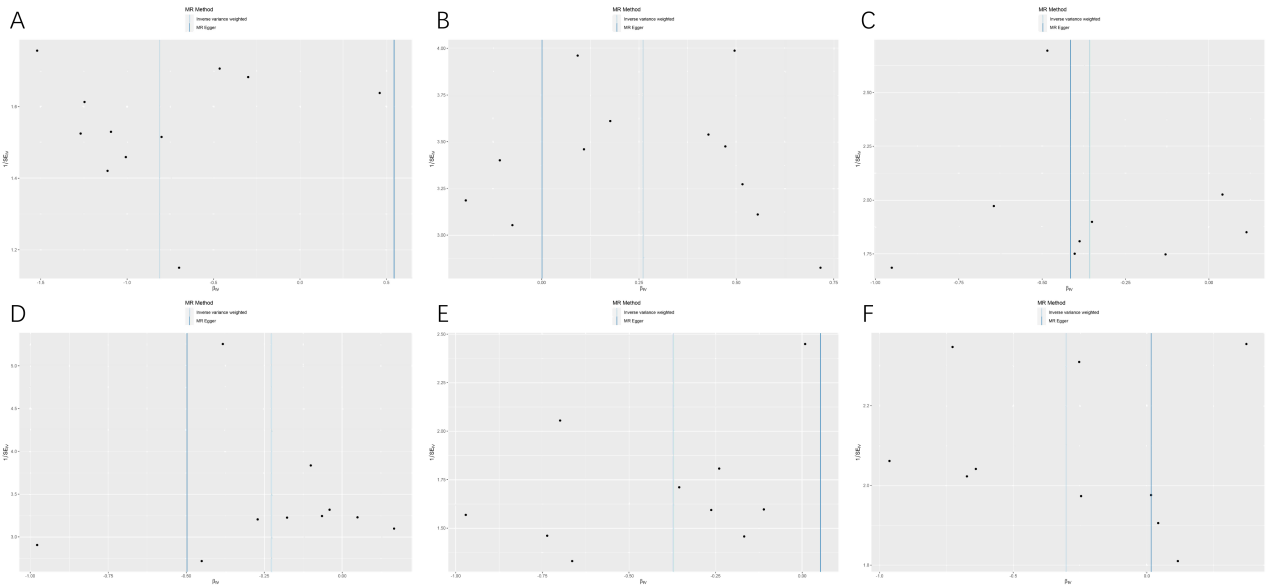
**

Supplement Figure S12

Funnel plots of significant and nominal significant estimates from genetically predicted

gut microbiota on Sarcoidosis. (A) family Alcaligenaceae; (B)family Victivallaceae; (C)genus Eubacterium xylanophilum group; (D)genus Olsenella; (E)genus Subdoligranulum

**
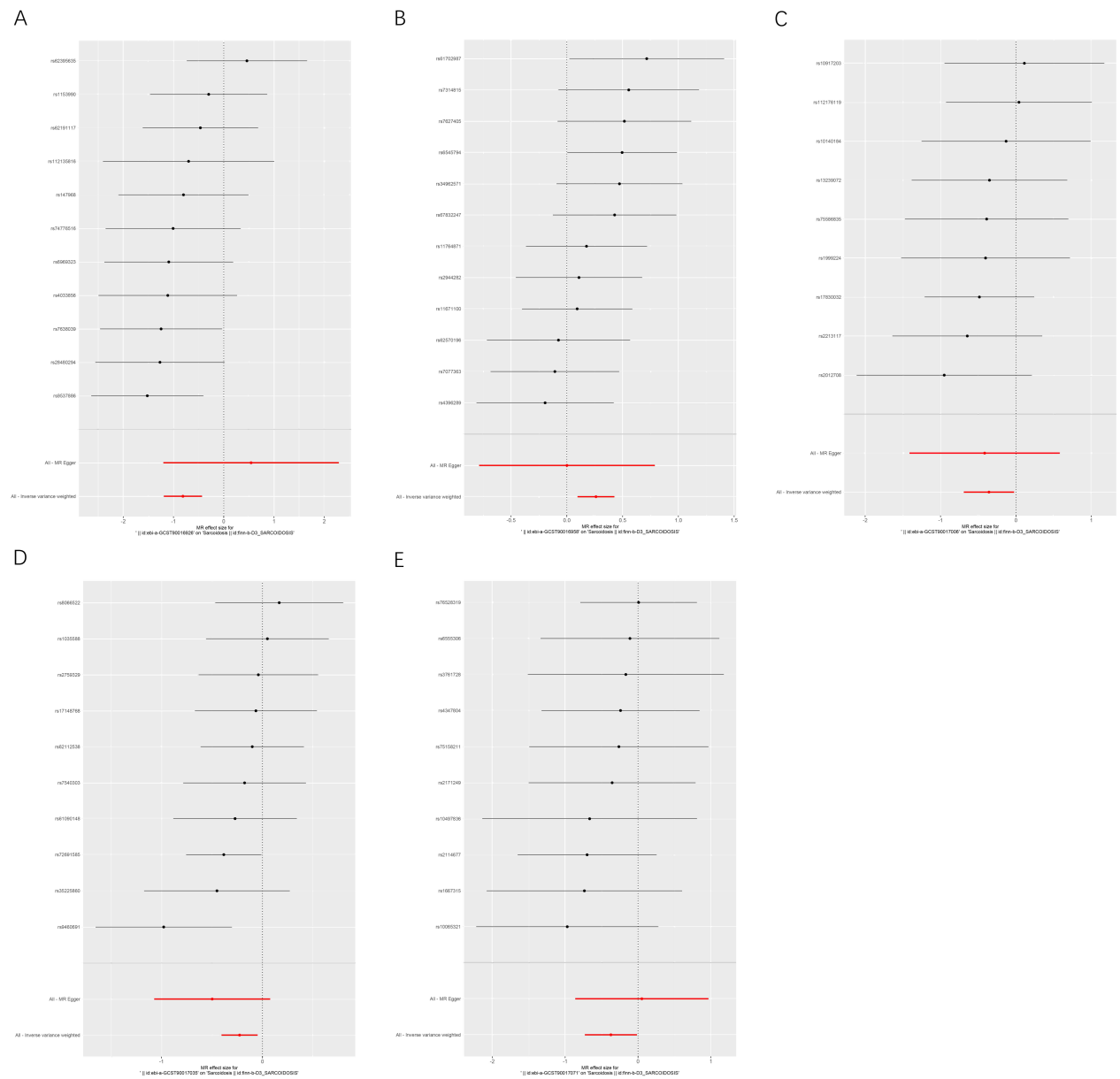
**

Supplement Figure S13

Single plots of significant and nominal significant estimates from genetically predicted

gut microbiota on Sarcoidosis. (A) family Alcaligenaceae; (B)family Victivallaceae; (C)genus Eubacterium xylanophilum group; (D)genus Olsenella; (E)genus Subdoligranulum

**
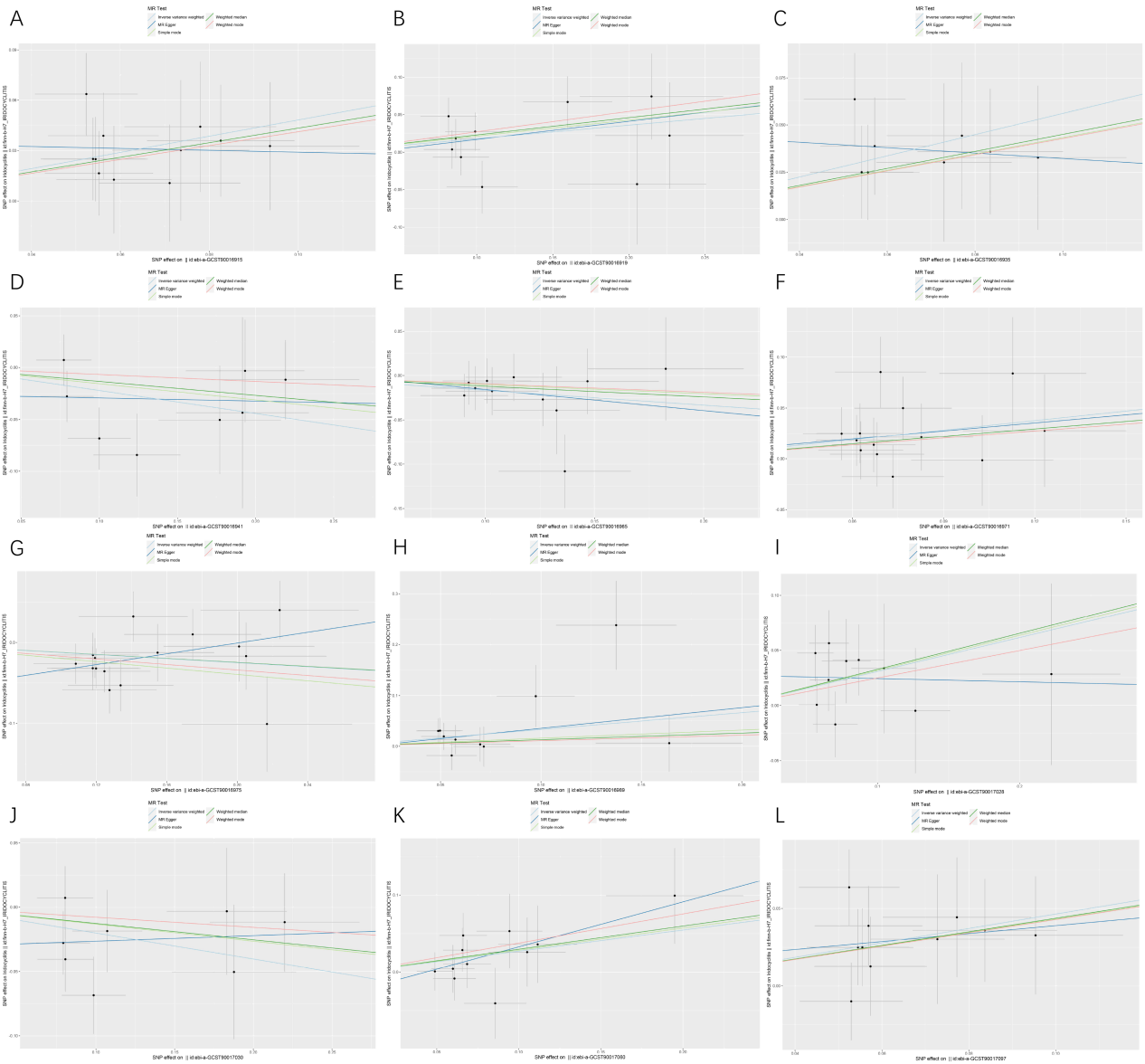
**

Supplement Figure S14

Scatter plots of significant and nominal significant estimates from genetically predicted gut microbiota on Iridocyclitis. (A)class Deltaproteobacteria; (B)class Melainabacteria; (C)family Desulfovibrionaceae; (D)family Lactobacillaceae; (E)genus Anaerofilum; (F)genus Bilophila; (G)genus Butyrivibrio; (H)genus Dorea; (I)genus Lachnospiraceae UCG010; (J)genus Lactobacillus; (K)genus Ruminococcaceae UCG013; (L)order Desulfovibrionales.

**
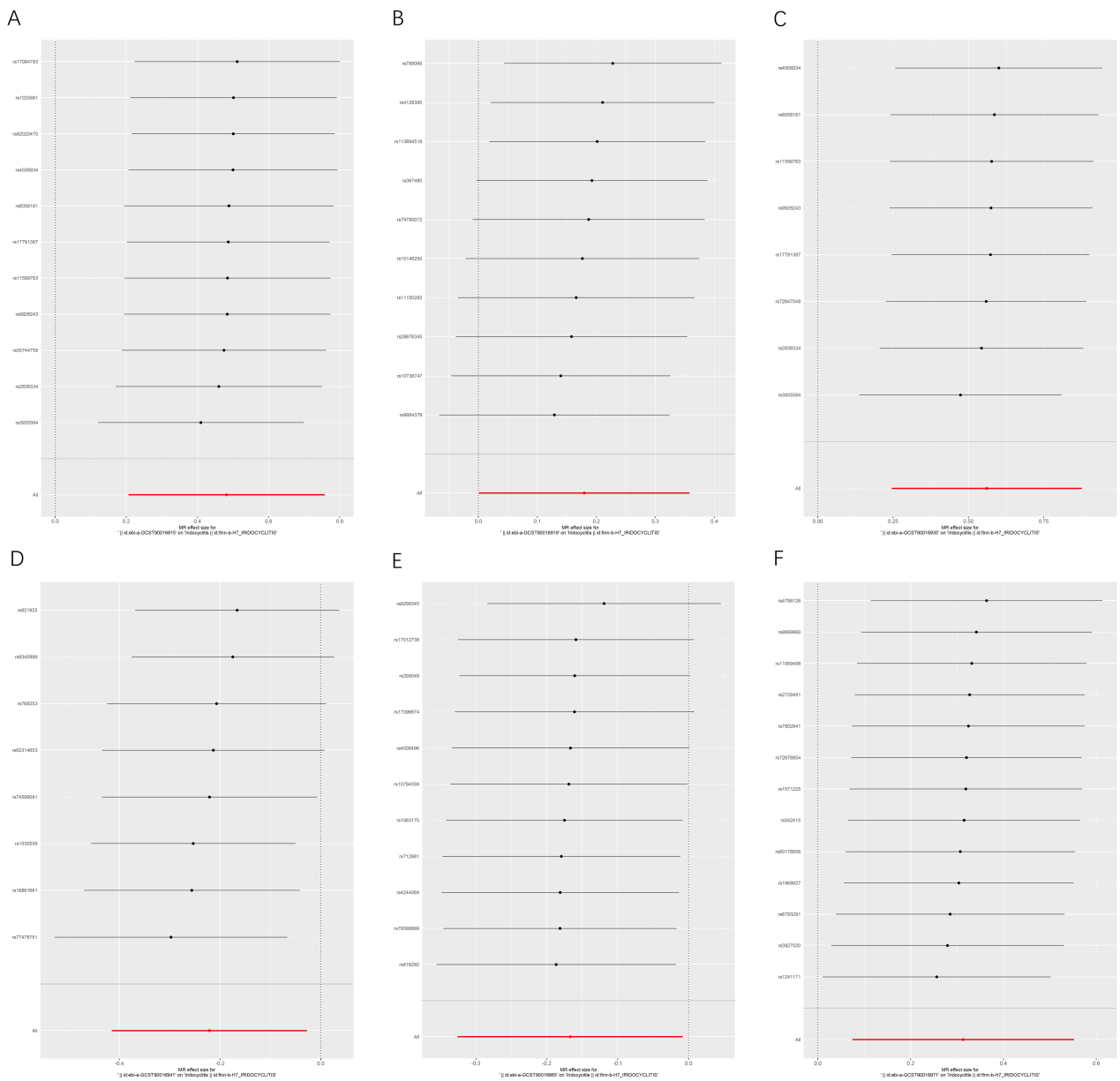
**

**
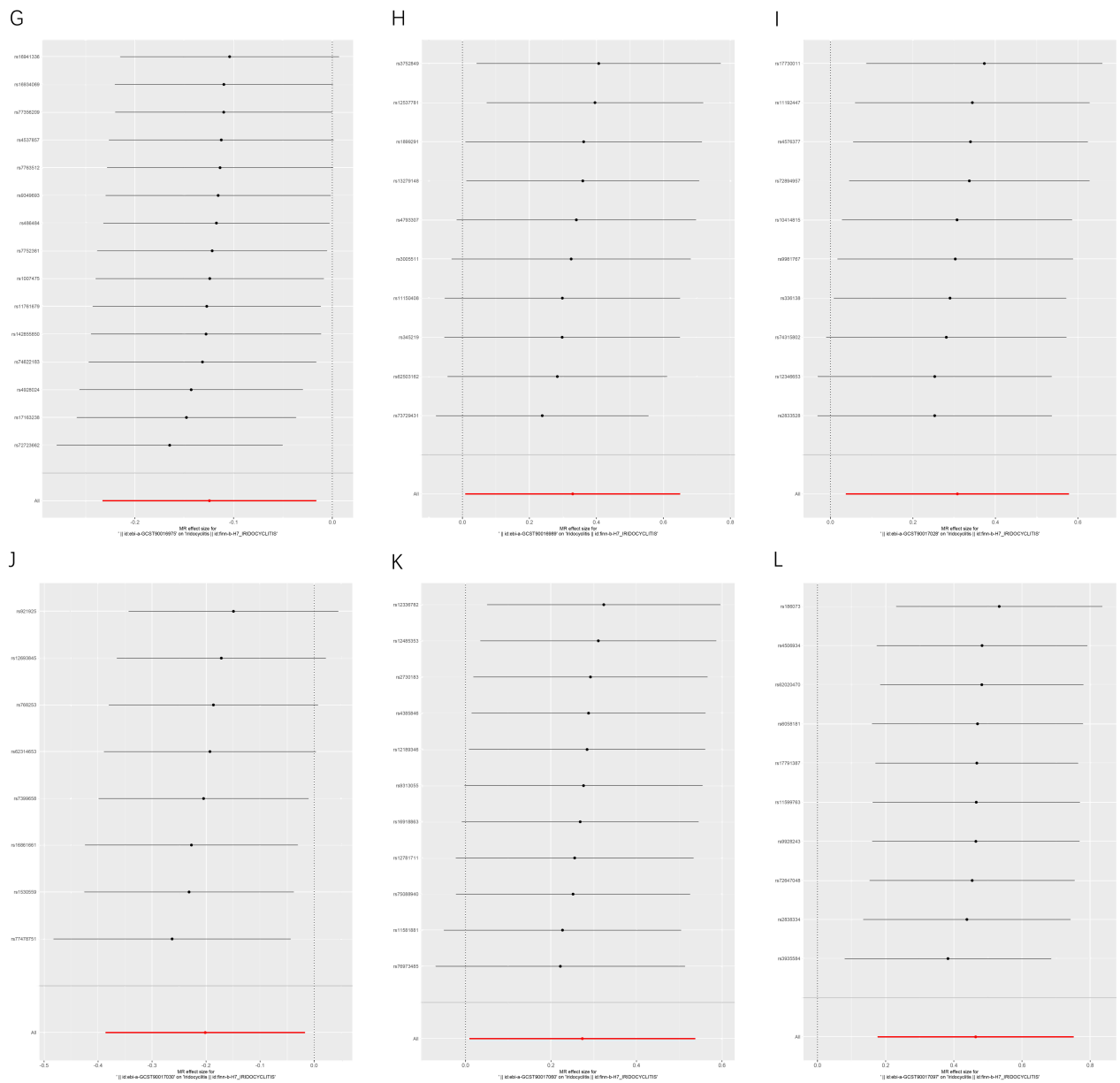
Supplement Figure S15**

Leave-one-out plots of significant and nominal significant estimates from genetically predicted gut microbiota on Iridocyclitis. (A)class Deltaproteobacteria; (B)class Melainabacteria; (C)family Desulfovibrionaceae; (D)family Lactobacillaceae; (E)genus Anaerofilum; (F)genus Bilophila; (G)genus Butyrivibrio; (H)genus Dorea; (I)genus Lachnospiraceae UCG010; (J)genus Lactobacillus; (K)genus Ruminococcaceae UCG013; (L)order Desulfovibrionales.

**
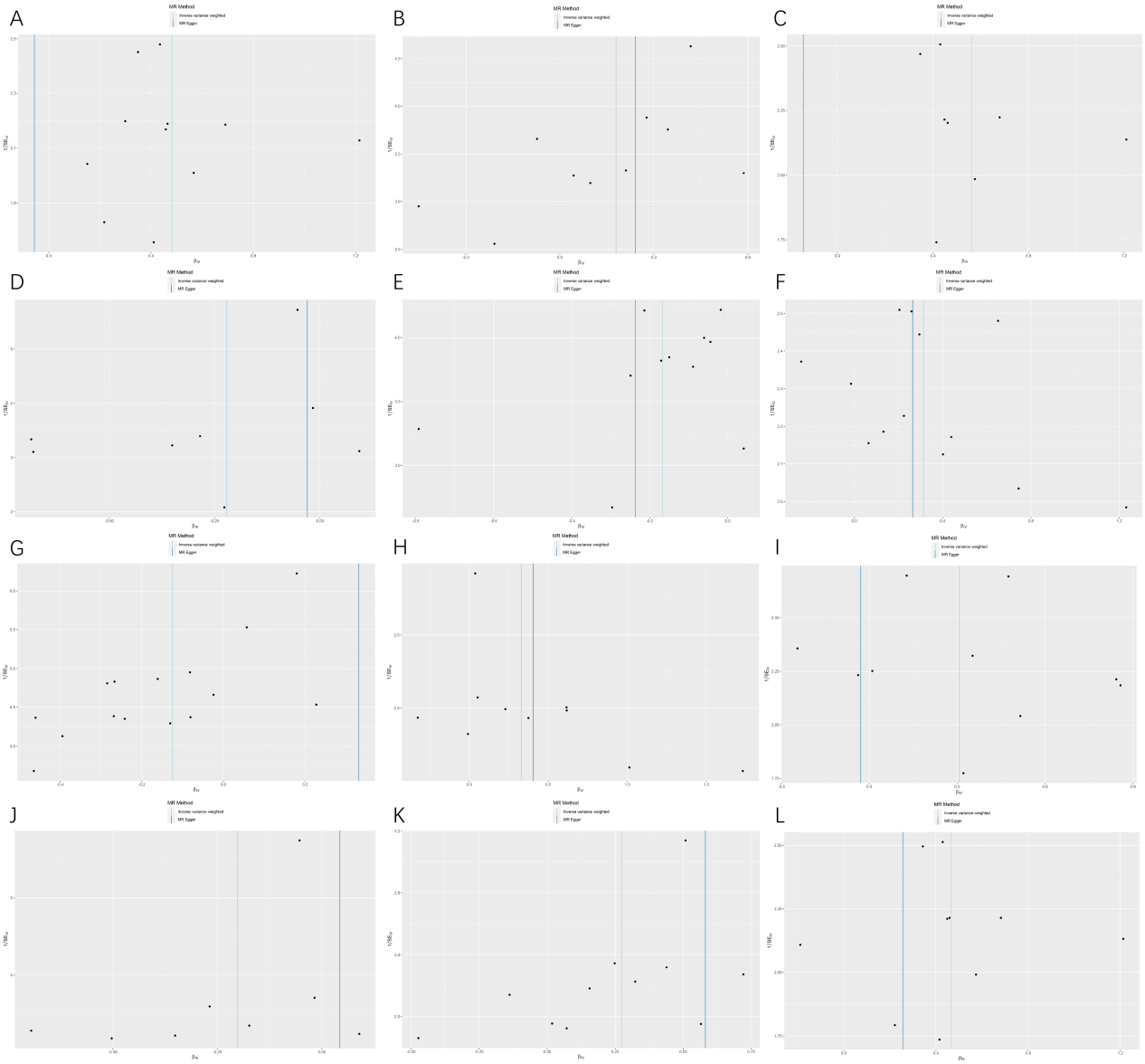
**

Supplement Figure S16

Funnel plots of significant and nominal significant estimates from genetically predicted

gut microbiota on Iridocyclitis. (A)class Deltaproteobacteria; (B)class Melainabacteria; (C)family Desulfovibrionaceae; (D)family Lactobacillaceae; (E)genus Anaerofilum; (F)genus Bilophila; (G)genus Butyrivibrio; (H)genus Dorea; (I)genus Lachnospiraceae UCG010; (J)genus Lactobacillus; (K)genus Ruminococcaceae UCG013; (L)order Desulfovibrionales.


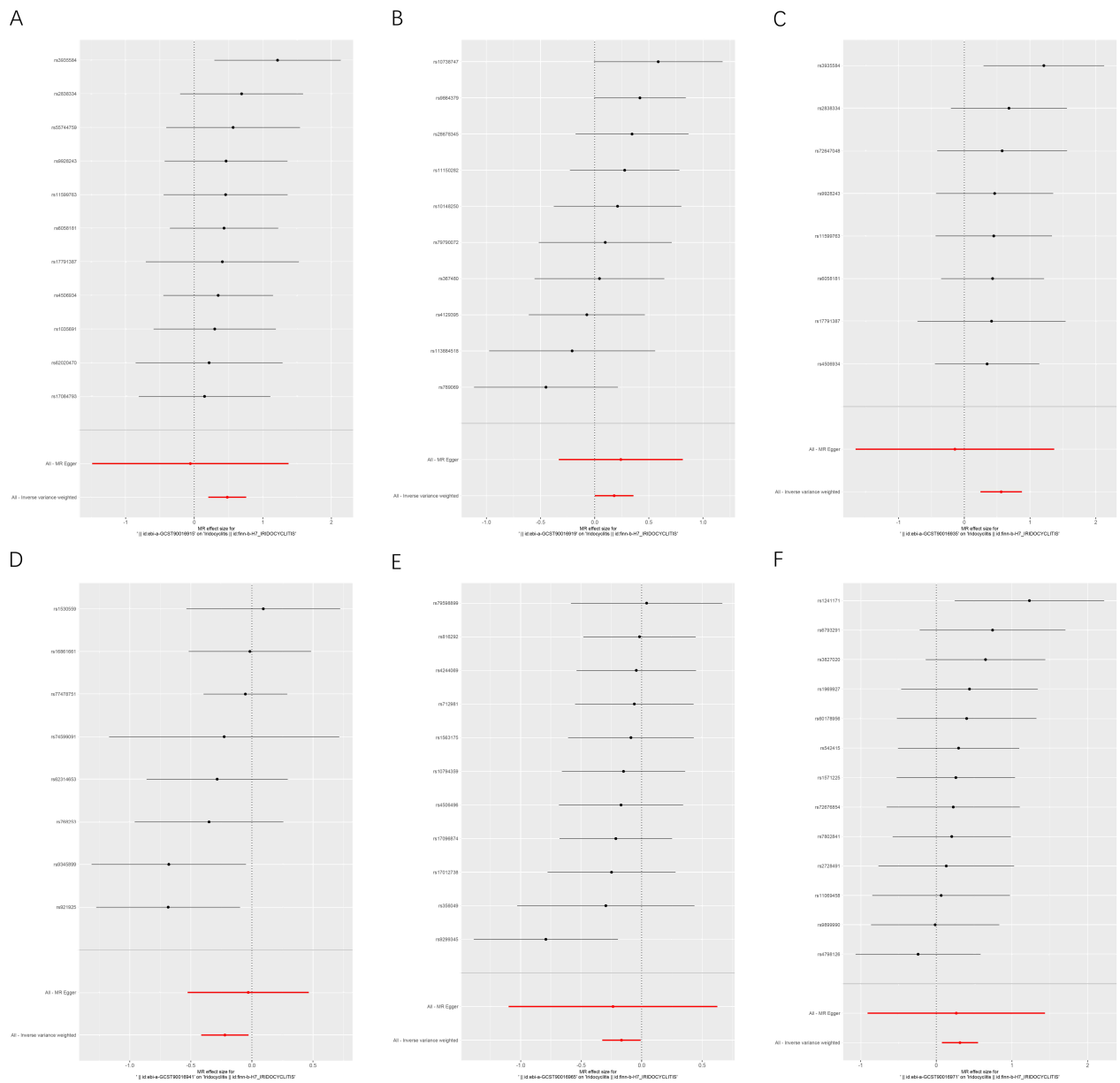


**
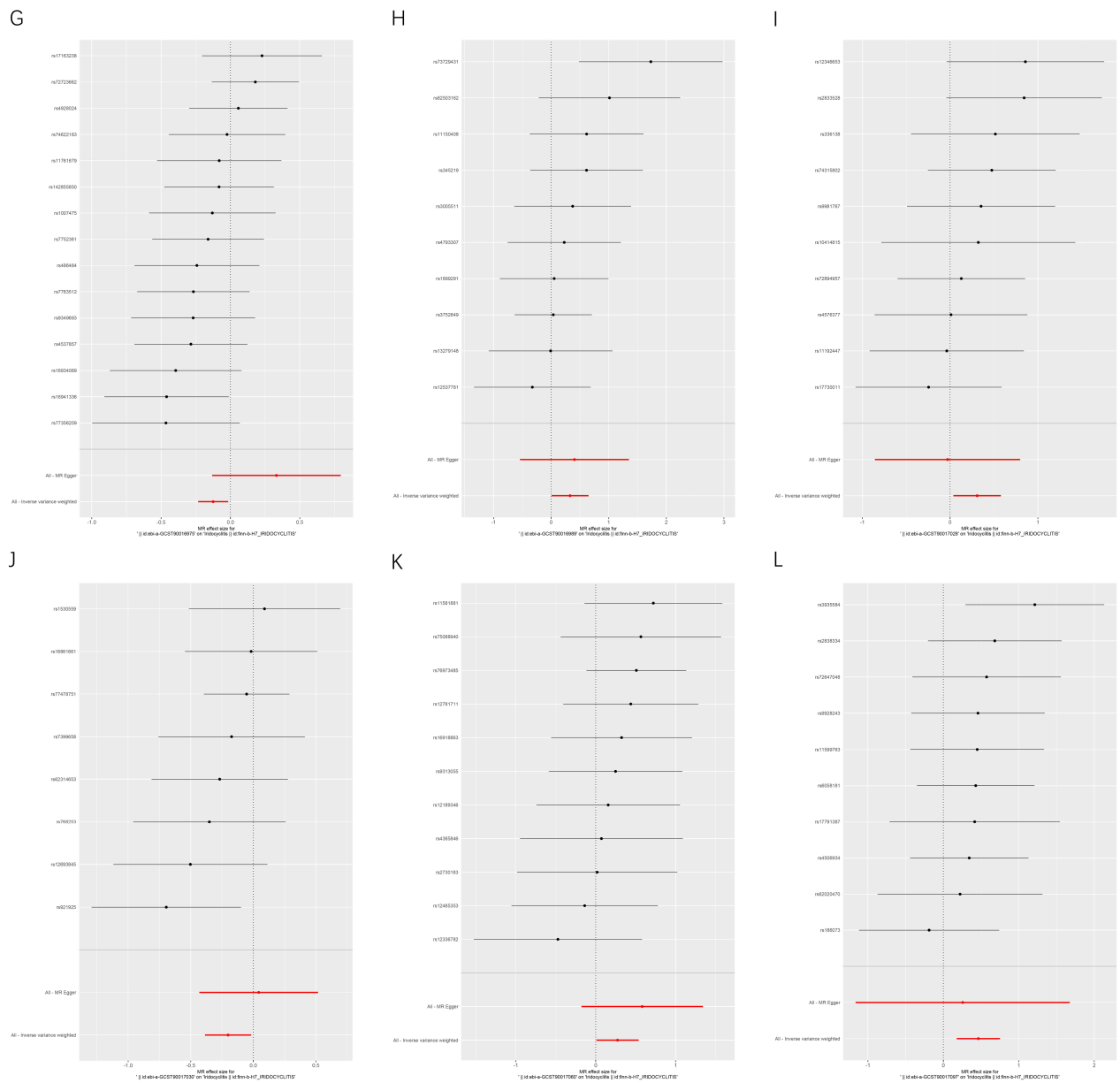
Supplement Figure S17**

Single plots of significant and nominal significant estimates from genetically predicted

gut microbiota on Iridocyclitis. (A)class Deltaproteobacteria; (B)class Melainabacteria; (C)family Desulfovibrionaceae; (D)family Lactobacillaceae; (E)genus Anaerofilum; (F)genus Bilophila; (G)genus Butyrivibrio; (H)genus Dorea; (I)genus Lachnospiraceae UCG010; (J)genus Lactobacillus; (K)genus Ruminococcaceae UCG013; (L)order Desulfovibrionales.

**
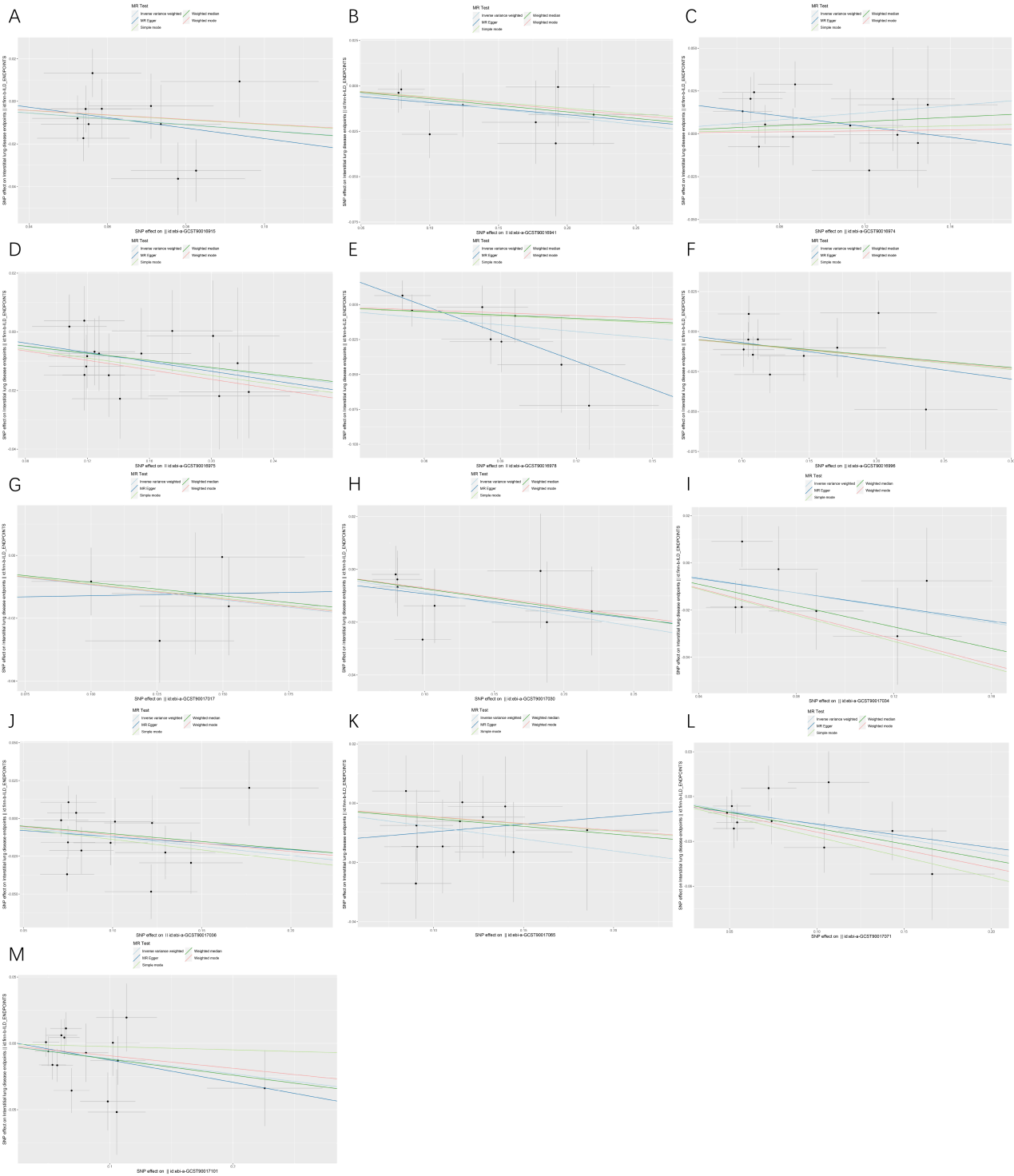
**

Supplement Figure S18

Scatter plots of significant and nominal significant estimates from genetically predicted gut microbiota on Interstitial lung disease. (A)class Deltaproteobacteria; (B)family Lactobacillaceae; (C)genus Butyricimonas; (D)genus Butyrivibrio; (E)genus Christensenellaceae R 7group; (F)genus Eubacterium brachy group; (G)genus Hungatella; (H)genus Lactobacillus; (I)genus Odoribacter; (J)genus Oscillibacter; (K)genus Ruminococcus gnavus group; (L)genus Subdoligranulum; (M)order Lactobacillales.

**
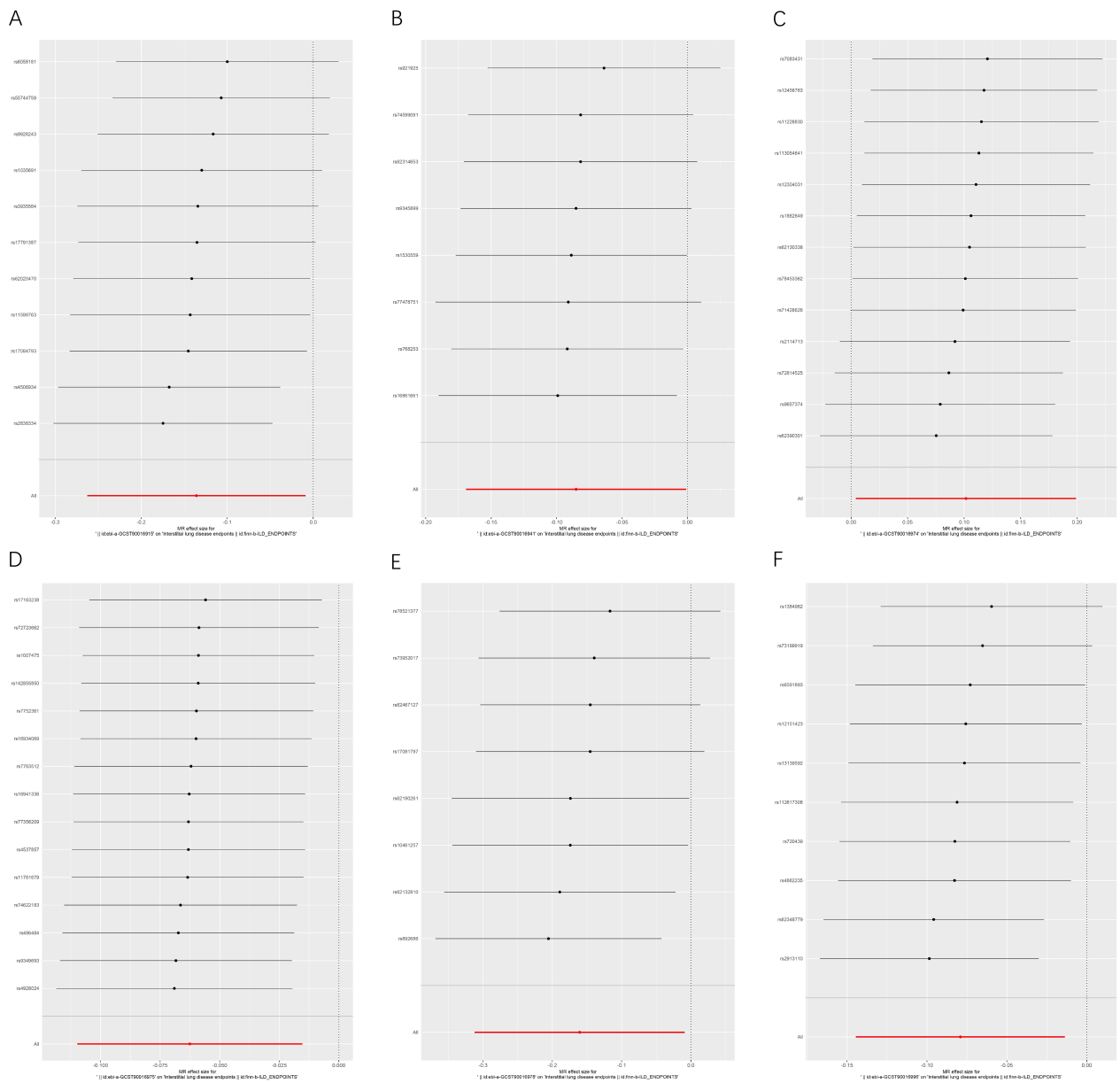
**

**
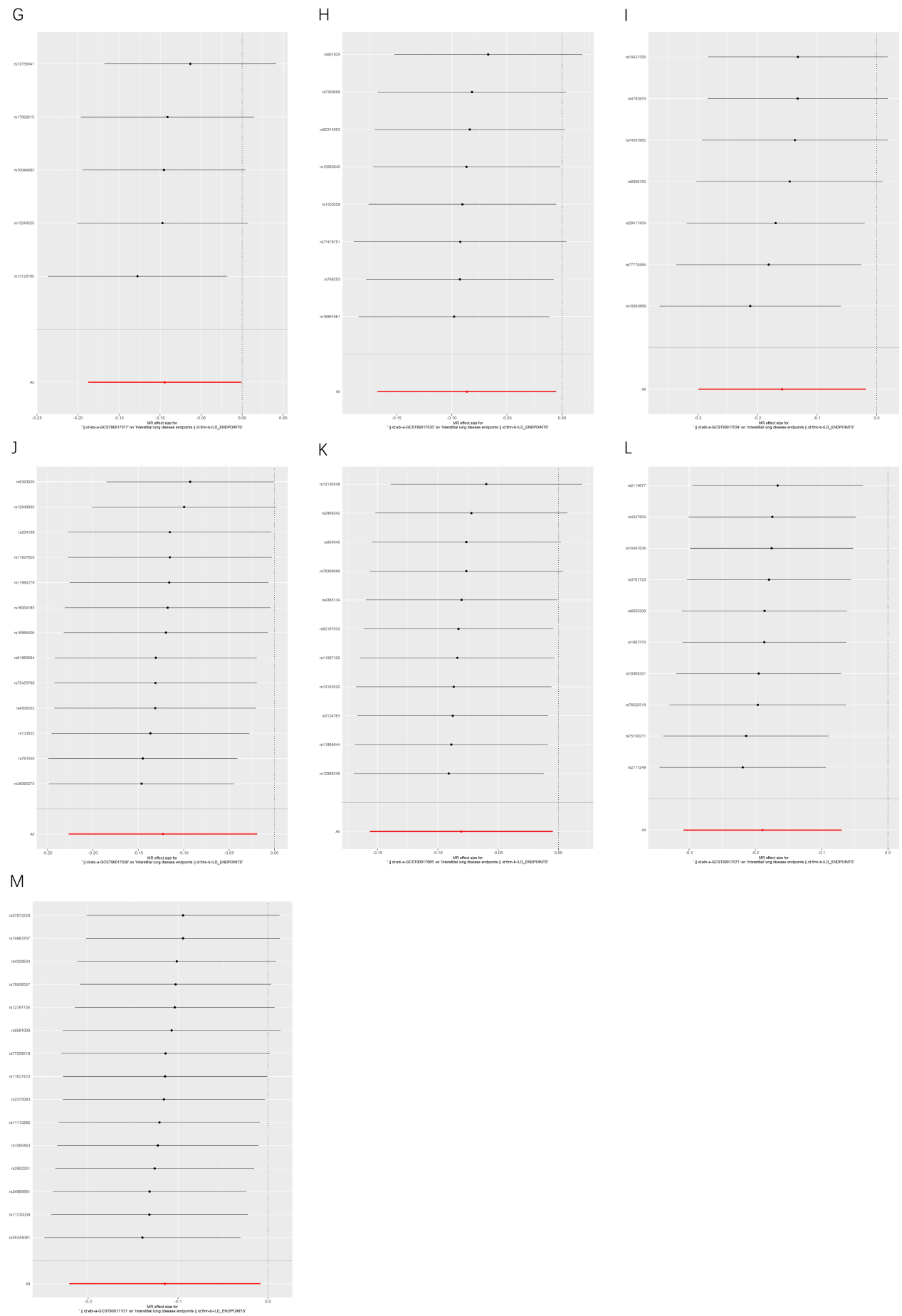
**

Supplement Figure S19

Leave-one-out plots of significant and nominal significant estimates from genetically predicted gut microbiota on Interstitial lung disease. (A)class Deltaproteobacteria; (B)family Lactobacillaceae; (C)genus Butyricimonas; (D)genus Butyrivibrio; (E)genus Christensenellaceae R 7group; (F)genus Eubacterium brachy group; (G)genus Hungatella; (H)genus Lactobacillus; (I)genus Odoribacter; (J)genus Oscillibacter; (K)genus Ruminococcus gnavus group; (L)genus Subdoligranulum; (M)order Lactobacillales.

**
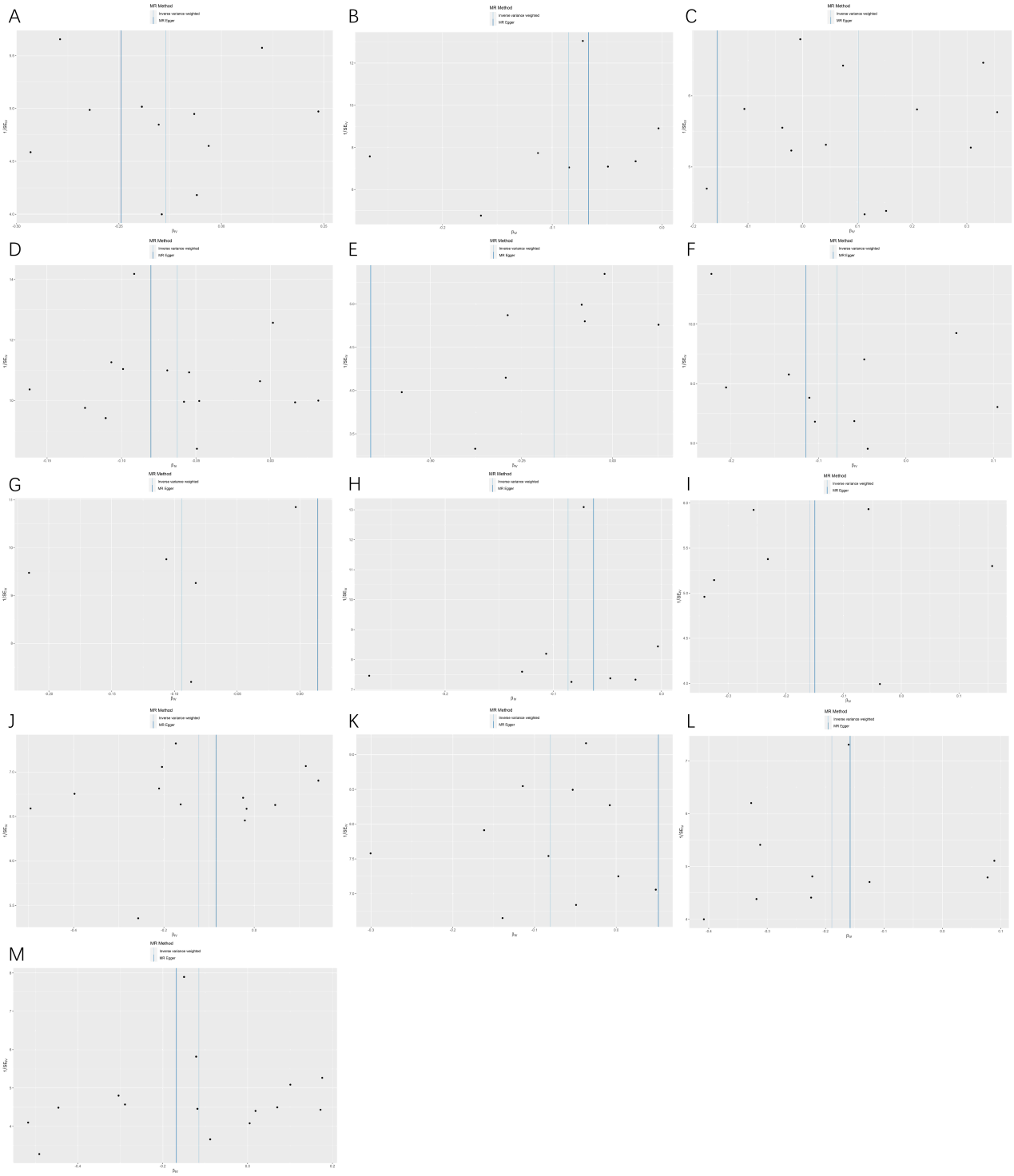
**

Supplement Figure S20

Funnel plots of significant and nominal significant estimates from genetically predicted

gut microbiota on Interstitial lung disease. (A)class Deltaproteobacteria; (B)family Lactobacillaceae; (C)genus Butyricimonas; (D)genus Butyrivibrio; (E)genus Christensenellaceae R 7group; (F)genus Eubacterium brachy group; (G)genus Hungatella; (H)genus Lactobacillus; (I)genus Odoribacter; (J)genus Oscillibacter; (K)genus Ruminococcus gnavus group; (L)genus Subdoligranulum; (M)order Lactobacillales.

**
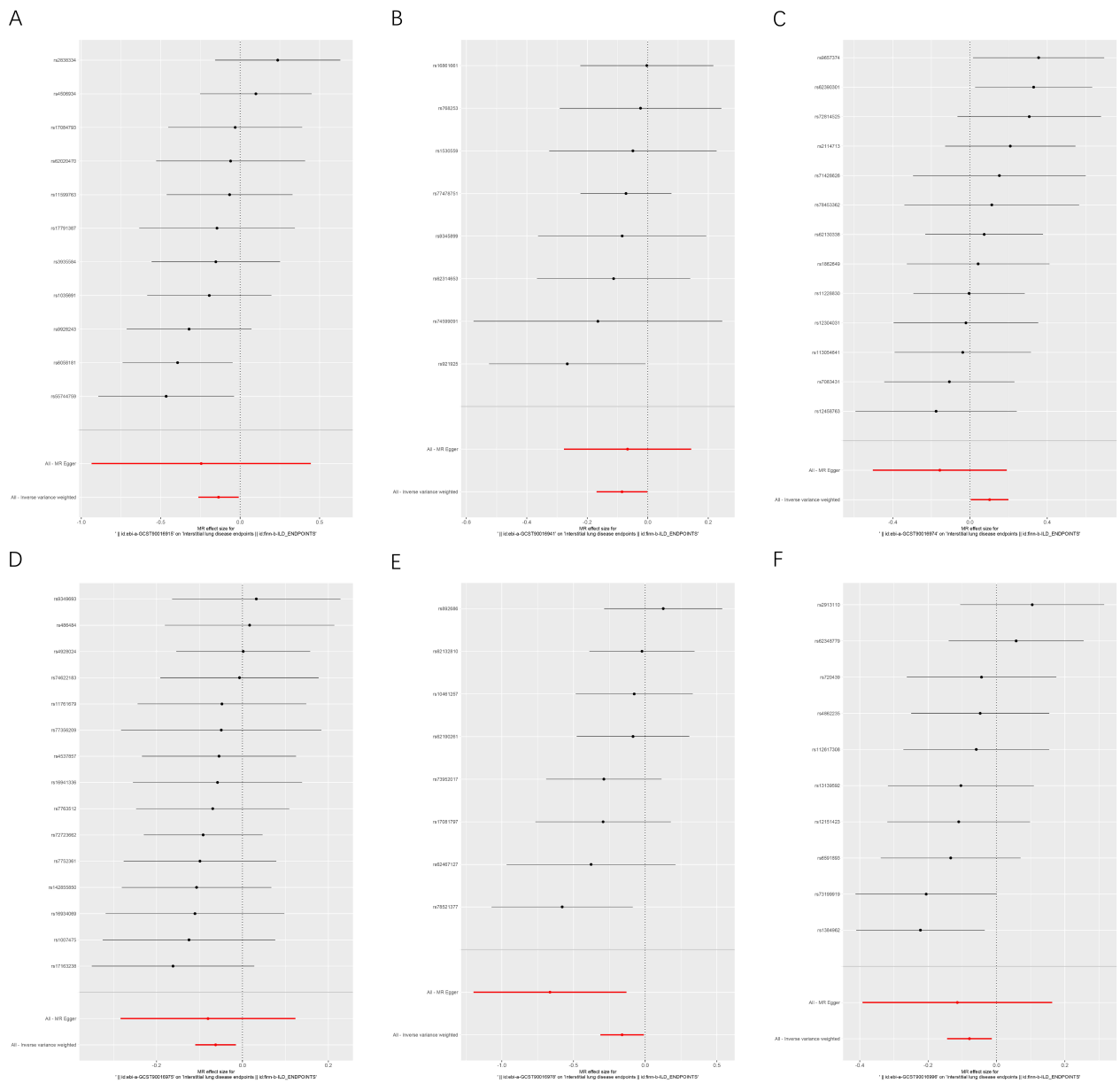

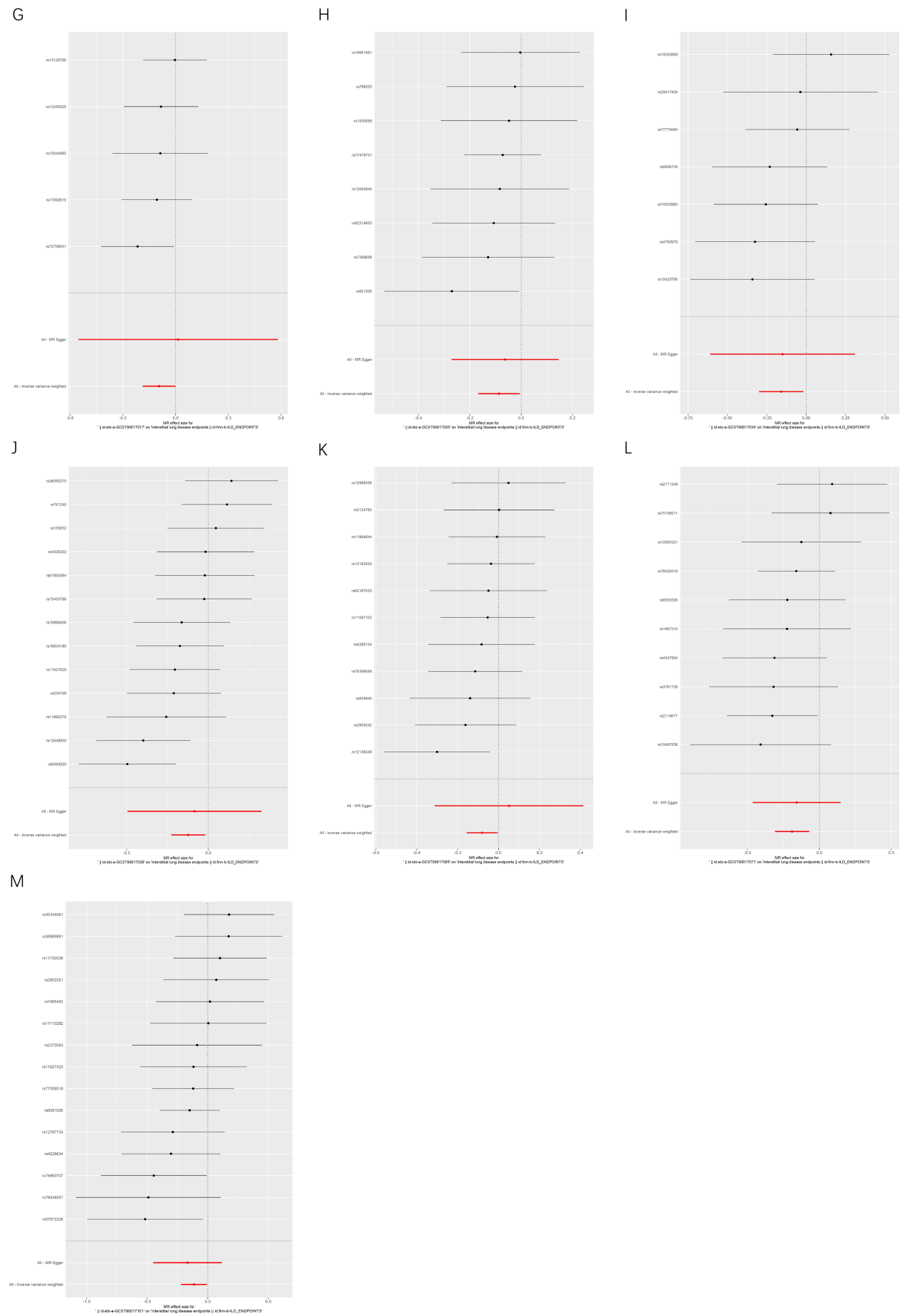
**

Supplement Figure S21

Single plots of significant and nominal significant estimates from genetically predicted

gut microbiota on Interstitial lung disease. (A)class Deltaproteobacteria; (B)family Lactobacillaceae; (C)genus Butyricimonas; (D)genus Butyrivibrio; (E)genus Christensenellaceae R 7group; (F)genus Eubacterium brachy group; (G)genus Hungatella; (H)genus Lactobacillus; (I)genus Odoribacter; (J)genus Oscillibacter; (K)genus Ruminococcus gnavus group; (L)genus Subdoligranulum; (M)order Lactobacillales.

**
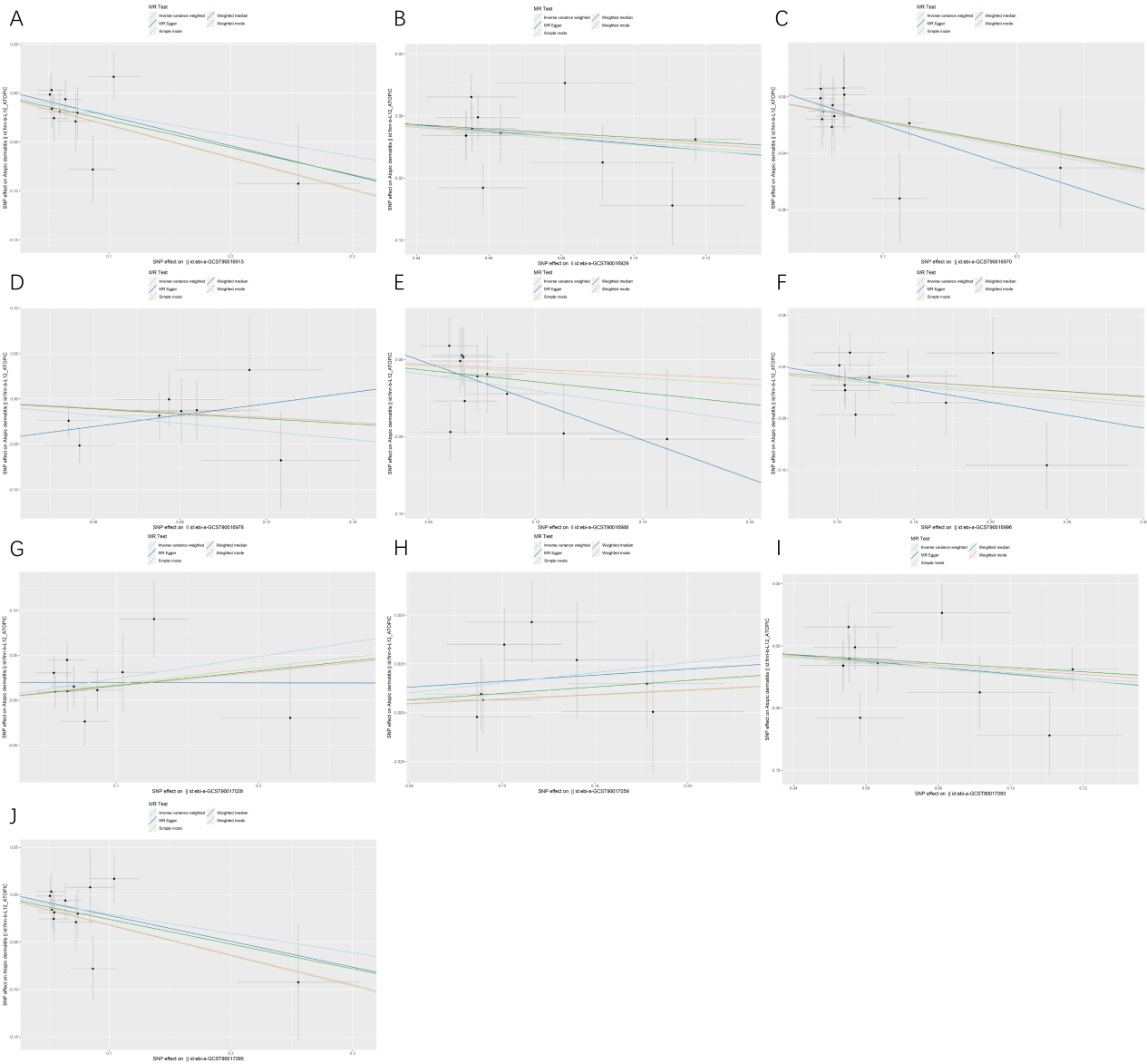
**

Supplement Figure S22

Scatter plots of significant and nominal significant estimates from genetically predicted gut microbiota on Atopic dermatitis. (A)class Clostridia; (B)family Bifidobacteriaceae; (C)genus Bifidobacterium; (D)genus Christensenellaceae R 7group; (E)genus Dialister; (F)genus Eubacterium brachy group; (G)genus Lachnospiraceae UCG010; (H)genus Ruminococcaceae UCG011; (I)order Bifidobacteriales; (J)order Clostridiales.

**
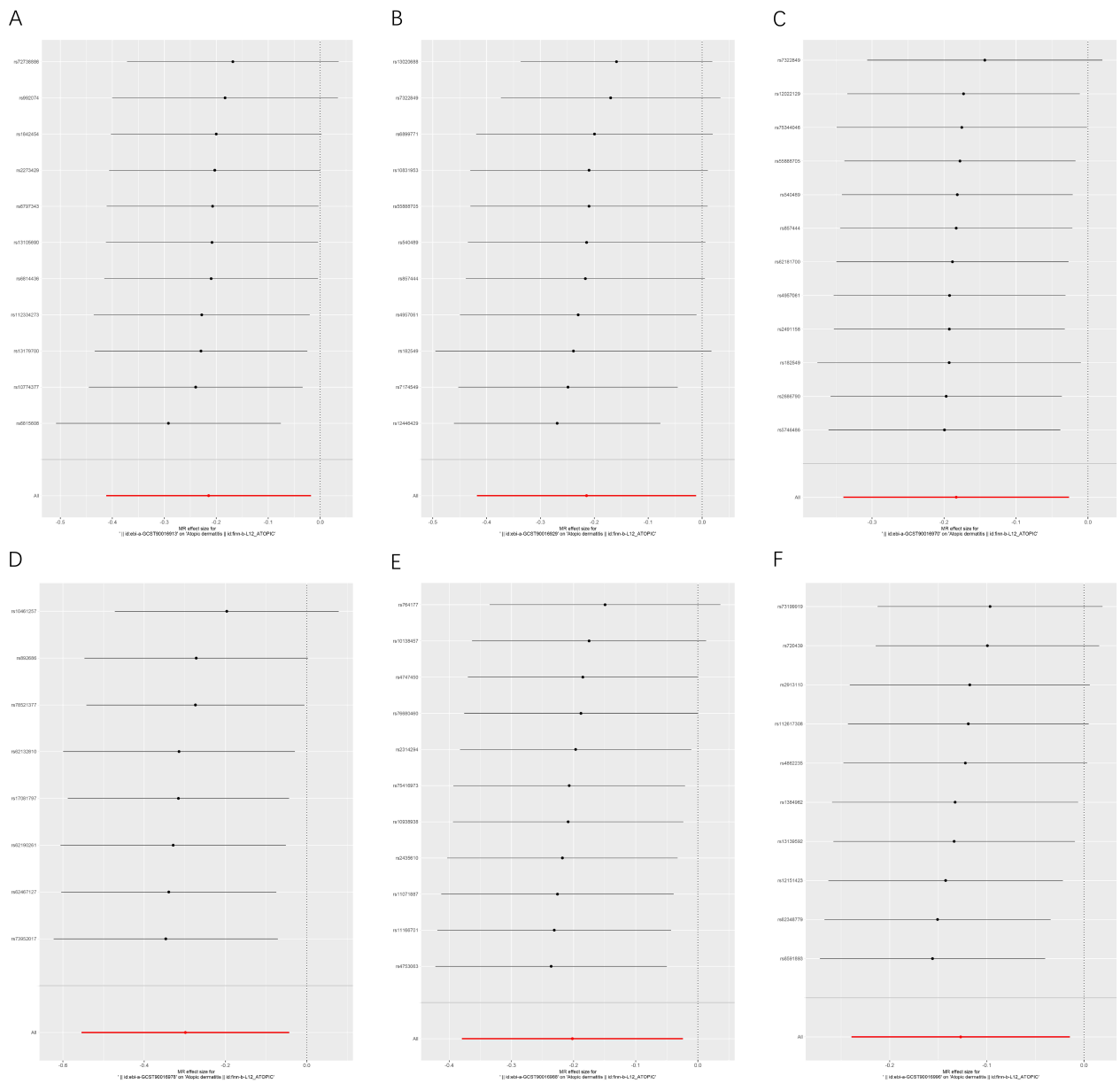
**

**
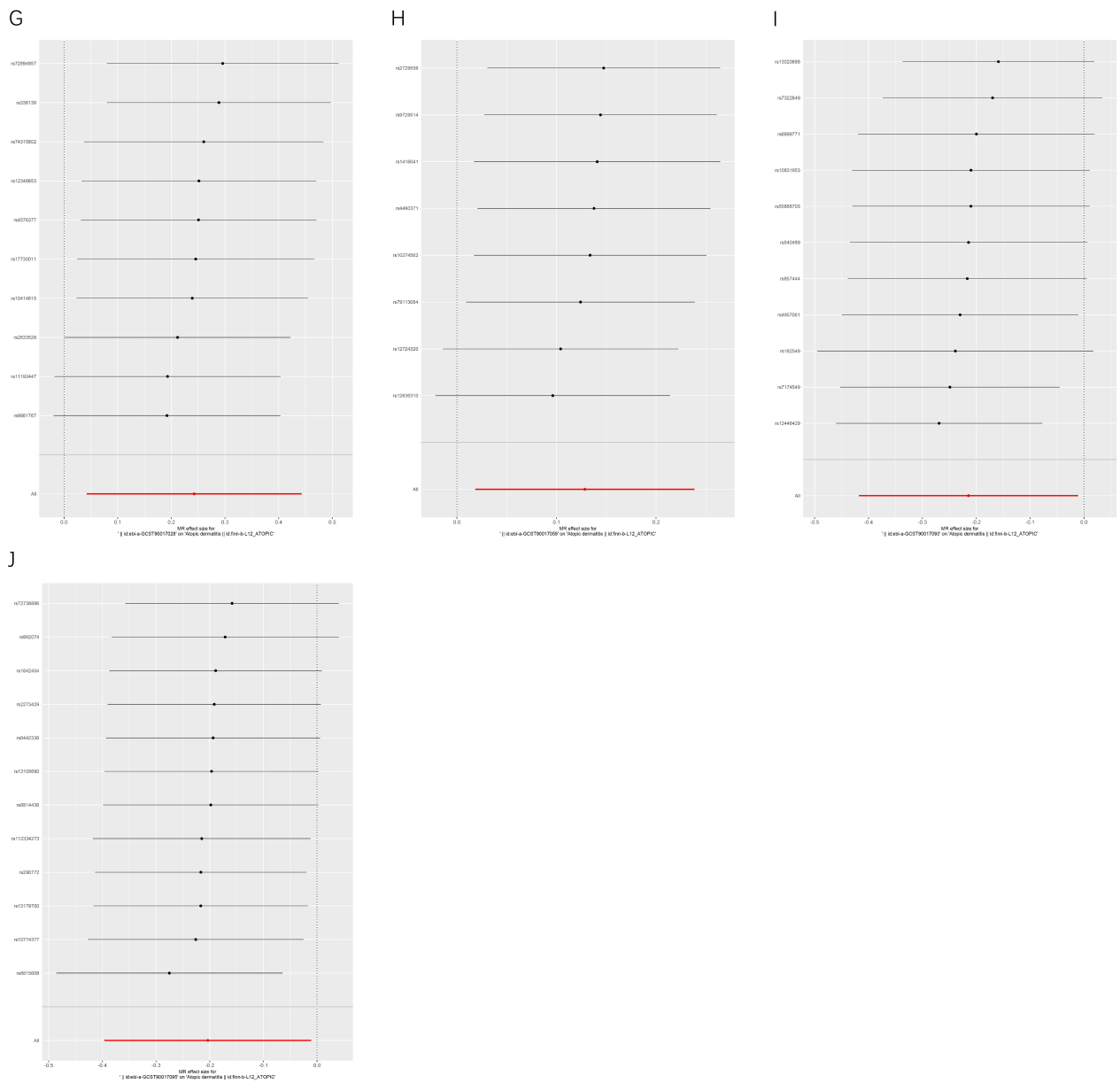
**

Supplement Figure S23

Leave-one-out plots of significant and nominal significant estimates from genetically predicted gut microbiota on Atopic dermatitis. (A)class Clostridia; (B)family Bifidobacteriaceae; (C)genus Bifidobacterium; (D)genus Christensenellaceae R 7group; (E)genus Dialister; (F)genus Eubacterium brachy group; (G)genus Lachnospiraceae UCG010; (H)genus Ruminococcaceae UCG011; (I)order Bifidobacteriales; (J)order Clostridiales.

**
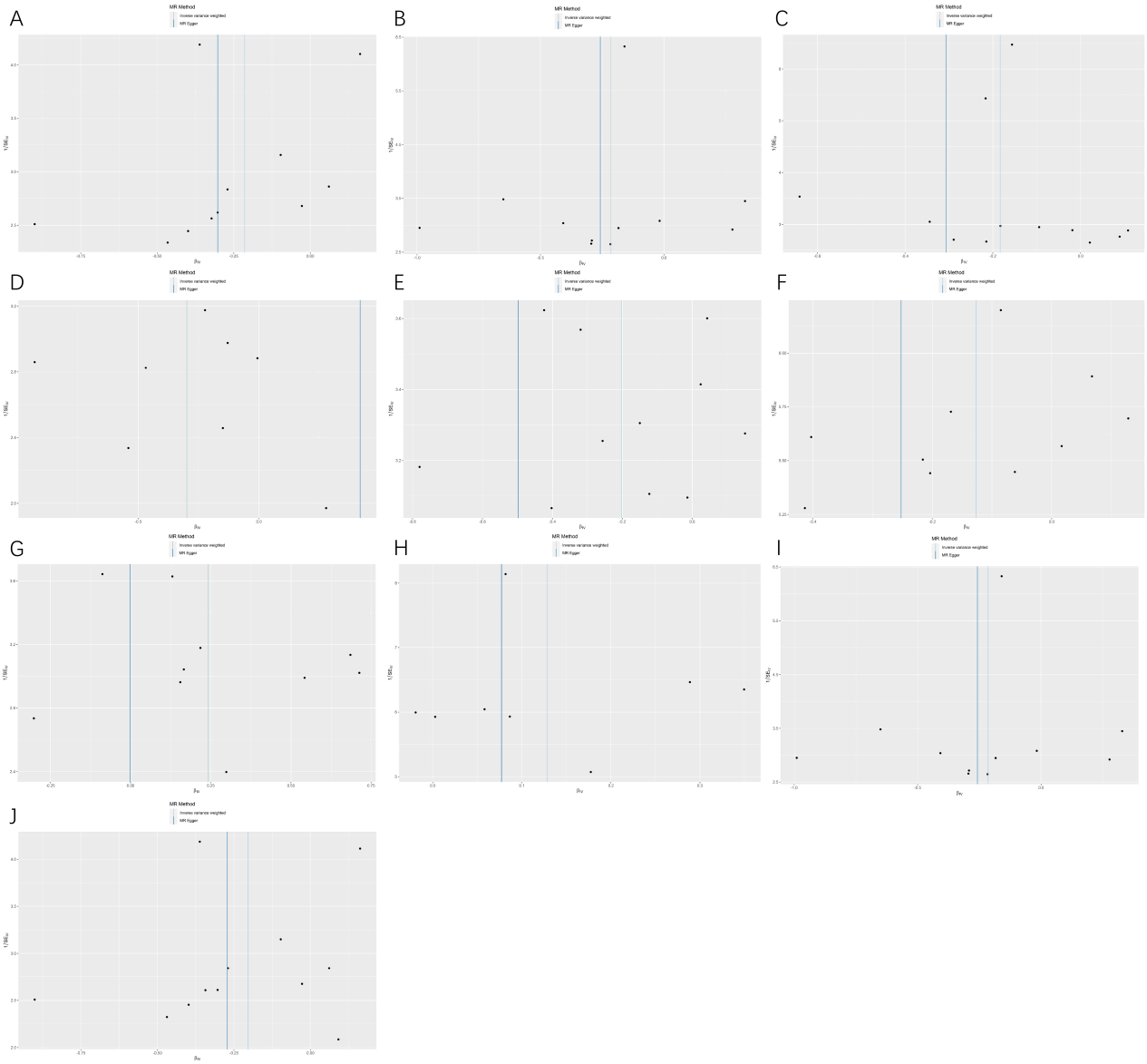
**

Supplement Figure S24

Funnel plots of significant and nominal significant estimates from genetically predicted

gut microbiota on Atopic dermatitis. (A)class Clostridia; (B)family Bifidobacteriaceae; (C)genus Bifidobacterium; (D)genus Christensenellaceae R 7group; (E)genus Dialister; (F)genus Eubacterium brachy group; (G)genus Lachnospiraceae UCG010; (H)genus Ruminococcaceae UCG011; (I)order Bifidobacteriales; (J)order Clostridiales.

**
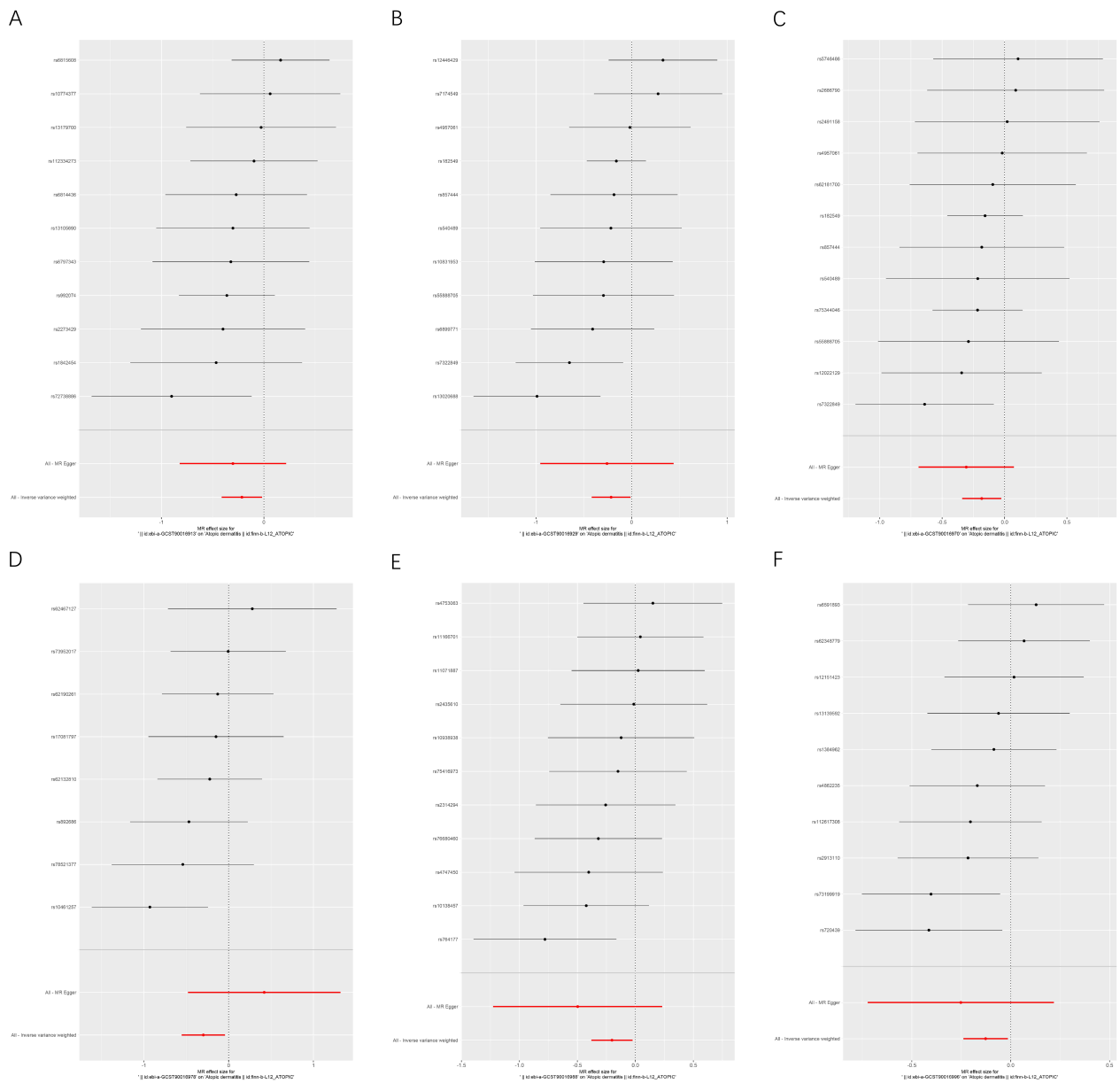
**

**
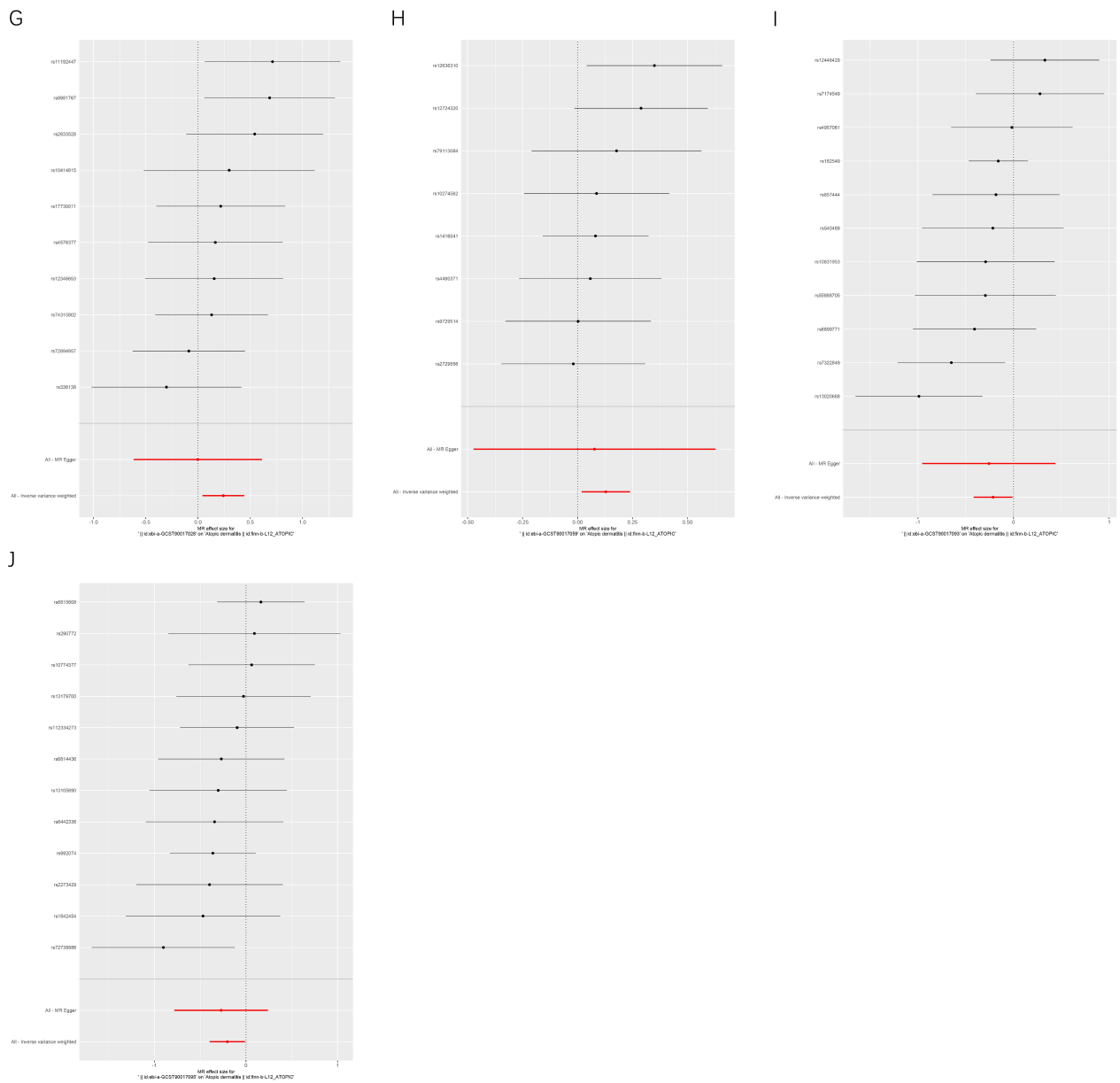
**

Supplement Figure S25

Single plots of significant and nominal significant estimates from genetically predicted

gut microbiota on Atopic dermatitis. (A)class Clostridia; (B)family Bifidobacteriaceae; (C)genus Bifidobacterium; (D)genus Christensenellaceae R 7group; (E)genus Dialister; (F)genus Eubacterium brachy group; (G)genus Lachnospiraceae UCG010; (H)genus Ruminococcaceae UCG011; (I)order Bifidobacteriales; (J)order Clostridiales.

**
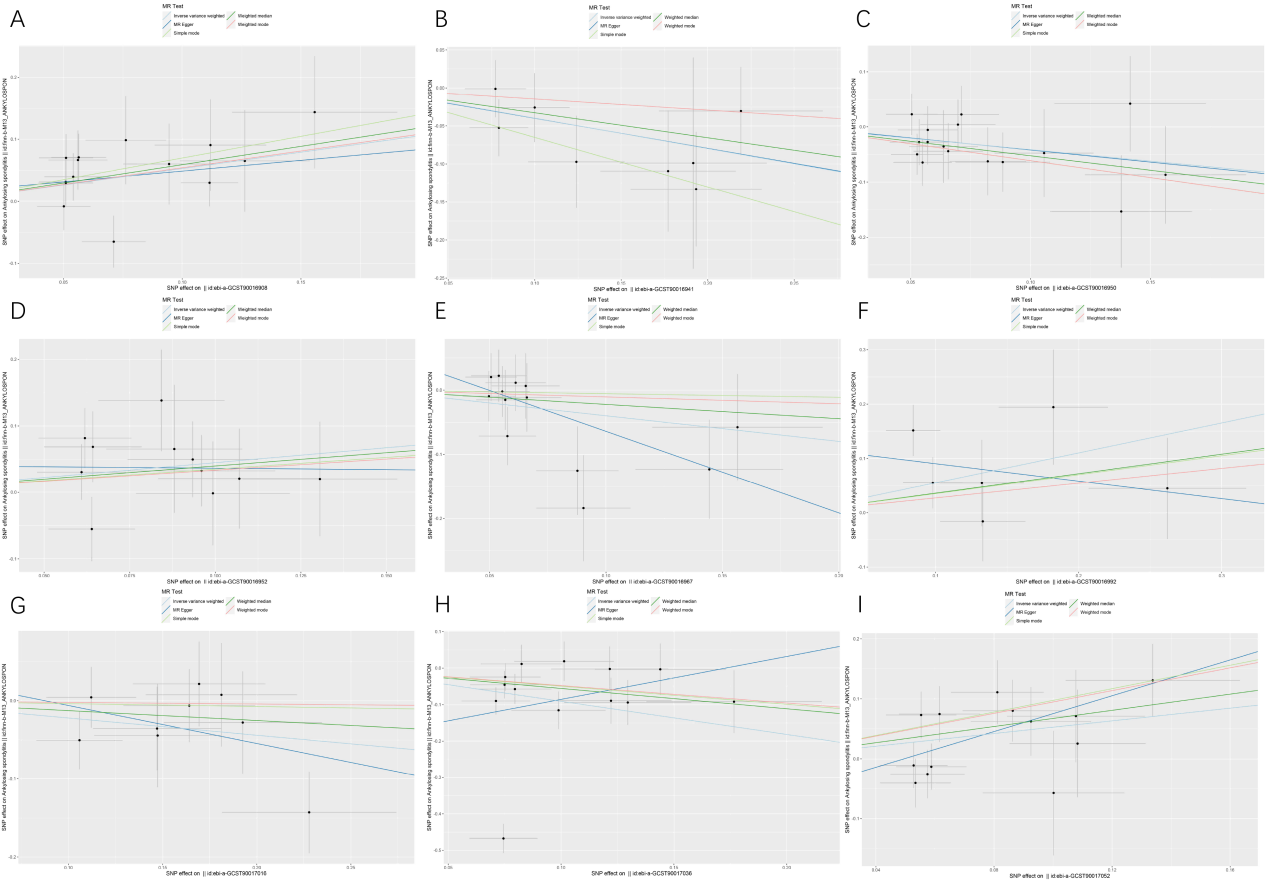
**

Supplement Figure S26

Scatter plots of significant and nominal significant estimates from genetically predicted gut microbiota on Ankylosing spondylitis. (A)class Actinobacteria; (B)family Lactobacillaceae; (C)family Rikenellaceae; (D)family Streptococcaceae; (E)genus Anaerotruncus; (F)genus Enterorhabdus; (G)genus Howardella; (H)genus Oscillibacter; (I)genus Ruminococcaceae NK4A214 group.

**
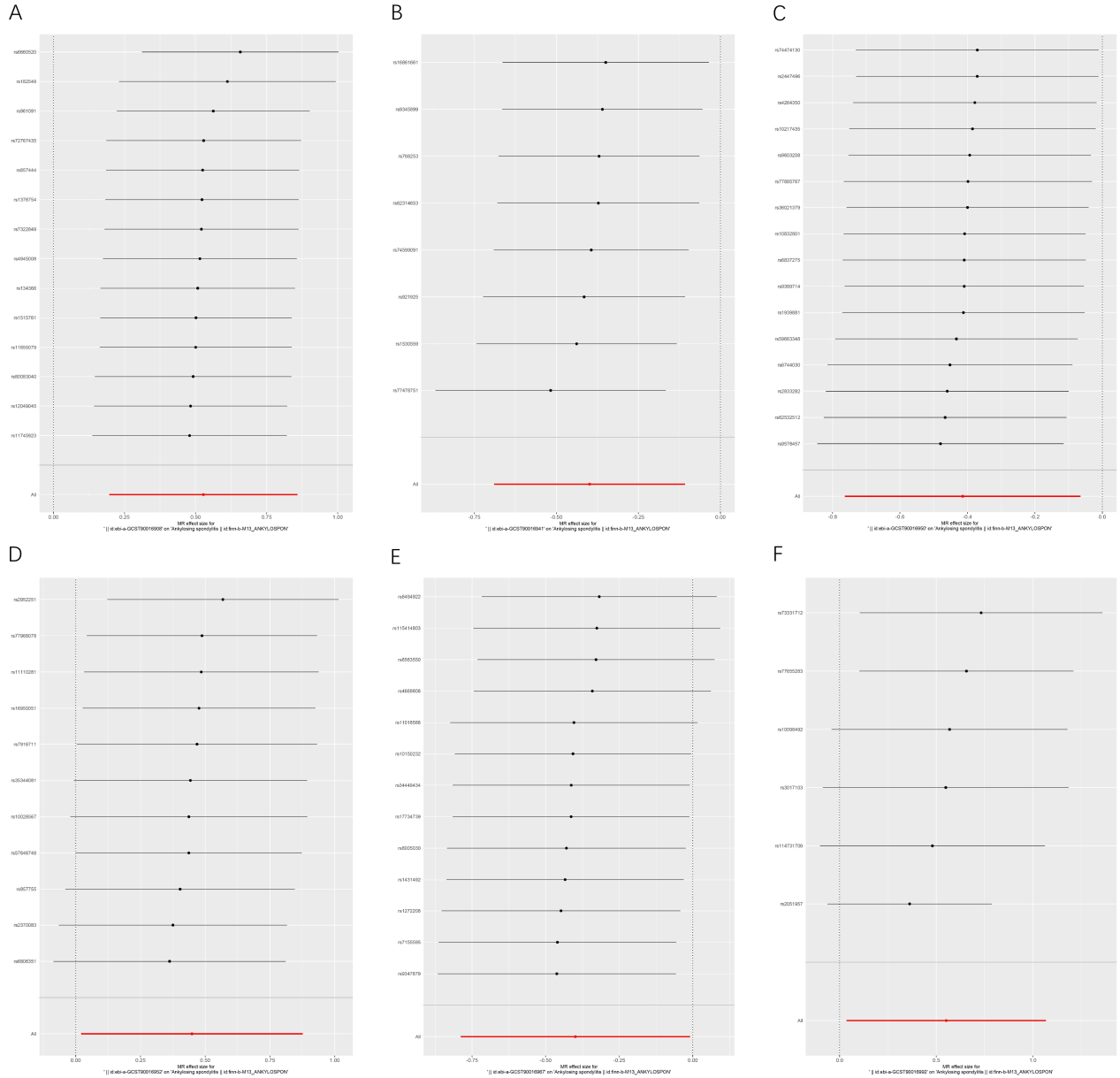
**

**
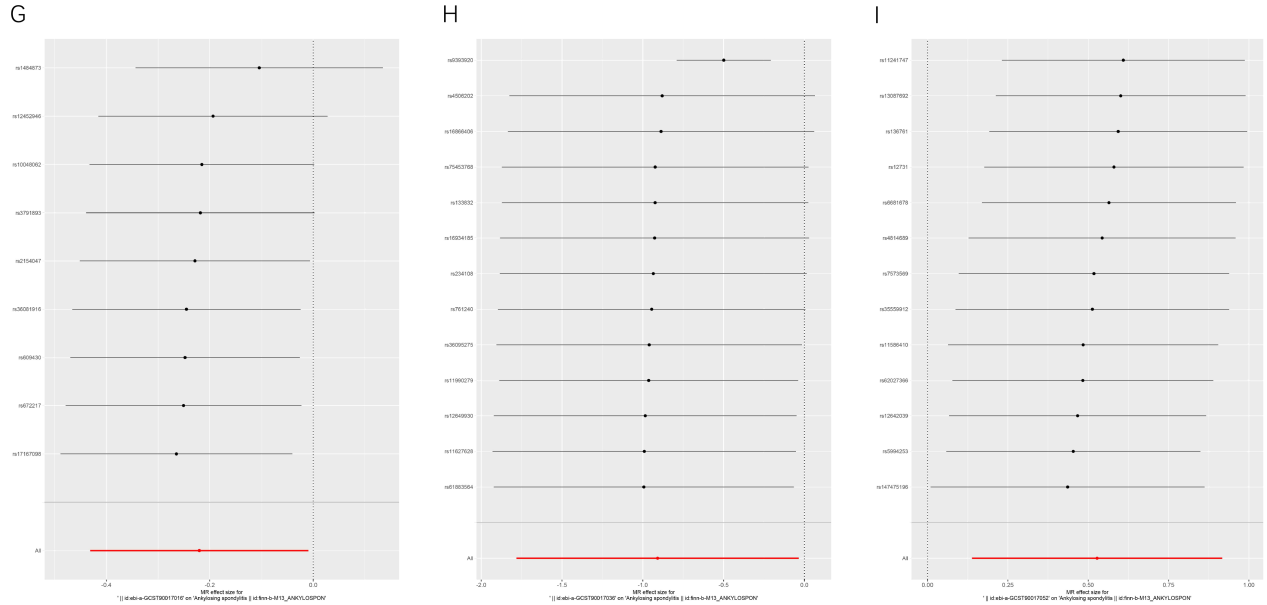
**

Supplement Figure S27

Leave-one-out plots of significant and nominal significant estimates from genetically predicted gut microbiota on Ankylosing spondylitis. (A)class Actinobacteria; (B)family Lactobacillaceae; (C)family Rikenellaceae; (D)family Streptococcaceae; (E)genus Anaerotruncus; (F)genus Enterorhabdus; (G)genus Howardella; (H)genus Oscillibacter; (I)genus Ruminococcaceae NK4A214 group.

**
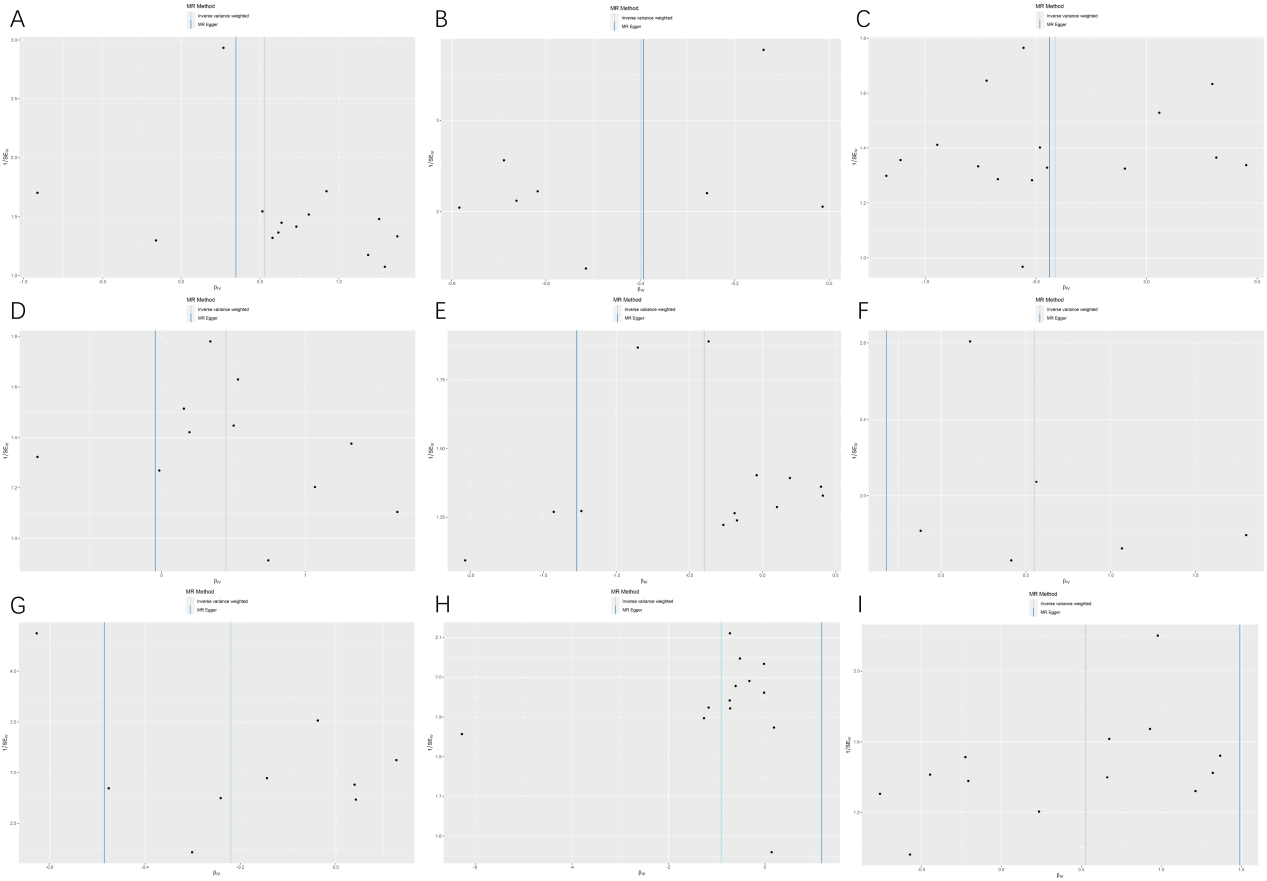
**

Supplement Figure S28

Funnel plots of significant and nominal significant estimates from genetically predicted

gut microbiota on Ankylosing spondylitis. (A)class Actinobacteria; (B)family Lactobacillaceae; (C)family Rikenellaceae; (D)family Streptococcaceae; (E)genus Anaerotruncus; (F)genus Enterorhabdus; (G)genus Howardella; (H)genus Oscillibacter; (I)genus Ruminococcaceae NK4A214 group.

**
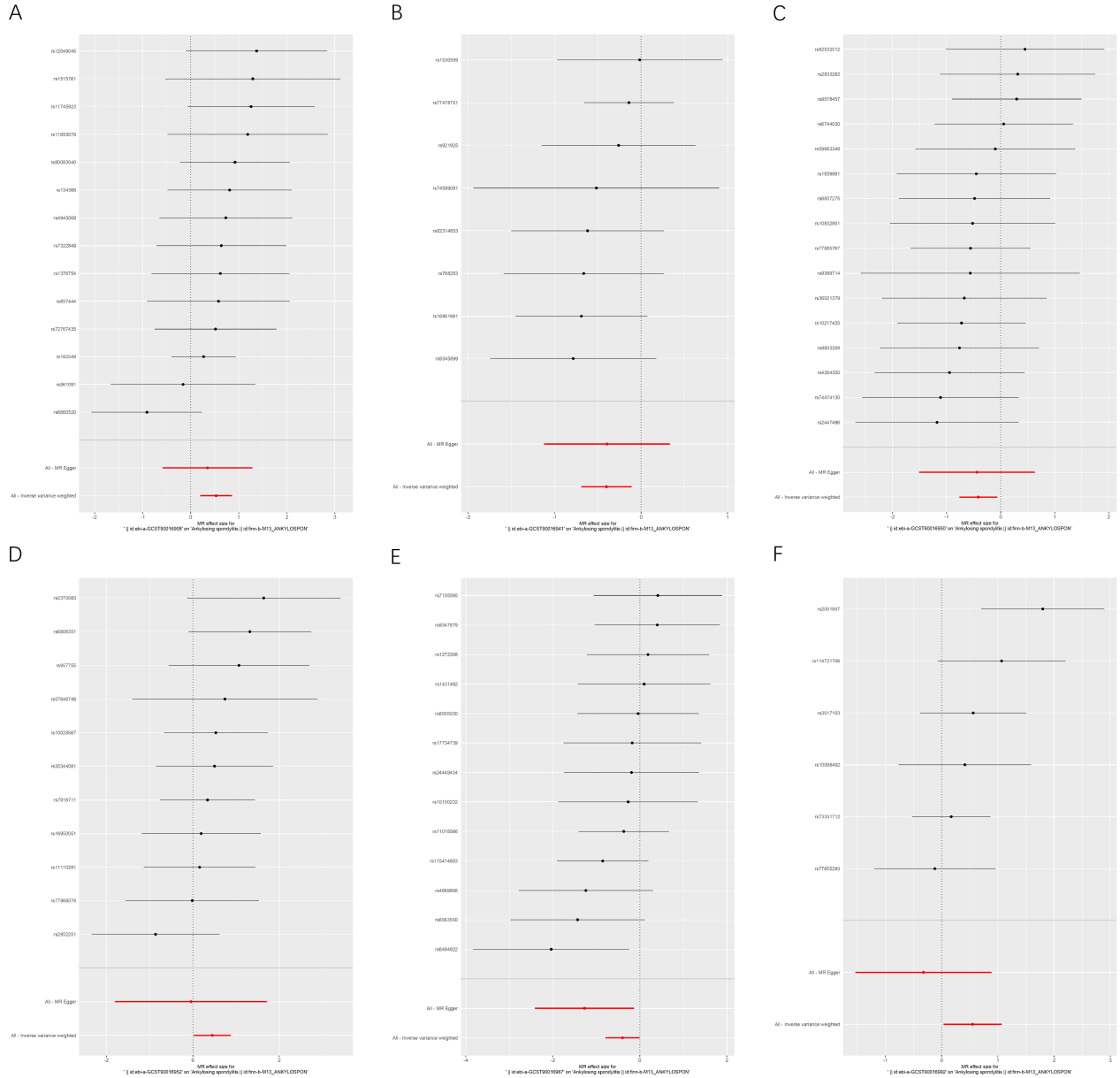
**

**
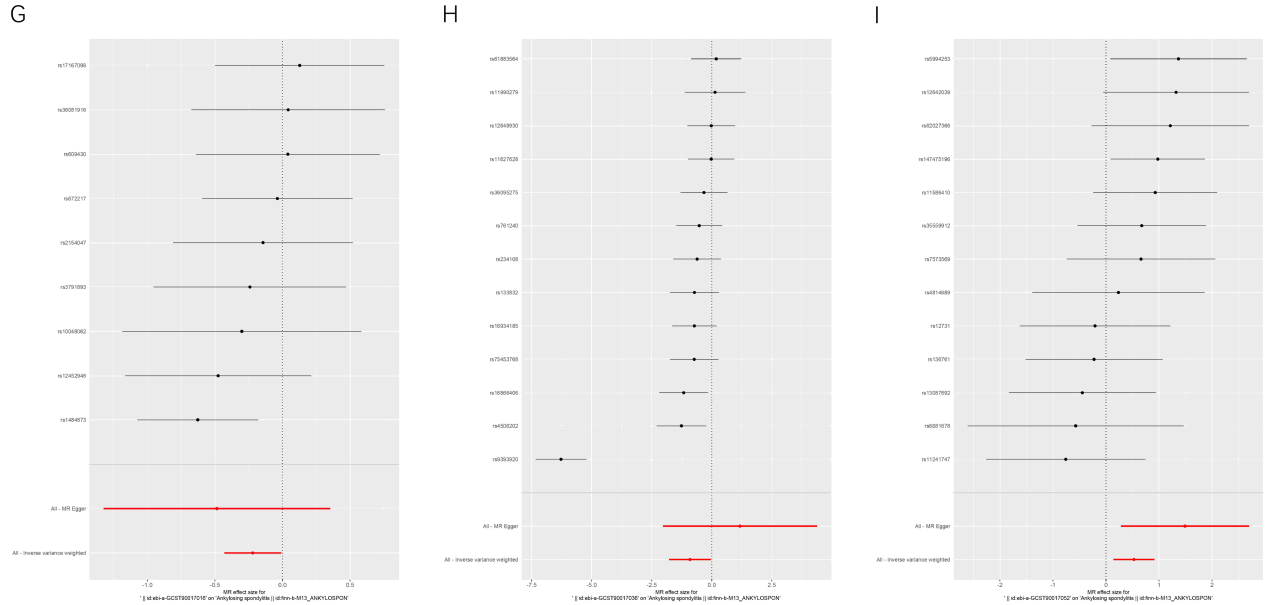
**

Supplement Figure S29

Single plots of significant and nominal significant estimates from genetically predicted

gut microbiota on Ankylosing spondylitis. (A)class Actinobacteria; (B)family Lactobacillaceae; (C)family Rikenellaceae; (D)family Streptococcaceae; (E)genus Anaerotruncus; (F)genus Enterorhabdus; (G)genus Howardella; (H)genus Oscillibacter; (I)genus Ruminococcaceae NK4A214 group.

**
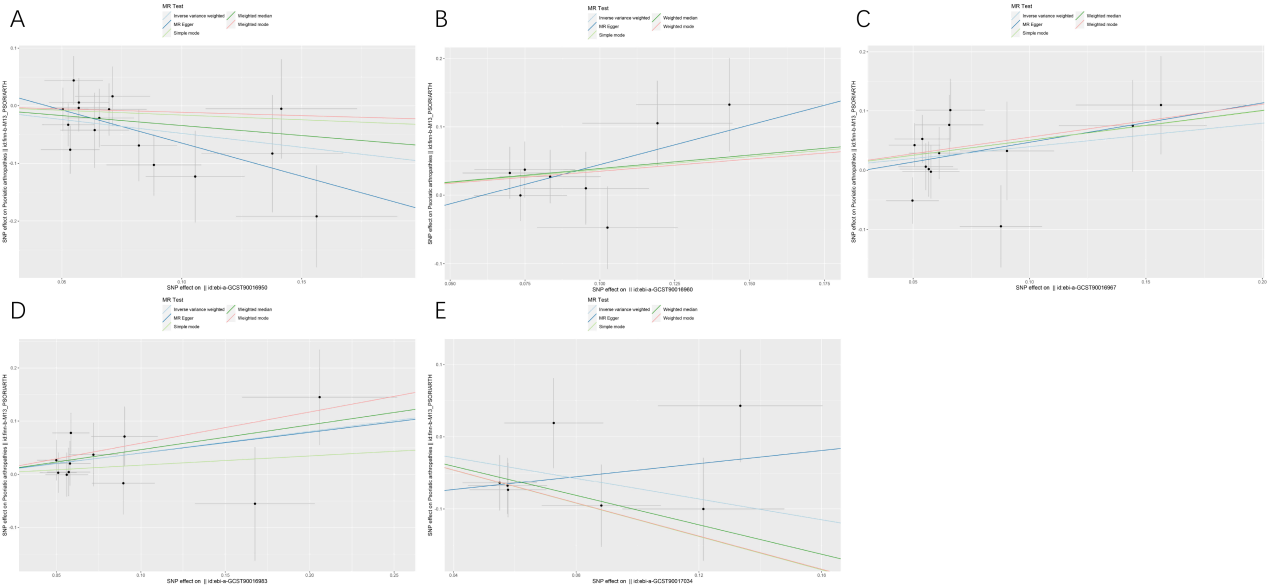
**

Supplement Figure S30

Scatter plots of significant and nominal significant estimates from genetically predicted

gut microbiota on Psoriatic arthropathies. (A)family Rikenellaceae; (B)genus Adlercreutzia; (C)genus Anaerotruncus; (D)genus Coprococcus1; (E)genus Odoribacter.

**
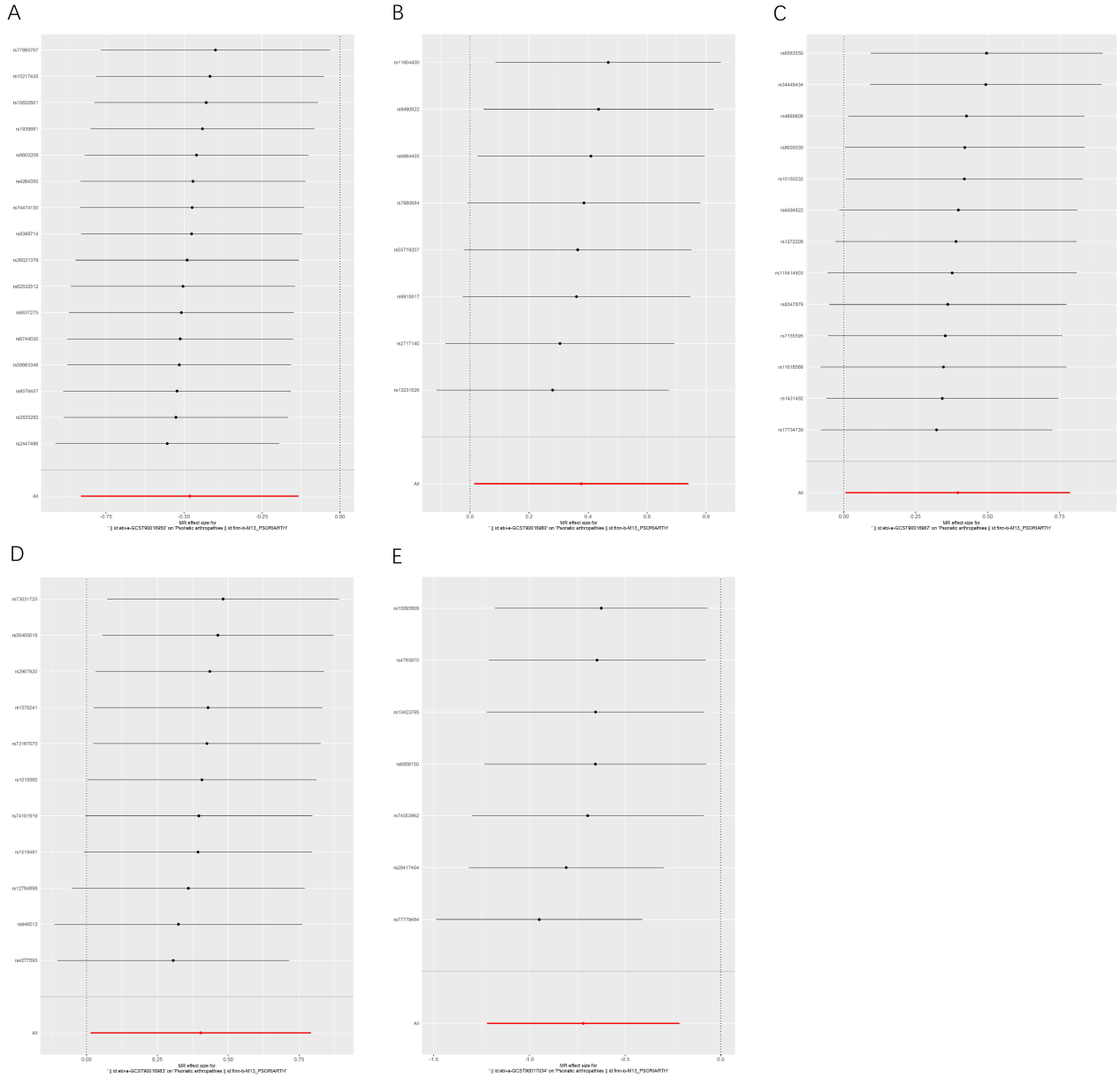
**

Supplement Figure S31

Leave-one-out plots of significant and nominal significant estimates from genetically predicted

gut microbiota on Psoriatic arthropathies. (A)family Rikenellaceae; (B)genus Adlercreutzia; (C)genus Anaerotruncus; (D)genus Coprococcus1; (E)genus Odoribacter.

**
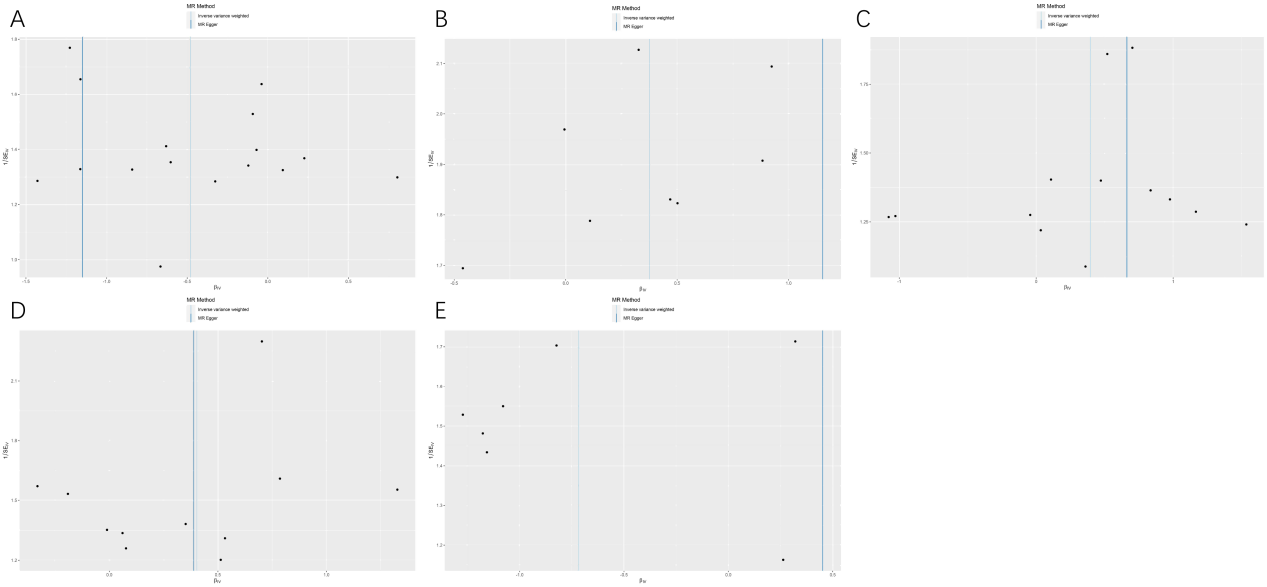
**

Supplement Figure S32

Funnel plots of significant and nominal significant estimates from genetically predicted

gut microbiota on Psoriatic arthropathies. (A)family Rikenellaceae; (B)genus Adlercreutzia; (C)genus Anaerotruncus; (D)genus Coprococcus1; (E)genus Odoribacter.

**
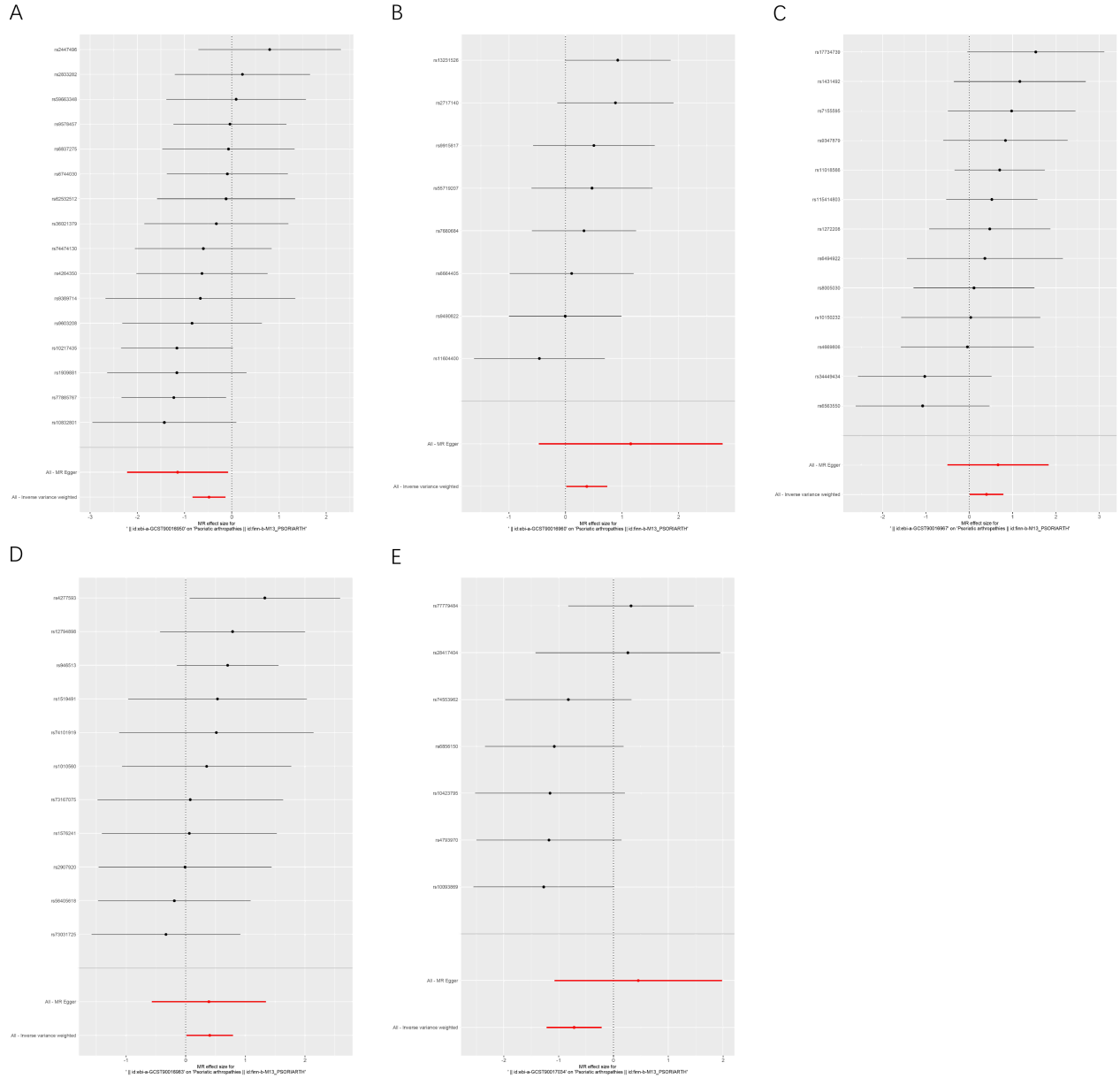
**

Supplement Figure S33

Single plots of significant and nominal significant estimates from genetically predicted

gut microbiota on Psoriatic arthropathies. (A)family Rikenellaceae; (B)genus Adlercreutzia; (C)genus Anaerotruncus; (D)genus Coprococcus1; (E)genus Odoribacter.


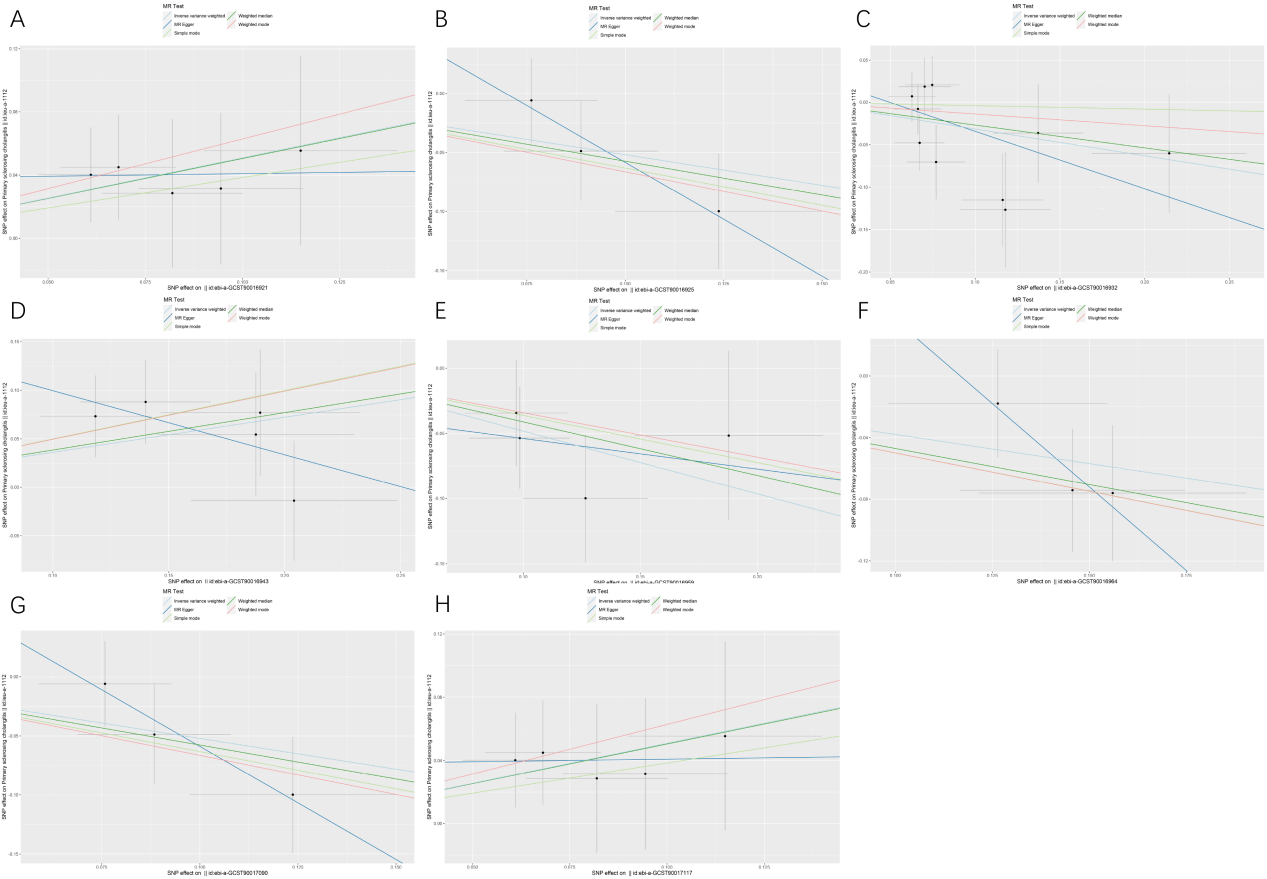


Supplement Figure S34

Scatter plots of significant and nominal significant estimates from genetically predicted

gut microbiota on Primary sclerosing cholangitis. (A)class Mollicutes; (B)family Actinomycetaceae; (C)family Clostridiales vadin BB60 group; (D)family Oxalobacteraceae; (E)genus Actinomyces; (F)genus Alloprevotella; (G)order Actinomycetales; (H)phylum Tenericutes.


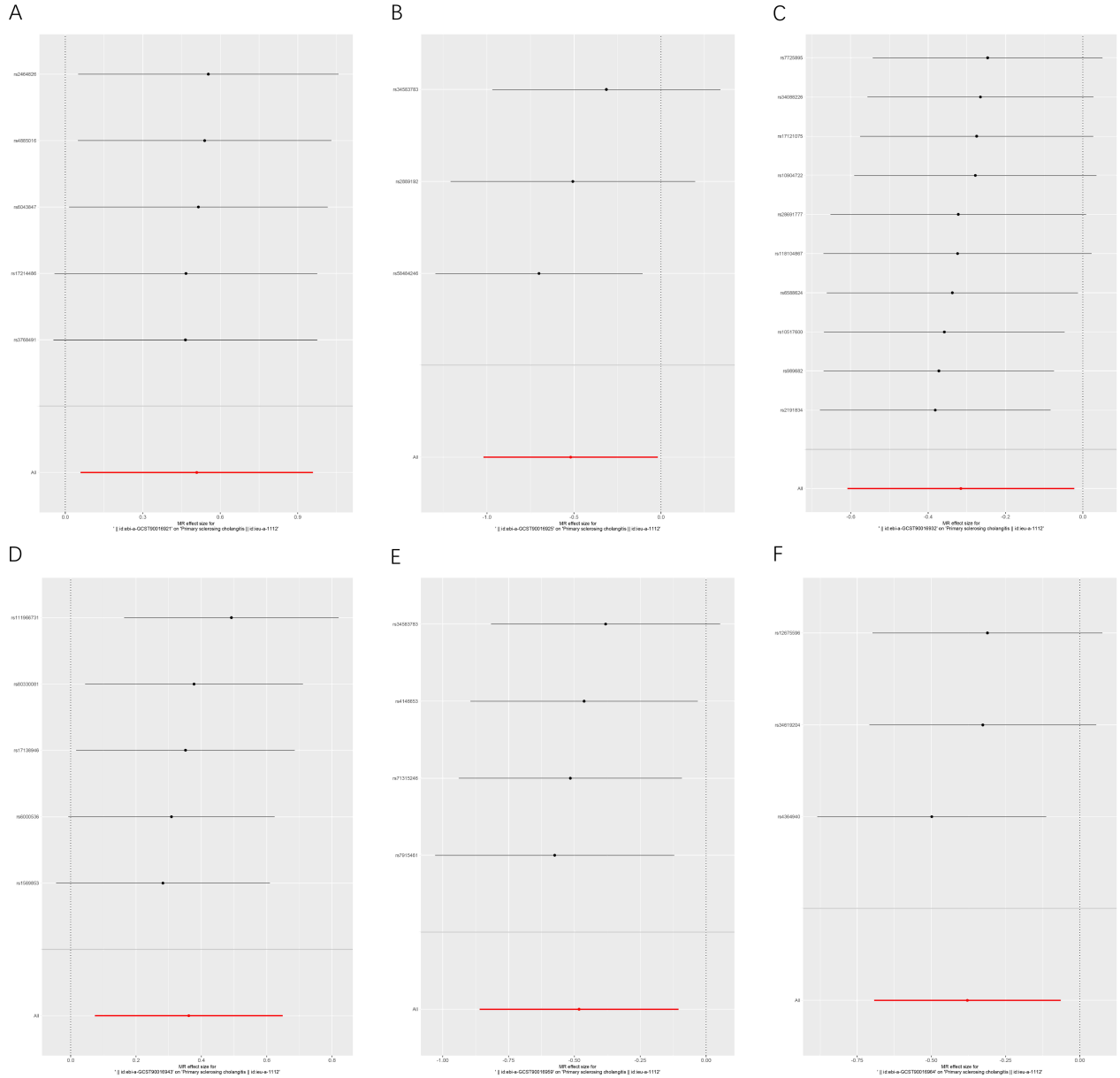


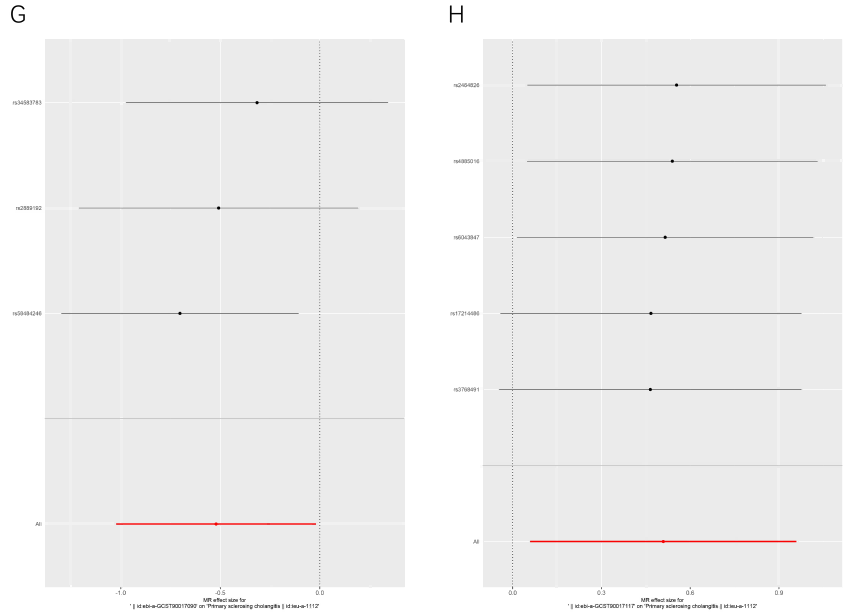


Supplement Figure S35

Leave-one-out plots of significant and nominal significant estimates from genetically predicted

gut microbiota on Primary sclerosing cholangitis. (A)class Mollicutes; (B)family Actinomycetaceae; (C)family Clostridiales vadin BB60 group; (D)family Oxalobacteraceae; (E)genus Actinomyces; (F)genus Alloprevotella; (G)order Actinomycetales; (H)phylum Tenericutes.

**
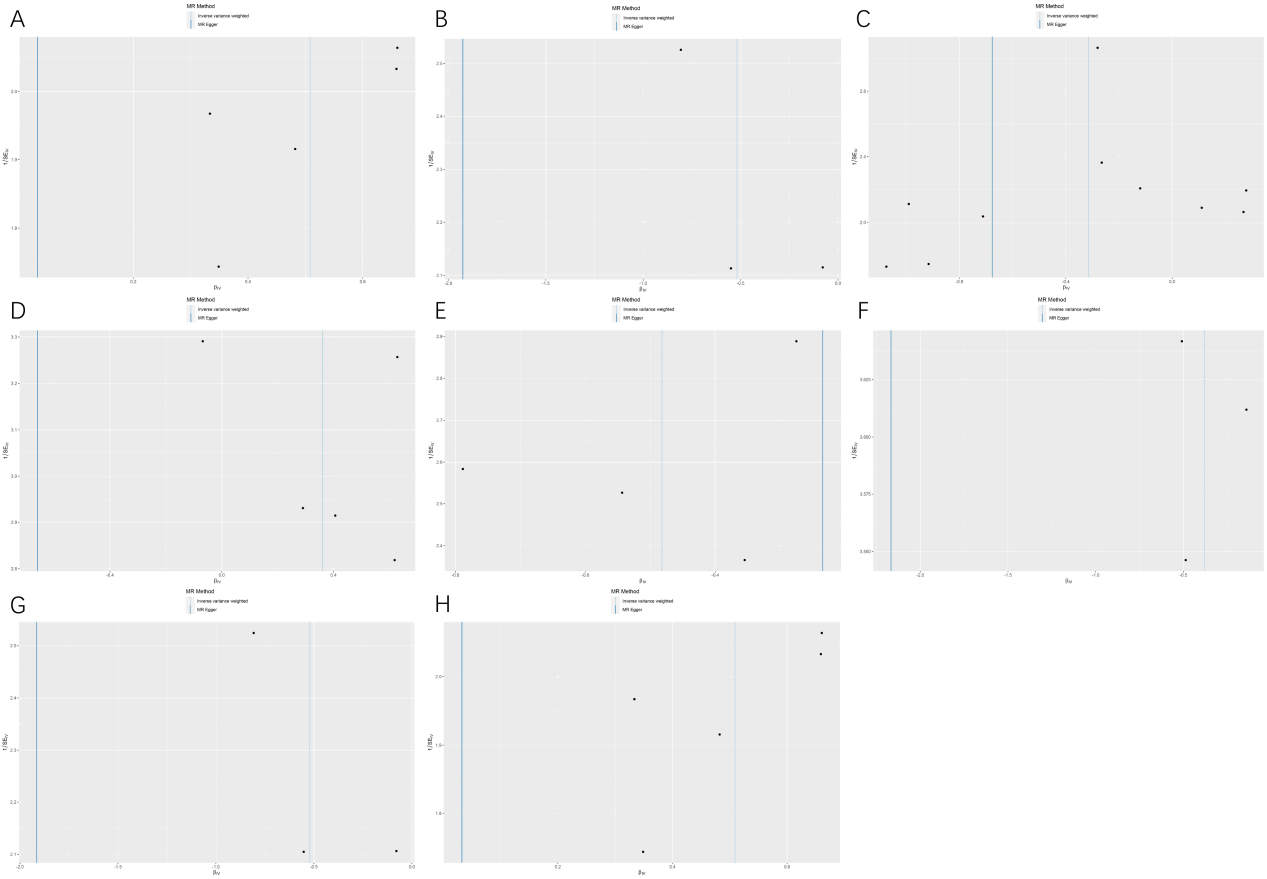
**

Supplement Figure S36

Funnel plots of significant and nominal significant estimates from genetically predicted

gut microbiota on Primary sclerosing cholangitis. (A)class Mollicutes; (B)family Actinomycetaceae; (C)family Clostridiales vadin BB60 group; (D)family Oxalobacteraceae; (E)genus Actinomyces; (F)genus Alloprevotella; (G)order Actinomycetales; (H)phylum Tenericutes.

**
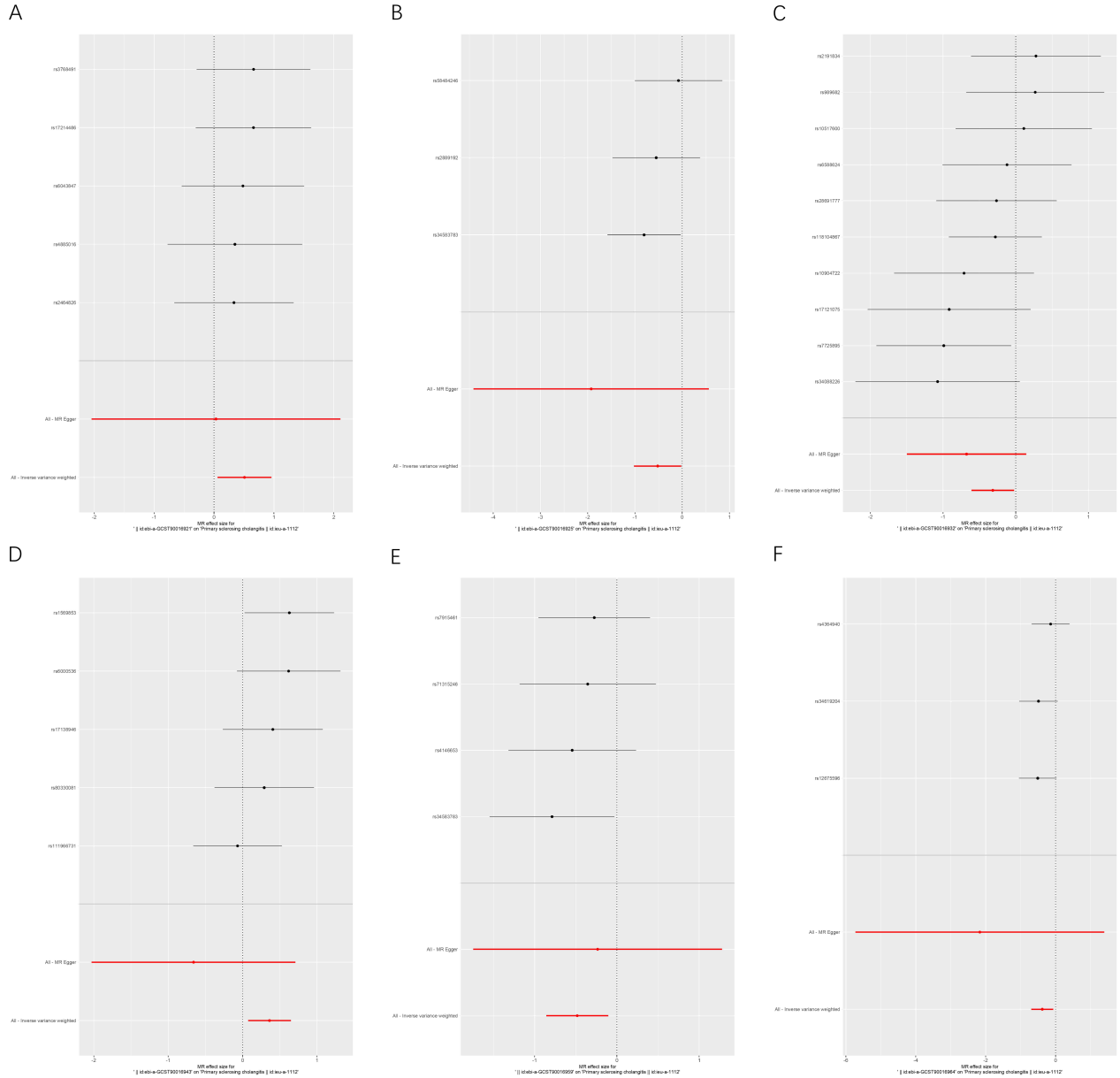
**

**
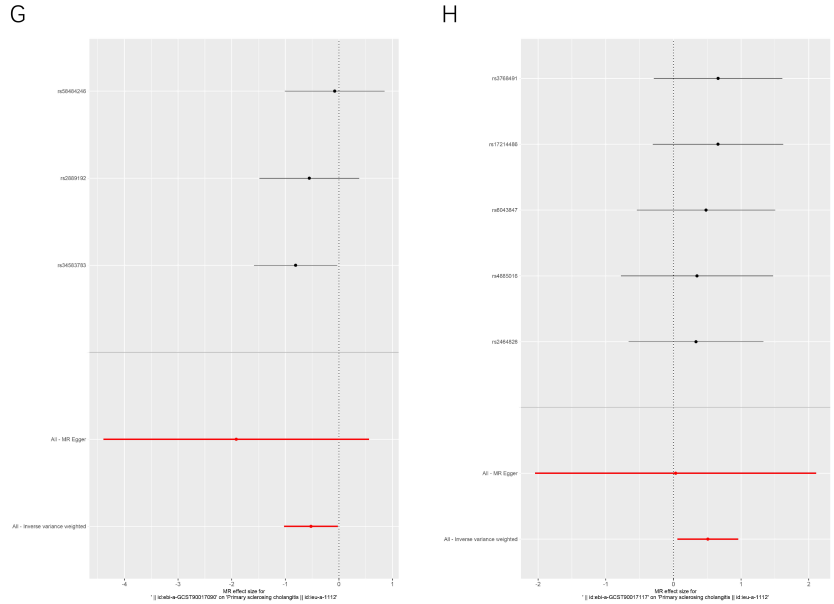
**

Supplement Figure S37

Single plots of significant and nominal significant estimates from genetically predicted

gut microbiota on Primary sclerosing cholangitis. (A)class Mollicutes; (B)family Actinomycetaceae; (C)family Clostridiales vadin BB60 group; (D)family Oxalobacteraceae; (E)genus Actinomyces; (F)genus Alloprevotella; (G)order Actinomycetales; (H)phylum Tenericutes.

**
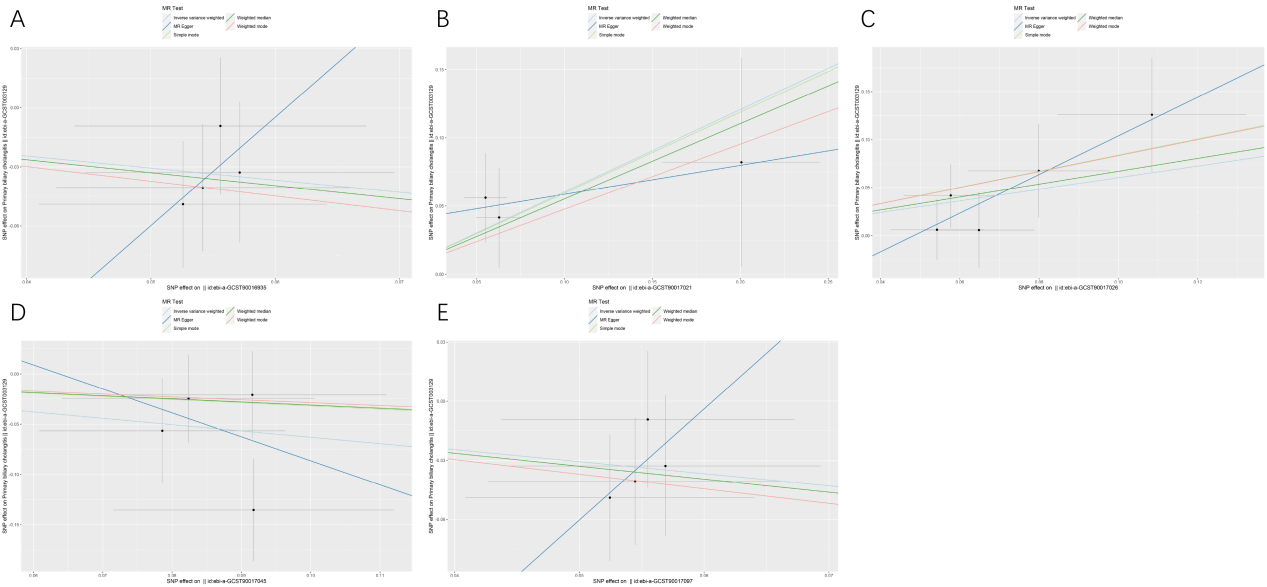
**

Supplement Figure S38

Scatter plots of significant and nominal significant estimates from genetically predicted

gut microbiota on Primary biliary cholangitis. (A)family Desulfovibrionaceae; (B)genus Lachnospiraceae FCS020 group; (C)genus Lachnospiraceae UCG004; (D)genus Prevotella9; (E)order Desulfovibrionales.

**
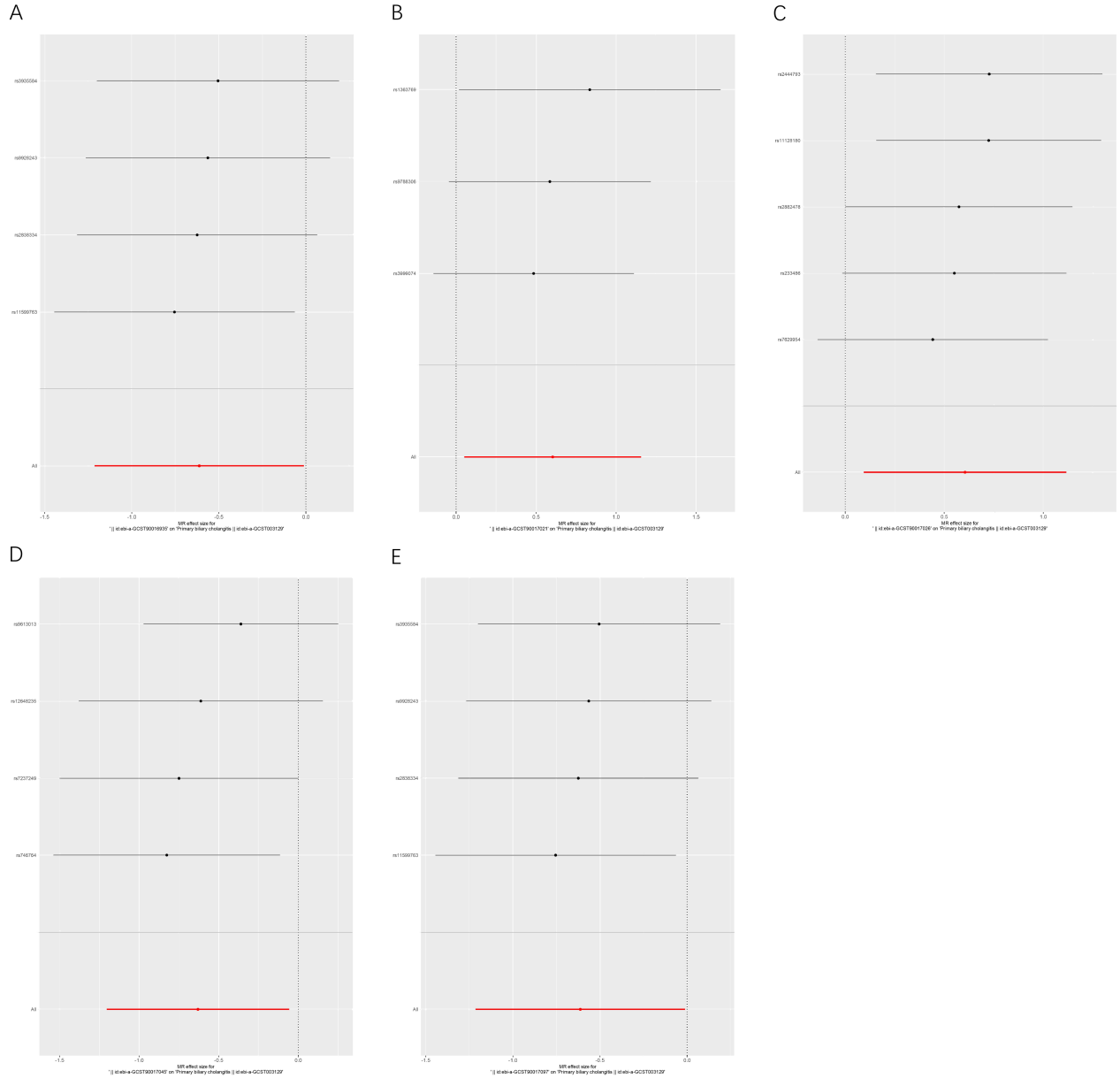
**

Supplement Figure S39

Leave-one-out plots of significant and nominal significant estimates from genetically predicted

gut microbiota on Primary biliary cholangitis. (A)family Desulfovibrionaceae; (B)genus Lachnospiraceae FCS020 group; (C)genus Lachnospiraceae UCG004; (D)genus Prevotella9; (E)order Desulfovibrionales.

**
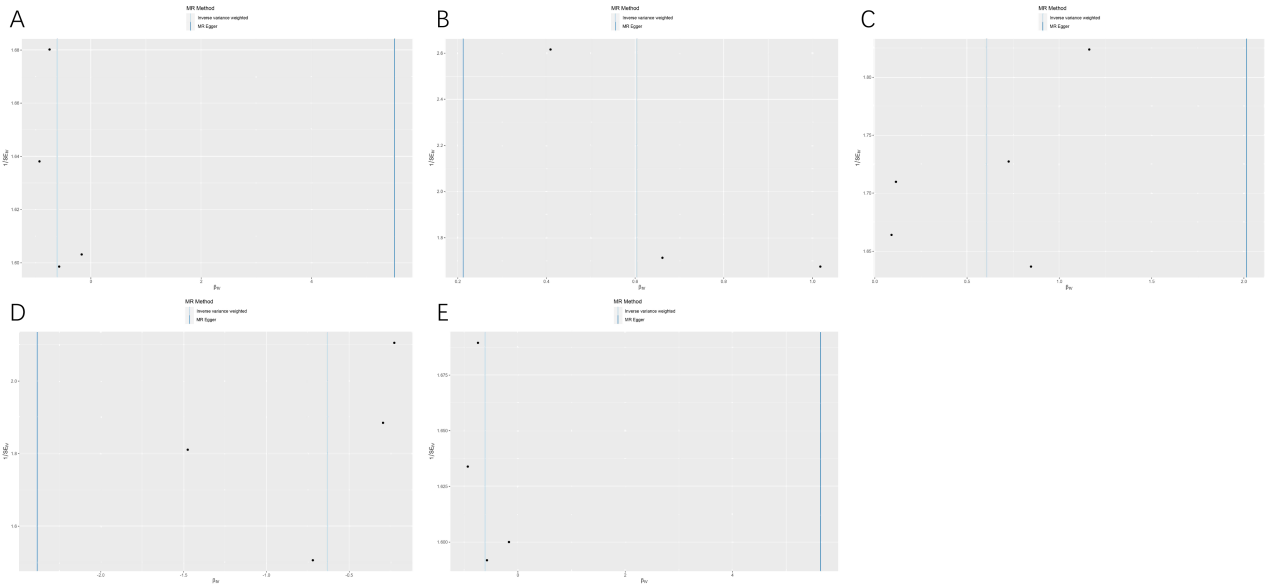
**

Supplement Figure S40

Funnel plots of significant and nominal significant estimates from genetically predicted

gut microbiota on Primary biliary cholangitis. (A)family Desulfovibrionaceae; (B)genus Lachnospiraceae FCS020 group; (C)genus Lachnospiraceae UCG004; (D)genus Prevotella9; (E)order Desulfovibrionales.

**
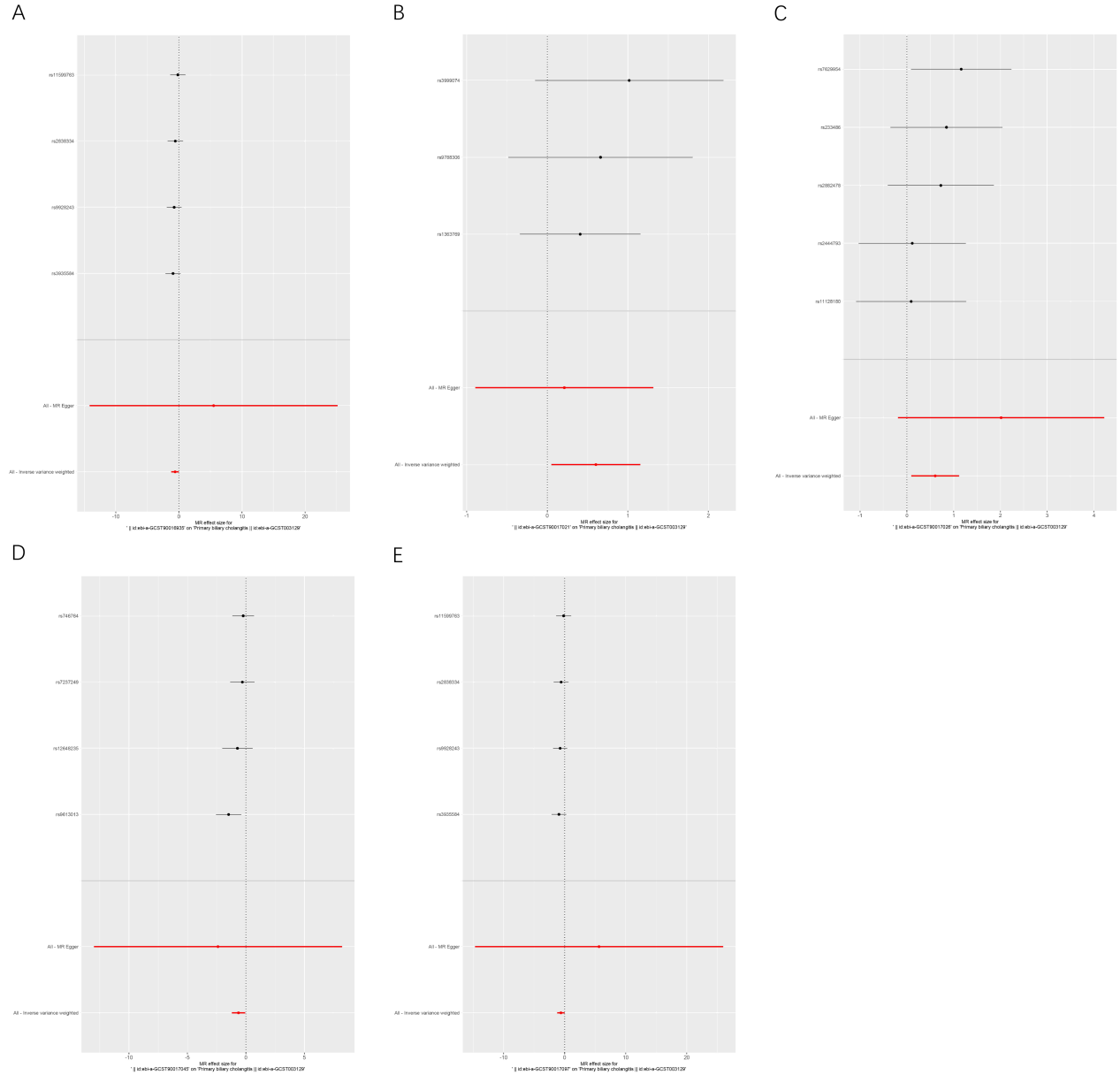
**

Supplement Figure S41

Single plots of significant and nominal significant estimates from genetically predicted

gut microbiota on Primary biliary cholangitis. (A)family Desulfovibrionaceae; (B)genus Lachnospiraceae FCS020 group; (C)genus Lachnospiraceae UCG004; (D)genus Prevotella9; (E)order Desulfovibrionales.
